# Plasma cells are not restricted to the CD27+ phenotype: characterization of CD27-CD43+ antibody-secreting cells

**DOI:** 10.3389/fimmu.2023.1165936

**Published:** 2023-07-10

**Authors:** Kris Covens, Bert Verbinnen, Britt G. de Jong, Leen Moens, Greet Wuyts, Geert Verheyen, Kris Nys, Jonathan Cremer, Stijn Smulders, Rik Schrijvers, Andreas Weinhäusel, Séverine Vermeire, Patrick Verschueren, Ellen De Langhe, Jacques J. M. van Dongen, Menno C. van Zelm, Xavier Bossuyt

**Affiliations:** ^1^ Department of Microbiology and Immunology, Clinical and Diagnostic Immunology Research Group, Leuven, Belgium; ^2^ Biocartis, Research and Development, Mechelen, Belgium; ^3^ Biomedical Laboratory Technology, Radius, Life Sciences and Chemistry, Thomas More Kempen, Geel, Belgium; ^4^ Department of Immunology, Erasmus MC, University Medical Center, Rotterdam, Netherlands; ^5^ Department of Periodontology, ACTA, University of Amsterdam and VU University, Amsterdam, Netherlands; ^6^ Department of Microbiology and Immunology, Inborn Errors of Immunity, Leuven, Belgium; ^7^ Gastroenterology, University Hospitals Leuven, Leuven, Belgium; ^8^ Department of Microbiology and Immunology, Allergy and Clinical Immunology Research Group, Leuven, Belgium; ^9^ AIT Austrian Institute of Technology GmbH, Center for Health and Bioresources, Molecular Diagnostics, Vienna, Austria; ^10^ Department of Rheumatology, University Hospitals Leuven, Leuven, Belgium; ^11^ Department of Immunology, Leiden University Medical Center (LUMC), Leiden, Netherlands; ^12^ Centro de Investigación del Cáncer-Instituto de Biología Molecular y Celular del Cáncer (CIC-IBMCC, USAL-CSIC-FICUS), Salamanca, Spain; ^13^ Department of Medicine, University of Salamanca (USAL), Salamanca, Spain; ^14^ Department of Immunology and Pathology, Central Clinical School, Monash University and Alfred Hospital, Melbourne, VIC, Australia; ^15^ Department of Laboratory Medicine, University Hospitals Leuven, Leuven, Belgium

**Keywords:** antibody secreting cells, CD43, CD27, plasmablast, somatic hypermutation, B lymphocyte

## Abstract

Circulating antibody-secreting cells are present in the peripheral blood of healthy individuals reflecting the continued activity of the humoral immune system. Antibody-secreting cells typically express CD27. Here we describe and characterize a small population of antibody-secreting class switched CD19+CD43+ B cells that lack expression of CD27 in the peripheral blood of healthy subjects. In this study, we characterized CD27-CD43+ cells. We demonstrate that class-switched CD27-CD43+ B cells possess characteristics of conventional plasmablasts as they spontaneously secrete antibodies, are morphologically similar to antibody-secreting cells, show downregulation of B cell differentiation markers, and have a gene expression profile related to conventional plasmablasts. Despite these similarities, we observed differences in IgA and IgG subclass distribution, expression of homing markers, replication history, frequency of somatic hypermutation, immunoglobulin repertoire, gene expression related to Toll-like receptors, cytokines, and cytokine receptors, and antibody response to vaccination. Their frequency is altered in immune-mediated disorders.

**Conclusion:**

we characterized CD27-CD43+ cells as antibody-secreting cells with differences in function and homing potential as compared to conventional CD27+ antibody-secreting cells.

## Introduction

Long-term protective humoral immunity depends on the generation and survival of antibody-secreting cells (ASC). During an immune response, B cells are activated in secondary lymphoid organs and differentiate into antibody-secreting plasmablasts that are the precursors of long-lived plasma cells (1). The majority of these plasma cells home to the bone marrow or the lamina propria of the gut after a transient passage in the peripheral blood (2). The permanent presence of circulating ASC in healthy individuals reflects the continued activity of the humoral immune system (3, 4).

In the absence of obvious infection or vaccination, most circulating ASC express IgA (3, 5). Expression of specific homing receptors (CCR10, CCR9, β7-Integrin) on these steady-state circulating ASC suggests that they are derived from mucosal immune reactions and/or can home to mucosal tissue (3). In response to vaccination or during infection, additional ASC transiently circulate in the peripheral blood and transit to the bone marrow or inflamed tissue (3). The kinetics of the appearance of antigen-specific ASC in the peripheral blood invariably peaks at day 6-7 after immunization or infection, and the response seems to be independent of the type of antigen, the adjuvant used, and the route of immunization (3, 6–18). A large part of these ASC is IgG-class-switched, likely because non-mucosal lymphoid organs participate in these responses (3). For example, ASC generated in response to vaccination with tetanus toxoid mostly secrete IgG, lack expression of mucosal homing receptors, express CXCR4, and home in on the bone marrow (3, 19). A memory response to viral pathogens generated IgG and IgA ASC (20), whereas a natural dengue infection induced specific IgA, IgG, and IgM ASC (13).

ASC are characterized by the downregulation of CD20, expression of CD38, and up-regulation of CD27 (21). CD27 is also a hallmark of memory B cells (Bmem). Although CD27 was initially used as a marker to identify memory B cells (Bmem), CD27- B cells have been described as exhibiting classic attributes of memory cells, including upregulated activation markers, extensive replication history, and somatically mutated Ig genes (22–24). In addition to identifying certain subsets of Bmem, CD27 is generally assumed to be highly up-regulated on ASC (10).

In previous work, we showed that conventional ASC express high levels of CD43 and that CD43 expression can be induced upon activation in distinct memory B cell subsets (25). These data are in line with CD43 as a B cell activation marker (26). Furthermore, we consistently observed the presence of a small population of CD43+ cells within the CD19+CD27- B cell fraction in the peripheral blood of healthy subjects. Here we show that this is a new population of circulating ASC lacking CD27.

## Methods

### Donors and samples

Anonymous peripheral blood samples were obtained as buffy coats from the Flemish Red Cross. Healthy adult volunteers received Rabipur (GlaxoSmithKline), Pneumo 23 (Sanofi Pasteur), pneumovax (Sanofi Pasteur) or Tedivax pro-Adulto (GlaxoSmithKline), and blood was collected by venipuncture using Heparin as anticoagulant 7 or 10 days later. The study was approved by the Ethics Committee of the University Hospitals of Leuven (S52146). Participants in the vaccination and immunophenotyping studies gave written informed consent (S52146, S63708).

### Processing

All samples were processed promptly upon receipt. Peripheral blood mononuclear cells (PBMC) were isolated from buffy coats or whole blood by density gradient separation (Lucron Biopruducts, De Pinte, Belgium). For sorting purposes, B cells were isolated using CD19+ beads (Miltenyi Biotec, Mönchengladbach, Germany) as per the manufacturer’s instructions. Cells were incubated in RPMI1640 (Life Technologies, Merelbeke, Belgium) containing 10% fetal calf serum (FCS) (Hyclone, Geel, Belgium) plus 2 mM L-glutamine, and 50 µg/ml gentamicin (Life Technologies).

### Antibodies, flow cytometry, and cell sorting

**Table S1** contains a complete list of all fluorochrome-conjugated antibodies (and viability dye) used in this study. All antibodies were titrated for optimal resolution between positive and negative populations and used at their optimal concentration. Surface staining of cells was performed in phosphate-buffered saline (PBS) with 0.5% bovine serum albumin (BSA) and 2 mM EDTA for 30 minutes at 37°C in the dark. Subsequently, cells were washed with PBS and either fixed for 30 minutes in PBS with 2% formaldehyde and subsequently stored at 4°C in the dark in PBS with 0.5% BSA and 2 mM EDTA (for analysis), or resuspended in PBS with 0.5% BSA and 2 mM EDTA for sorting. For intracellular staining, cells were permeabilized with PBS containing 0.5% BSA and 2 mM EDTA, and 0.5% Tween-20 (perm-buffer) for 30 minutes at room temperature in the dark. Subsequently, cells were washed with perm-buffer and intracellular staining (for IgA and IgG) was performed in perm-buffer for 30 minutes at 37°C. Cells were then washed twice with perm-buffer and once with PBS, resuspended in PBS with 0.5% BSA and 2 mM EDTA, and analyzed immediately. Intracellular staining was performed for IgA and IgG in order to correctly classify secreting cells. It was not performed for cell sorting experiments.

Flow cytometry was performed on an LSRFortessa (BD Biosciences, Erembodegem, Belgium), and cell sorting was performed on a FACSAriaI (BD Biosciences). Compensations were set using BD CompBeads (BD Biosciences) for mouse monoclonal antibodies and UltraComp eBeads (eBioscience, Vienna, Austria) for rat monoclonal antibodies. Heat-killed cells (30 minutes at 65°C) were used for setting compensations for the viability dye. Gates were set using fluorescence minus one control (27). An overview of the gating strategy used throughout this study is shown in **Figure S1**.

### ELISPOT

Individual wells of a nitrocellulose Millititer HA plate (MAHANS 4550, Merck Millipore, Overijse, Belgium) were coated overnight at 4°C with 5 µg/ml of tetanus anatoxine (Merck Millipore), mouse anti-human IgA (BD Biosciences), goat anti-human IgG (Life Technologies), or poly-L-lysine hydrobromide (MW 30.000-70.000; Sigma) coupled capsular polysaccharides of *Streptococcus pneumoniae*. After coating, the plates were blocked with PBS containing 1% milk powder for 2 hours at 37°C. Subsequently, cells were cultured overnight in 200 µl RPMI supplemented with 10% FCS and 50 µM β-mercaptoethanol (Life Technologies). After washing five times with PBS-0.05% Tween20, the wells were incubated with 100 µl of biotinylated goat anti-human IgA, or goat anti-human IgG (both from ImTec Diagnostics, Antwerp, Belgium). After 2 hours of incubation at 37°C, wells were washed with PBS and peroxidase-conjugated streptavidin (Jackson Immunoresearch Laboratories, West Grove, PA) was added and incubated for 1 hour at 37°C. Spots were visualized using 3-amino-9-ethylcarbazole (Sigma-Aldrich, Bornem, Belgium). After spots appeared, the reaction was stopped with tap water. Analysis was performed using Eli. Analysis software (A.EL.VIS, Hannover, Germany). Purified rabies-specific G glycoprotein antigen [obtained through collaboration with J. McGettigan, Thomas Jefferson University, Philadelphia (USA)] was used for the ELISPOT analysis.

### Replication history analysis using the KREC assay

DNA was isolated from each sorted subset with the QIAamp DNA micro kit (QIAgen, Venlo, The Netherlands). The replication history of sorted B-cell subsets was determined with the Igκ-deleting recombination excision circles (KREC) assay as described previously (28). Briefly, the amounts of coding and signal joints of the IGK-deleting rearrangement were measured by real-time quantitative PCR in DNA from sorted B-cell populations on a StepOnePlus Real-Time PCR (Thermo Fisher Scientific, Waltham, MA). Signal joints, but not coding joints are diluted 2-fold with every cell division. To measure the number of cell divisions undergone by each population, we calculated the ratio between the number of coding joints and signal joints. The previously established control cell line U698 DB01 (InVivoScribe) contains 1 coding and 1 signal joint per genome and was used to correct for minor differences in the efficiency of both real-time quantitative-PCR assays.

### Sequence analysis of complete IGH gene rearrangements and Ig switch regions

RNA was isolated from each sorted subset with the Absolutely total RNA nanoprep kit (Agilent Technologies, Zaventem, Belgium). After reverse transcription using random hexamers and the Superscript VILO cDNA Synthesis Kit (Life Technologies), IGA and IGG transcripts were amplified using six IGHV-FR1 forward primers in combination with an IGHA (5′GTGGCATGTCACGGACTTG 3′) or an IGHG (5′CACGCTGCTGAGGGAGTAG 3′) consensus reverse primer (29, 30). All PCR products were cloned into the pGEM-T easy vector (Promega, Madison, WI) and prepared for sequencing on the ABI Prism 3130 XL fluorescent sequencer (Life Technologies). Obtained sequences were analyzed with the IMGT database (http://imgt.cines.fr/) to assign the IGHV, IGHD, and IGHJ genes, and to identify somatic mutations. From each unique clone, the mutation frequency was determined within the IGHV gene, as was the length and composition of the IGH-CDR3. Kyte en Doolittle hydrophobicity index was applied to the CDR3 regions. The IgA and IgG receptor subclasses were determined using the IGH reference sequence (NG_001019).

### Comparative transcriptome analysis

Total RNA was isolated from each sorted subset with the Absolutely total RNA nanoprep kit. RNA concentration and purity were determined spectrophotometrically using the Nanodrop ND-1000 (Nanodrop Technologies) and RNA integrity was assessed using a Bioanalyser 2100 (Agilent). Per sample, an amount of 0.5 or 2 ng of total RNA was amplified and converted to cDNA using the NuGEN Ovation Pico WTA System v2.0. Subsequently, the cDNA was fragmented and biotin-labeled using the NuGEN Encore Biotin Module. All steps were carried out according to the manufacturer’s protocol (NuGEN). A mixture of purified and fragmented biotinylated cDNA and hybridization controls (Affymetrix) was hybridized on Affymetrix PrimeView Human Gene Expression arrays followed by staining and washing in a GeneChip^®^ fluidics station 450 (Affymetrix) according to the manufacturer’s procedures. To assess the raw probe signal intensities, chips were scanned using a GeneChip^®^ scanner 3000 (Affymetrix).

Normalization and analysis of the transcriptome data were performed in R version 3.0.1 (31). The gene expression data were normalized using the RMA procedure as implemented in the Affy package (32). The lmFit function contained in the R-package LIMMA (33) was used for the analysis of differential expression among the groups. To correct for multiple comparisons, adjusted P-values (Adj. P-Val) were obtained by a Benjamini-Hochberg correction of the p-values. The genes included (i) 19 established human markers of late B cell differentiation and (ii) 253 human homologs of murine ASC-related genes (34, 35). The genes are shown in **Table S2**.

When indicated, multigroup comparison (ANOVA) and clustering were performed by Qlucore Omics Explorer 3.8 (Qlucore AB 2021).

### Patients

All subjects were recruited at the inflammatory bowel disease (IBD) out-patient clinic of the University Hospitals of Leuven (Belgium), from a control group of unrelated healthy controls, at the general internal medicine department of the University Hospitals Leuven (CVID patients) and at the department of Rheumatology of the University Hospitals Leuven (patients with rheumatoid arthritis and with systemic lupus erythematosus). Healthy volunteers (n=10) were 40% female with a median age of 28 years old, ulcerative colitis (UC) patients (n=10) were 60% female with a median age of 31.5 years old, Crohn’s disease (CD) patients (n=11) were 72.7% female with a median age of 33 years old, and CVID patients (n=5) were 60% female with a median age of 44 years (range 19-54). Rheumatoid arthritis patients (n=7) were 71% female with a median age of 50.6 (range 31-79). Systemic lupus erythematosus patients (n=6) were 83%% female with a median age of 38 (range 28-48). Diagnosis of active UC/CD was confirmed based on a combination of clinical, endoscopic, radiologic, histological, and biochemical criteria according to existing guidelines (36, 37), CVID diagnosis was based on ESID criteria (http://esid.org/Working-Parties/Registry/Diagnosis-criteria). Ethical approval was given by the Ethics Board of the University Hospital Leuven (B322201213950/S53684). Informed consent was obtained from all participants. Basic demographic data and clinical data at the time of blood sampling were extracted from the clinical patient files.

### High-density protein microarray

#### Sample preparation

PBMCs were separated from whole blood by Ficoll-Paque density gradient centrifugation. The blood was obtained from three healthy blood donors through the Red Cross-Flanders. CD19 B cells were isolated from PBMCs using CD19 magnetic beads (Miltenyi Biotech) and stained [CD3/CD14/CD56_PEVio770 (Miltenyi Biotech), CD43_PE (BD), CD27_APC (BD)] for sorting on BD Aria III. B cells were isolated from PBMCs using CD19 magnetic beads (Miltenyi Biotech) and stained [CD3/CD14/CD56_PEVio770 (Miltenyi Biotech), CD43_PE (BD), CD27_APC (BD)] for sorting on BD Aria III. In total 50.10^3^ sorted CD43+CD27-, CD43+CD27+, CD43-CD27- and CD43-CD27+ B cells were cultured in 125 µL RPMI supplemented with 10% FCS with or without 1 µg/mL CpG_2006 (Invivogen).

The concentration of the IgG in the supernatant was 8083 ± 2775 ng/mL, 150 ± 89 ng/mL, 766 ± 327 ng/mL, and 48 ± 33 ng/mL for CD43-CD27+, CD43+CD27-, CD43+CD27+ and CD43-CD27- cells, respectively. The concentration of IgA was, respectively, 10743 ± 11284 ng/mL, 436 ± 407 ng/mL, 1106 ± 935 ng/mL, and 84 ± 51 ng/mL. The concentration of IgM was, respectively, 22033 ± 10027 ng/mL, 4890 ± 4339 ng/mL, 324 ± 48 ng/mL, and 632 ± 537 ng/mL. The total volume of supernatant produced was 4 mL, 5.6 mL, 0.09 mL, and 0.2 mL for CD43-CD27+, CD43+CD27-, and CD43+CD27+ and CD43-CD27- cells, respectively. The supernatants of CD43-CD27+ and CD43+CD27- cells were concentrated. Samples were stored at -80°C until further utilization on protein microarray.

#### Protein microarray analysis

The 16k protein microarray was printed at the Austrian Institute of Technology and comprised 7390 annotated human proteins, corresponding to 6258 different genes. The recombinant proteins (each represented by 2 to 3 clones) were derived from full-length E. Coli cDNA expression clones [the UniPex expression libraries (human fetal brain, T-cell, lung- and colon expression libraries)]. For a detailed description of the 16K protein microarray platform, see (38).

The concentrated cell culture supernatants (200 µL) were thawed on ice and diluted 1:2 with 200 µL 2X assay buffer (2 x PBS pH 7.4 with 0.1% Triton X-100 and 1.5% non-fat milk powder). The IgG concentration was 72 µg/mL, 32 µg/mL, and 72 µg/mL (mean: 59 µg/mL ± 19 µg/mL) for the CD43-CD27+ population and 1.81 µg/mL, 0.42 µg/mL and 0.8 µg/mL (1 µg/mL ± 0.6 µg/mL) for the CD43+CD27- population. DMEM (Dulbecco’s Modified Eagle Medium) was used as blank. DMEM spiked with IgG pooled from 10 healthy individuals was used as a positive control. Protein microarray slides were blocked with DIG Easy Hyb solution (Roche, Basel, Swiss) followed by three washing steps in wash buffer (1 x PBS 0.1% Triton X-100) for 5 minutes each. Rinsing was done with ultrapure water and spin drying (900 rpm, 4 min). The arrays were incubated for 16 h with gentle rotation (12 rpm) at room temperature in a microarray hybridization oven (Agilent). After hybridization, the slides were washed three times with wash buffer, rinsed with ultrapure water and spin-dried. Thereafter, the slides were incubated for 1 h with Alexa Fluor^®^ 647 goat anti-human IgG detection antibody (Invitrogen, Life Technologies) in 1 x PBS pH 7.4 with 0.1% Triton X-100 and 3% non-fat dry milk powder). Image acquisition was done with the Tecan PowerScannerTM. After scanning, a second detection using a 1:7500 diluted Alexa Fuor 647-conjugated Affine Pure Rabbit Anti-bovine IgG (in 1 x PBS pH 7.4 with 0.1% Triton X-100 and 3% non-fat dry milk powder) was performed. This latter step was performed to exclude any influence of bovine IgG which could be present in the supernatant of the cell culture. Only human-specific antibodies were considered for further analysis.

#### Data acquisition and statistical analyses

Fluorescence intensities from the scanned array images were calculated using GenePix Pro Microarray Acquisition and Analysis Software 6.0 (Molecular Devices, Sunnyvale, CA, USA). The local background was subtracted from the median values before statistical data analysis, which was performed using R 2.10.0 and BRB-Array Tools 4.2.1 (https://linus.nci.nih.gov/BRB-ArrayTools.html). Differentially reactive antigens were determined using class comparison analyses (BRB-Array Tools) at the significance thresholds for univariate tests of p ≤ 0.05 (two-sample T-test with random variance model) and minimum fold changes of 1.5 between groups. Average spot intensities of duplicate spots were used for calculations and data were median normalized against the microarray data of IgG pooled from 10 healthy individuals as a reference.

### Statistical analyses

Kruskal-Wallis test followed by a Dunns multiple comparison and Mann Whitney U test was performed by GraphPad Prism. A χ2 test was performed by Analyse-it for Microsoft Excel.

## Results

### CD27- ASC are present in peripheral blood

**Figure 1:**
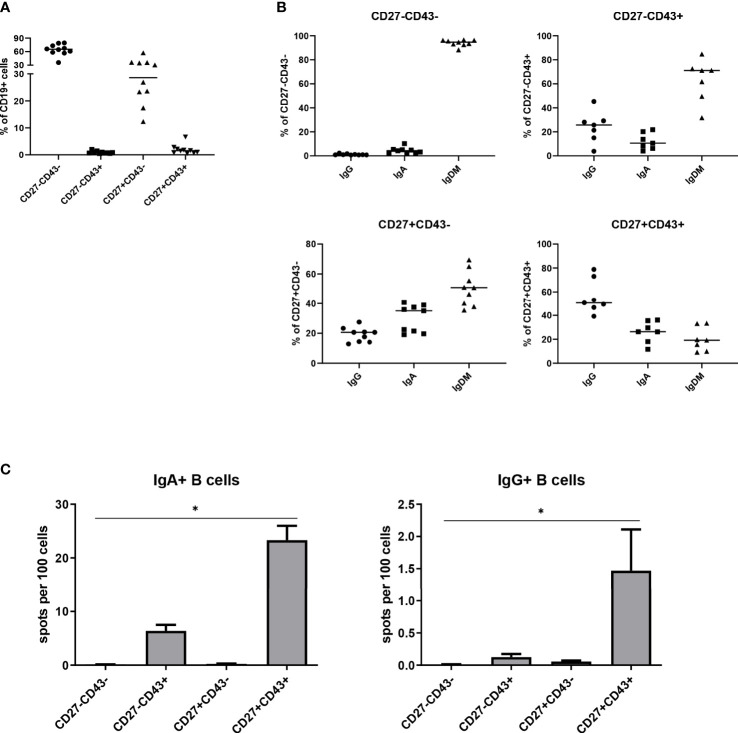
CD3-CD19+CD27-CD43+ B cells are present in peripheral blood. **(A)** Mean percentages of the different B cell subpopulations (CD27-CD43-, CD27-CD43+, CD27+CD43- and CD27+CD43+), determined as a percentage of CD3-CD19+ B cells, in peripheral blood of ten healthy adults. **(B)** Obtained B cell subpopulations were further analyzed for their expression of IgA, IgG, and IgMD. IgMD expression groups cells that are single positive for IgM or IgD, or are double positive for IgM and IgD. Subpopulations for which the sum of the IgA, IgG, and IgMD did not equal 100% ± 7% were excluded.Cells were isolated from a single donor. **(C)** CD3-CD19+IgM-IgD-IgA+ B cells were sorted according to CD27 and CD43 expression. Spontaneous IgA (left panel) and IgG (right panel) antibody secretion by the different B cell subpopulations [CD27-CD43-, CD27-CD43+, CD27+CD43- and CD27+CD43+], analyzed by ELISPOT. Cells were isolated from three different donors. The data were pooled. Bars indicate mean ± SEM. Statistical comparison between groups was done by Kruskal-Wallis test followed by a Dunns multiple comparison test. *p<0.05, **p<0.01, ***p<0.001 compared to CD27-CD43-.

In previous work (25), we observed the presence of a small population of CD43+ cells within the CD19+CD27- B cell fraction in the peripheral blood of healthy subjects. We here analyzed more thoroughly the frequency of the CD3-CD19+CD27-CD43+ and other B cell subpopulations in the peripheral blood of nine healthy adults. Within peripheral blood CD3-CD19+ B cells, CD27-CD43+, CD27+CD43+, CD27-CD43- and CD27+CD43- cells amounted to, respectively, 0.9 ± 0.5%, 1.9 ± 1.8%, 64.0 ± 12.6% and 29.8 ± 12.5% (n=10) (**Figure 1A**). The isotype distribution within each subpopulation is given in **Figure 1B**. On average 12.1 ± 6.3% (standard deviation) of the CD27-CD43+ cells expressed IgA and 24.1 ± 11.9% expressed IgG, whereas 52.8 ± 14.6% of the CD27+CD43+ cells expressed IgA and 20.0 ± 9.17% expressed IgG. (**Figure 1B**). We further characterized IgM-IgD- class-switched CD27-CD43+ B cells, thereby excluding transitional and marginal zone B lymphocytes (for gating strategy, see **Figure S1**).

The capacity of class-switched CD27-CD43+ B cells to spontaneously secrete IgA and IgG antibodies *ex vivo* was studied by ELISPOT. CD27-CD43+ B cells spontaneously secreted IgA and IgG, albeit less than CD27+CD43+ B cells (**Figure 1C**). CD43- class-switched B cells (irrespective of CD27 expression) did not secrete IgA or IgG antibodies (**Figure 1C**). Thus, CD27+CD43+ and CD27-CD43+ cells are ASC, whereas class switched CD27+CD43- and CD27-CD43- cells are Bmem.

### Isotype subclass distribution of CD27-CD43+ ASC

**Figure 2:**
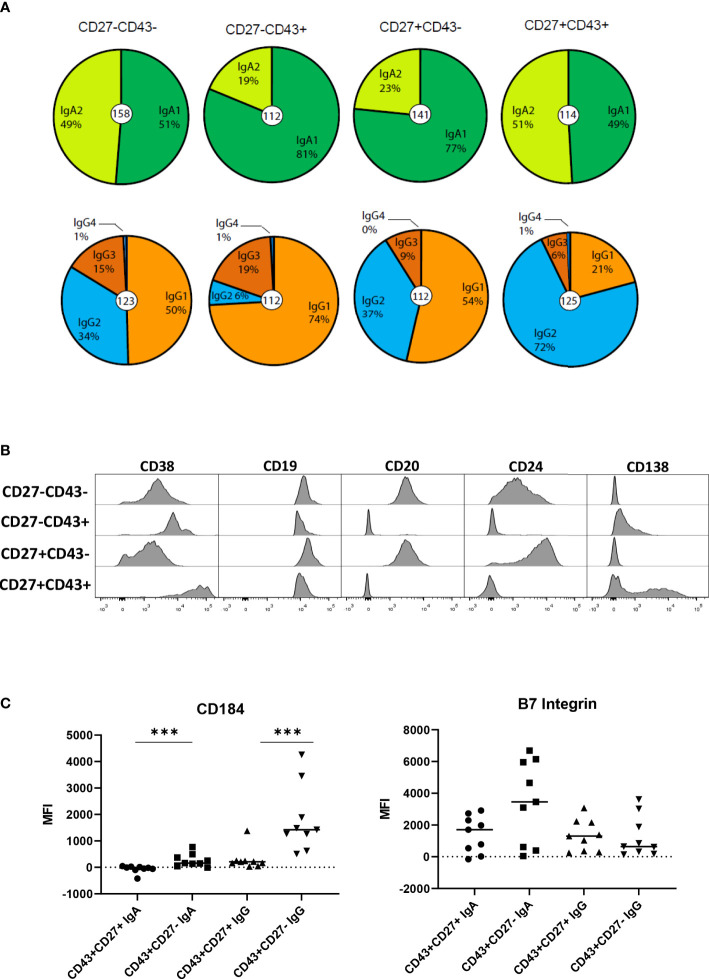
IgA and IgG receptor subclass distribution and phenotype of the different B cell populations. **(A)** The IgA and IgG receptor subclass distribution by sequence analysis for the different cell populations (n= 158, 112, 141, and 114 for IgA analysis and 123, 112, 112, 125 for IgG analysis in respectively CD27-CD43-, CD27-CD43+, CD27+CD43- and CD27+CD43+ (IgM- IgD-) B cells). Data are pooled data from 3 independent donors. **(B)** B Cells were stained with different antibodies and analyzed by flow cytometry. Plots shown are from a representative experiment of 3 healthy donors. **(C)** MFI (mean fluorescence intensity) for CD184 (CXCR4) and B7 integrin on CD27-CD43-, CD27-CD43+, CD27+CD43- and CD27+CD43+ B cells for 9 healthy individuals. Statistical comparison between CD43+CD27+ and CD43+CD27- cells was done by Mann Whitney U test. *p<0.05, **p<0.01, ***p<0.001.

The IgA and IgG receptor subclass distribution of the CD27+ and CD27- ASC and Bmem was assessed by sequence analysis of rearranged *IGH* transcripts in 3 independent donors (**Figure 2A**). IgA1 was the dominant IgA subclass in CD27- ASC and CD27+ Bmem, whereas IgA1 and IgA2 were equally distributed in CD27+ ASC and CD27- B mem. IgG1 was the dominant IgG subclass in CD27- ASC (74%), whereas IgG2 was the dominant subclass in CD27+ ASC (72%). Bmem populations differed slightly in IgG subclass distribution, with more IgG3 in the CD27- subset.

### Surface phenotype of CD27-ASC

Since high expression of CD38 is a feature of conventional ASC (21), we analyzed the expression of CD38 on the CD43+ B cells by flow cytometry. CD38 was expressed on CD27- ASC, albeit at lower levels (mainly on IgG+ cells) than on CD27+ ASC (**Figure 2B**). Expression levels of B cell differentiation markers CD19, CD20, and CD24 are known to decrease upon differentiation to CD27+ ASC (10, 21). Expression of CD20 and CD24 was lower on CD27- and CD27+CD43+ ASC than on CD43- cells (**Figure 2B**). A large fraction of CD27+ ASC expressed CD138. A small fraction of CD27- ASC expressed CD138, although at lower levels than on CD27+ ASC. The expression of CD20, CD24, and CD38 on IgA and IgG ASC is shown in **Figure S2**.

IgG and IgA CD27- ASC expressed significantly higher levels of the homing receptor CD184 (CXCR4) (homing to bone marrow, lymph node, Peyer patch) (2) than CD27+ ASC (**Figure 2C**). IgA+ CD27- ASC expressed higher levels of β7-integrin (homing to gut) (2) than CD27+ASC, albeit not statistically significantly different.

Taken together, CD27-ASC have phenotypical characteristics reminiscent of conventional CD27+ ASC. For homing receptor CXCR4, differences were observed between ASC.

### Gene expression profiling

To compare the CD27- ASC population with the CD27+ ASC and with Bmem, gene expression analysis was performed. RNA was isolated from sort-purified IgA and IgG-expressing B cell populations obtained from 3 healthy adults, and microarray analysis was performed.

**Figure 3:**
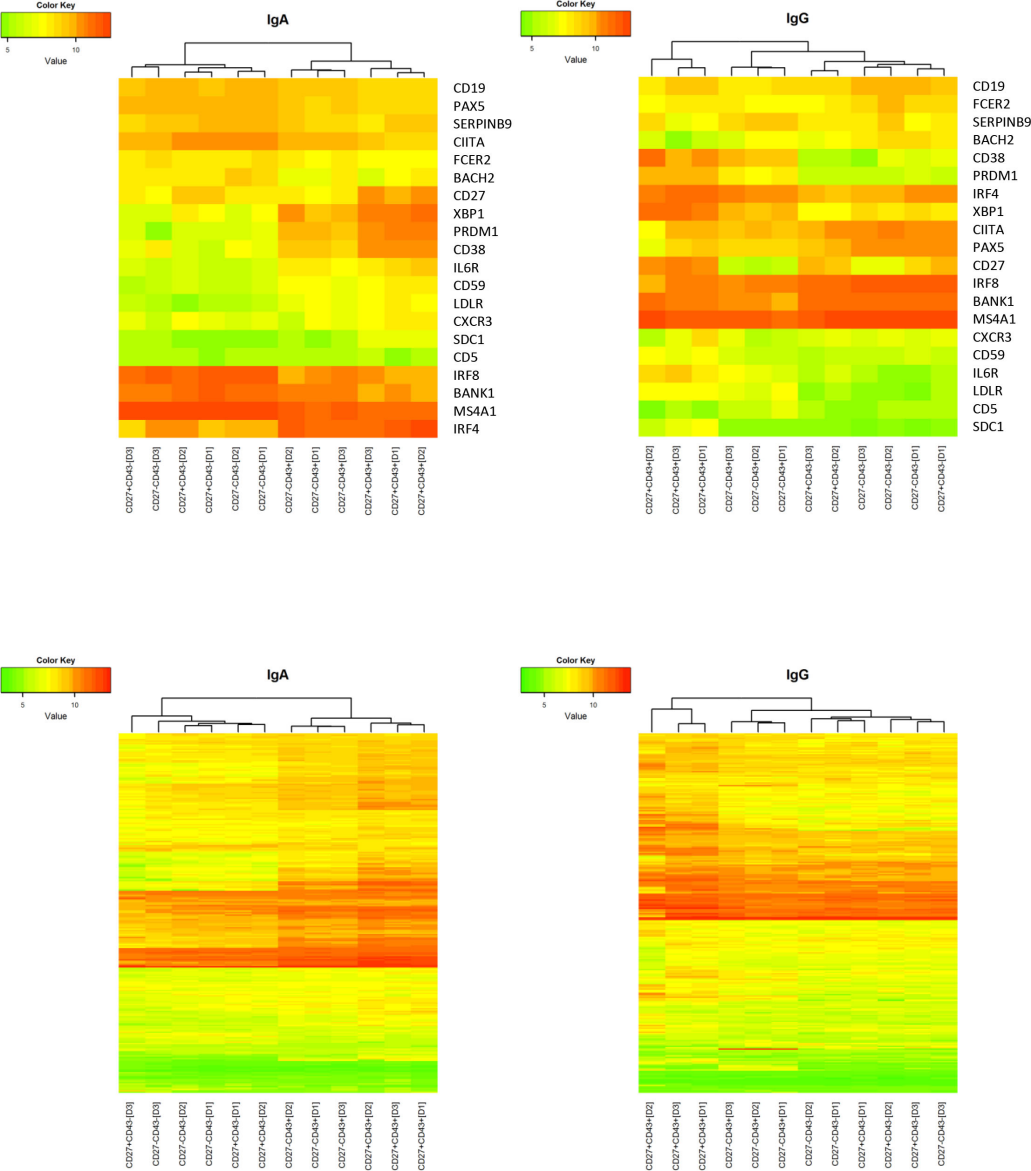
Gene expression profile of CD27-CD43-, CD27-CD43+, CD27+CD43- and CD27+CD43+ B cells. The gene expression profile of different (IgM- IgD-) B-cell subsets was determined on a selection of typical plasma cell marker genes and clustering analysis on log2-transformed normalized expression value was performed (green/red scale bar) for IgA+ (left panel) and IgG+ (right panel) cells. If a gene was represented by more than one probe set the average expression value over the different probe sets was used as the expression value of the gene. The scale ranges from 3.5 to 13.1. The genes included 19 established human markers of late B cell differentiation (upper two panels) and 253 human homologs of murine ASC-related genes (lower two panels) (34, 35). The genes are shown in **Table S2**. Data are from a single experiment including three different donors (D1, D2, D3). Normalization of the transcriptome data is described in Material and Methods.

Hierarchical clustering expression analysis was performed on the expression level (i) of 19 established human markers of late B cell differentiation (34) and (ii) of 253 human homologues of murine ASC-related genes (35) (**Table S2**). Within IgA-expressing subsets, CD27- ASC clustered with CD27+ ASC, separate from CD27- and CD27+ Bmem (**Figure 3**). For many genes, the expression levels in CD27- ASC were intermediate between CD27+ ASC and CD27+ Bmem. Within IgG-expressing subsets, CD27- ASC clustered closer to Bmem than to CD27+ ASC (for both sets of genes analyzed) (**Figure 3**).

In CD27- as well as in CD27+ ASC, an increase in CD38 and PRDM1 (Blimp-1) and a decrease in MS4A1 (CD20) and PAX5 (a repressor of B cell differentiation) was noted, which is consistent with late-stage differentiating B cells (34) (**Figures 3**, **S3**).

Next, we studied the expression levels of (i) Toll-like receptors, (ii) chemokines and their receptors, and (iii) interleukins and their receptors on CD27- and CD27+ ASC. The data are summarized in **Table S3** and show no statistically significant difference (p<0.05 after correction for multiple comparisons: see Materials and Methods for description of statistical analysis) in the expression of these genes for the two analyzed types of IgA ASC. However, IgG secreting CD27- ASC had a significantly higher expression (with all probe sets) of TLR2, TLR8, CCL4, CCL5, CCR3, CXCR2 (CXCR4, albeit not for all probe sets), CXCL2, CX3CR1, IL18RAP, IL21R, IL32, and IL1RN.

Principal component analysis of gene expression of all genes provided on the microarray revealed that gene expression in CD27-CD43+ ASC was distinct from the gene expression in CD27+CD43+ ASC and in CD27-CD43- and CD27+CD43- Bmem (**Figure S4**). Heat maps and principal component analysis of highly statistically significant differentially expressed genes [between CD27+CD43+ ASC, CD27+CD43- Bmem, CD27-CD43+ ASC, and CD27-CD43- Bmem] are shown in **Figure S5**. CD27-CD43+ ASC shares more transcripts in common with CD27+CD43+ ASC than with Bmem. Genes linked to cell cycle/cell division, endoplasmic reticulum, unfolded protein binding, Golgi apparatus, and ribosome (biogenesis) had a higher expression in CD27-CD43+ and CD27+CD43+ ASC than in CD27-CD43- and CD27+CD43- Bmem.

### Replication history and SHM levels in CD27- ASC

**Figure 4:**
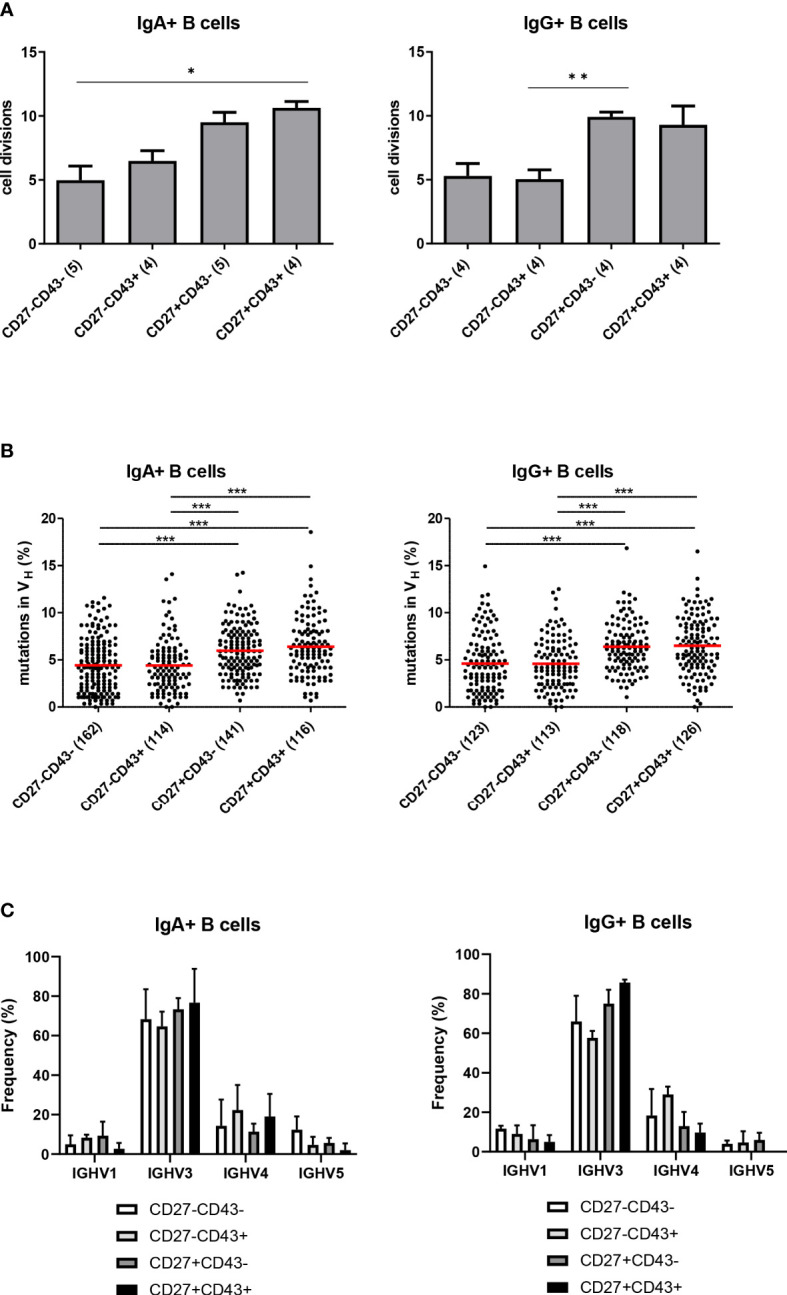
Replication history, SHM levels and IGHV family usage of the different B cell subsets. **(A)** The replication history of purified populations CD27+CD43-, CD27+CD43+, CD27-CD43- and CD27-CD43+ (of CD3-CD19+IgM-IgD-IgG+ B cell subpopulations) of both the IgA and IgG isotype as measured with the KREC assay. Bars represent mean values with SEM from the replication histories determined from cells isolated from 3 different donors (pooled data). Statistical analysis was done by Kruskal-Wallis test followed by a Dunns multiple comparison test *p<0.05; **p<0.01; ***p<0.001. **(B)** Frequency of mutated nucleotides in rearranged IGHV genes from cells isolated from 3 independent donors (pooled results shown). All individual data points are shown as black dots, with red lines indicating the mean value. Statistical analysis was done by Kruskal-Wallis test followed by a Dunns multiple comparison test *p<0.05; **p<0.01; ***p<0.001. **(C)** The relative frequencies of IGHV1, IGHV3, IGHV4, and IGHV5 usage in the different CD3-CD19+IgM-IgD-IgA+ B cell subpopulations (Left panel) and in the CD3-CD19+IgM-IgD-IgG+ B cell subpopulations (right panel). Cells were isolated from three different donors. Data represent mean ± SD. In IgG (but not IgA) expressing CD27- ASC, the frequency of IGHV3 usage was lower, and the frequency of IGHV4 usage was higher, than in CD27+ ASC (p<0.001) (χ2 test).

Purified populations of CD27+ and CD27- ASC and Bmem of both the IgA and IgG isotype from 3 healthy subjects were analyzed for their *in vivo* replication history and frequency of somatic mutations in their immunoglobulin transcripts. The replication histories of IgG and IgA CD27- ASC and Bmem populations ( ± 5 divisions) were lower than the replication history of IgG and IgA CD27+ B cell populations ( ± 10 divisions) (**Figure 4A**).

The frequency of SHM was similar between CD27+ ASC and CD27+ Bmem and significantly higher than in CD27- ASC and CD27- Bmem (**Figure 4B**). Thus, CD27- Bmem and CD27- ASC showed less extensive signs of molecular maturation than their CD27+ counterparts.

### Ig repertoire selection in CD27- ASC

IGH repertoire analysis of sorted cells revealed that in IgG (but not IgA) expressing CD27- ASC the frequency of IGHV3 usage was lower, and the frequency of IGHV4 usage was higher, than in CD27+ ASC (p<0.001) (**Figure 4C**).

Because the CDR3 region of the Ig gene is considered to impact antigen specificity, we also studied CDR3 characteristics of the IGH repertoire in the different cell populations. Sizes of CDR3 differed (p=0.02: Fisher’s LSD test) between IgG CD27-CD43+ cells [mean-SD: 15.2-3.6, n=113; data are pooled from 3 different donors)] and IgG CD27+CD43+ cells [mean-SD: 14.1-3.7 n=126; 3 donors)]. No such differences were observed for IgA [mean, SD: 14.6, 3.6 (n=114 from 3 different donors) for CD27-CD43+ and 14.2, 3.8 (n=116 from 3 different donors) for CD27+CD43+ (p=0.39: Fisher’s LSD test)]. The hydrophobicity index (Kyte en Doolittle) was not significantly different between the CD27-CD43+ and CD27+CD43+ populations (p=0.18 for IgG ASC and 0.45 for IgA ASC; Fisher’s LSD test).

In addition, selection for replacement mutations in the rearranged IGHV genes was performed using the BASELINe program (**Supplemental data Figure S6**). A selection strength >0 represents an increased replacement/silent mutation ratio over what can be expected based on random chance. The selection strength for framework regions (FR) of all four subsets was <0, fitting with selection against replacement mutations. Still, this selection against replacement mutations was significantly less for the CD27- Bmem than the other three subsets. There were no differences in the selection of complementarity-determining regions (CDR) in IgA transcripts. Similar to IgA, the IgG FR of CD27+ ASC and CD27+ Bmem had significantly fewer replacement mutations than CD27- ASC (p<0.05). In addition, the IgG CDR of CD27+ Bmem had significantly more replacement mutations than CD27- Bmem and CD27- ASC (p<0.05). Thus, the higher levels of SHM in CD27+ Bmem and ASC were associated with increased selection for replacement mutation in CDR as compared to CD27- Bmem and ASC.

### Antibody signature by protein microarray

In order to study the antibody signature (antibody repertoire) we evaluated immune reactivity to 7390 human proteins by protein microarray. CD27- and CD27+ ASC and CD43+ and CD43- Bmem from 3 different healthy donors were stimulated *in vitro* with CpG (see Material and Methods) and the supernatants were concentrated and used for protein microarray-based profiling. As an insufficient number of CD43+CD27+ and CD43-C27- cells were available, the antibody signature of these cells could not be studied (see Materials and Methods). There were 1599 proteins to which there was a significant difference in reactivity between CD43- Bmem and CD43+CD27- ASC cells at the nominal 0.05 level of the univariate test. **Figure S7** shows a heat map of the proteins to which there was differential reactivity and **Table S4**. summarizes the most significant differentially reactive antigens, Uniprot accession numbers, fold changes, p-values, and False Discovery Rates (FDR). These data suggest that the antibody profile of the CD43+CD27- ASC cell population is distinct from the antibody profile of CD43-CD27+ Bmem cells.

### Involvement in vaccine responses

Next, we investigated whether CD27- ASC are involved in the antibody response to vaccination. Rabipur^®^ (inactivated Rabies virus) was used as a model vaccine to induce a primary antibody response as rabies infections and vaccinations in Belgium are extremely rare (and thus the Belgian population is assumed to be naïve for the rabies antigen). Pneumo23/Pneumovax was used as a model polysaccharide vaccine, and Tedivax pro-Adulto^®^, containing tetanus-toxoid, as a model vaccine to induce a secondary antibody response. Healthy young adults were vaccinated with either one of the three vaccines and the presence of CD27- and CD27+ vaccine-specific ASC was analyzed by ELISPOT 7-10 days post-vaccination, a time window for which it is known that antigen-specific ASC can be found in circulation (3, 6, 14). For vaccination with unconjugated pneumococcal vaccine, ASC to the mixture of 23 serotypes, as well as the specific antibody response to serotype 1 and serotype 4 was measured. The results are shown in **Tables 1**, **S5** (serotypes 1 and 4).

**Table 1:** IgA and IgG secretion by different B cell populations after vaccination.

	IgA ASC (spots/100000 cells)				IgG ASC (spots/100000 cells)			
	CD27+ CD43-	CD27+ CD43+	CD27- CD43-	CD27- CD43+	CD27+ CD43-	CD27+ CD43+	CD27- CD43-	CD27- CD43+
Rabipur								
HV1 7d	0	23	0	1	0	14	0	0
HV2 7d	0	391	0	0	0	6	0	1
HV3 10d	0	55	0	2	0	80	0	3
HV4 10d	0	119	0	2	0	122	0	2
HV5 10d	0	107	0	0	0	110	0	0
HV6 10d	0	12	0	0	0	24	0	0
PPV – 23								
HV7 7d*	5	4000	5	1173	0	960	10	723
HV8 7d*	3	10240	0	141	0	3048	0	90
HV9 7d	0	4800	0	15	0	1813	3	10
HV11 10d	3	4267	3	5	2	3200	0	1
HV12 10d	0	3627	0	23	0	2027	0	23
Tedivax								
HV13 7d					21	3905	0	79
HV14 7d					22	7984	0	20
HV15 7d					31	1157	26	41

Fifteen healthy volunteers (HV1-15) (students) were vaccinated with either Rabipur^®^, polysaccharide pneumococcal vaccine (PPV), or Tedivax pro-Adulto^®^. For PPV, Pneumo23 (indicated with *) or Pneumovax was used. The presence of vaccine-specific ASC was analyzed by ELISPOT 7-10 days post-vaccination (as indicated). After vaccination with PPV, ASC was determined (i) for the mix of the 23 polysaccharides contained in PPV (PPV-23), (ii) for polysaccharide serotype 1 (PS-1), and (iii) for polysaccharide serotype 4 (PS-4). Results for PS-1 and PS-4 are documented in the supplementary information (**Table S2**). We observed differences between Pneumo23 and Pneumovax with respect to the induction of CD27- ASC. The reason for this difference is unclear. Both vaccines are produced by the same manufacturer applying similar procedures, albeit at different locations (factories).

All vaccinations induced IgA and IgG vaccine-specific ASC. Rabipur induced lower numbers of ASC than the unconjugated pneumococcal vaccine or Tedivax. For all vaccines studied, the highest frequencies of IgA and IgG vaccine-specific ASC were found in the CD27+ ASC population. Only a few IgA and IgG vaccine-specific ASC were induced within the CD27- ASC population after Rabipur and Tedivax vaccination. Higher levels of vaccine-specific antibodies were observed within the CD27- ASC population post-Pneumo23 vaccination, although much lower than within the CD27+ ASC population.

### CD27- ASC in immune-mediated disorders

**Figure 5:**
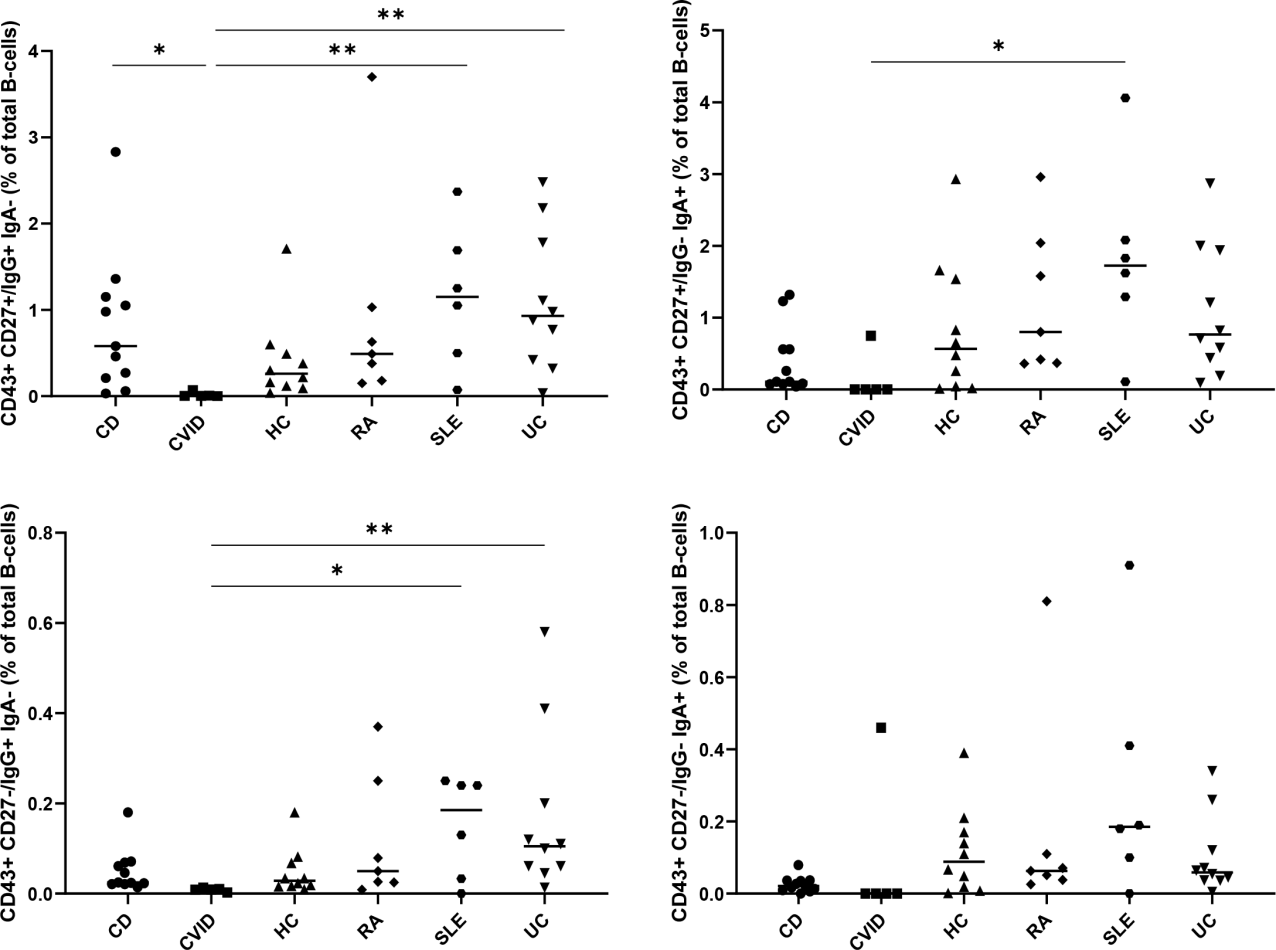
Presence of CD27+ and CD27- ASC in common variable immunodeficiency (CVID), systemic lupus erythematosus (SLE), rheumatoid arthritis (RA), ulcerative colitis (UC) and Crohn’s disease (CD). Percentages of the CD27+CD43+ (upper panels) and CD27-CD43+ (lower panels) IgA+ (right panels) and IgG+ (left panels) B cell subpopulations, determined as percentage of CD3-CD19+ B cells, in peripheral blood of healthy adults (HC) (n=10), of patients with active ulcerative colitis (UC) (n=10), active Crohn’s disease (CD) (n=11), common variable immunodeficiency (CVID) (n=5), active systemic lupus erythematosus (SLE) (n=6), and active rheumatoid arthritis (n=8). All individual data points are shown as black dots, with lines indicating the mean value. Statistical analysis was done by Kruskal-Wallis test followed by a Dunns multiple comparison test *p<0.05; **p<0.01; ***p<0.001. Statistical analysis by univariate non-parametric Kruskal-Wallis revealed that IgG CD27+ and CD27- ASC were significantly higher in UC than in HC, significantly lower (p<0.005: Kruskal-Wallis) in CVID than in HC (p<0.005) and tended to be higher in SLE than in HC (p=0.08 for CD27+ and p=0.12 for CD27-). IgA CD27+ and CD27- were lower in CVID than in HC (p=0.02 for CD27+ and 0.06 for CD27-).

To study the involvement of ASC in chronic inflammation, autoimmunity, and immunodeficiency, we analyzed the frequencies of CD27- and CD27+ ASC in healthy individuals (HC) (n=10), patients with common variable immunodeficiency (CVID) (n=5) and in patients with active ulcerative colitis (UC) (n=10), active Crohn’s disease (CD) (n=11), active rheumatoid arthritis (RA) (n=7) and active systemic lupus erythematosus (SLE) (n=6) (**Figure 5**). Differences in ASC between the different diseases were observed. The lowest levels of IgG+CD27+CD43+ and IgG+CD27-CD43+ ASC were found in CVID, whereas the highest levels were found in UC and SLE. The difference between CVID and UC was statistically significant by non-parametric statistics after correction for multiple comparisons. Without correction for multiple comparisons, IgG CD27+ and CD27- ASC were significantly higher in UC than in HC (p<0.03: Kruskal-Wallis), significantly lower (p<0.005: Kruskal-Wallis) in CVID than in HC (p<0.005) and tended to be higher in SLE than in HC (p=0.08 for CD27+ and p=0.12 for CD27-). IgA CD27+ and CD27- were lower in CVID than in HC (p=0.02 for CD27+ and 0.06 for CD27-).

### Comparison of CD27-CD43+ ASC to double negative (CD27 and IgD) B cells

B cells that are double negative for CD27 and IgD, express CD11c, and are negative for CXCR5 (DN_2_ cells) have been described to be autoreactive and expanded in lupus (39–41). Jens et al. reported that these cells expressed a T-bet transcriptional network with increased expression of TLR7 and lack of TRAF5 (39). Wang et al. reported that these cells express high levels of FcRL5 (40). As these cells have been described to derive from naïve cells and are able to rapidly differentiate into plasmablasts after stimulation (39, 40) we evaluated whether these cells show similarities to the CD27-CD43+ cells described here. ITGAX (CD11c) gene expression was higher in CD27-CD43+ cells than in CD27+CD43+ plasmablasts (**Supplementary Data Table S3**). TBX21 (T-Bet) expression was higher in IgG(+) CD27-CD43+ cells than in IgG(+) CD27+CD43+ plasmablasts [no difference in IgA(+) cells]. TLR7 expression was lower in IgG(+) CD27-CD43+ cells compared to IgG(+) CD27+CD43+ cells [no difference in IgA(+) cells] and TRAF5 expression was higher in IgA(+) CD27-CD43+ cells than in CD27+CD43+ cells [no difference in IgG(+) cells]. There was no difference in FCRL5 expression between CD27-CD43+ and CD27+CD43+ cells.

**Figure 6:**
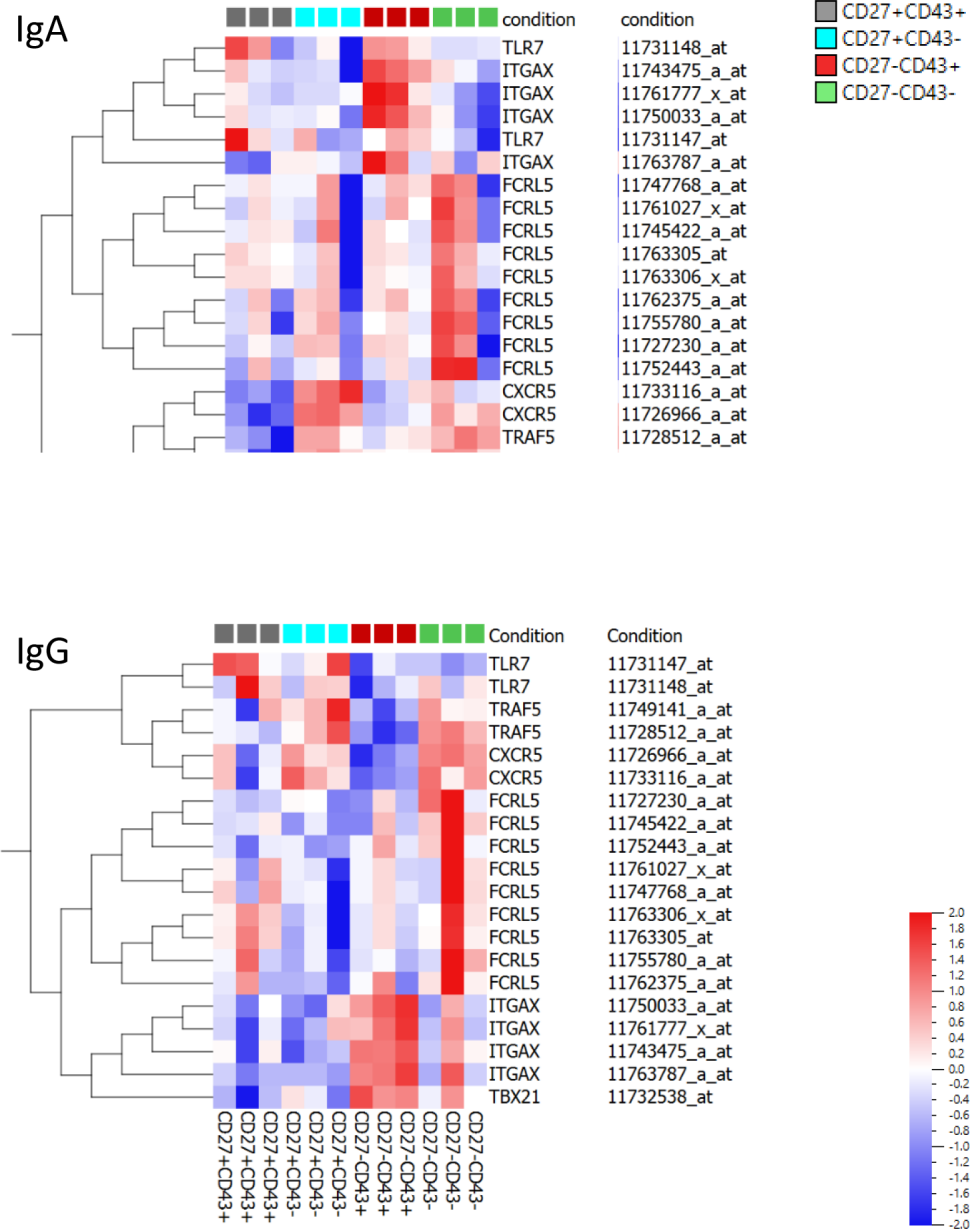
Gene expression profile of ITGAX, TBX21, CXCR5, TLR7, TRAF5, FCRL5. Heat maps of gene expression of ITGAX, TBX21, CXCR5, TLR7, TRAF5, FCRL5 in IgA(+) and IgG(+) CD27+CD43+ and CD27-CD43+ ASC and in CD27+CD43- and CD27-CD43- Bmem. Probe numbers are indicated. The heat map indicates the variance (from -2 to 2) compared to the mean (0).

Heat maps of the gene expression of the above-mentioned genes in CD27-CD43-, CD27-CD43+, CD27+CD43- and CD27+CD43+ cells are shown in **Figure 6**. ITGAX (CD11c) expression was highest in CD27-CD43+ (IgA and IgG expressing) cells. TBX21 expression was highest in IgG(+), but not IgA(+), CD27-CD43+ cells. CXCR5 and TRAF5 expression was lower in CD27-CD43+ cells than in naïve and memory B cells.

Given the high gene expression of ITGAX in CD27-CD43+ cells, we next investigated the likeness of CD27-CD43+ cells to CD11c^hi^ cells described by Maul et al. (41). In particular, we evaluated whether the immune-related transcripts that define CD11c^hi^ cells, as reported by Maul et al. (41) in SLE, also characterize CD27-CD43+ cells. **Figure S8** shows the heat maps of the gene expression of genes that define CD11c^hi^ cells and that significantly (p=0.05) discriminate CD27-CD43+ cells from CD27-CD43-, CD27+CD43- and CD27+CD43+ cells by multigroup comparison (ANOVA). Some genes have a similar expression profile in CD27-CD43+ and CD11c^hi^ cells (e.g. CD27, CXCR5, PRDM1), but others do not (e.g. CD38, IL6R).

We also evaluated CD11c expression by flow cytometry in three healthy donors. For IgA-expressing cells, the number of events in the CD27-CD43+ population was too low (<30 events in 2/3 cases) to allow reliable conclusions. In IgG-expressing cells, the highest expression of CD11c was found in CD27-CD43+ cells. The results of a representative case are shown in **Figure S9**.

## Discussion

In this study, we functionally, morphologically, and phenotypically characterized a small novel population of circulating CD43+ cells within the CD19+CD27- B cell fraction of healthy subjects. We demonstrated that CD27-CD43+ B cells possess many characteristics of conventional plasmablasts, although differences were observed in subclass distribution, replication history, frequency of SHM, expression of homing receptors, TLRs, cytokines, and their receptors. We assume these differences can be attributed to different maturation pathways and origins of CD27-CD43+ ASC and conventional plasmablasts.

Several studies demonstrated the existence of class-switched CD27- memory B cells in peripheral blood, which has raised questions about the usage of this marker to separate memory from naive B cells (22, 29, 42). Memory cells that are double negative for IgD and CD27 represent approximately 5% of all B cells in healthy adults (43). To the best of our knowledge, we show here for the first time the existence of an antibody-secreting population within the class-switched CD27- B cell fraction of healthy individuals. Spontaneous secretion was mainly restricted to the IgA isotype and the fraction of ASC was lower than the fraction of ASC for conventional CD27+CD43+ plasmablasts. These observations suggest that CD27-CD43+ B cells might be activated CD27-memory cells on their way to plasmablast/plasma cell differentiation.

Besides spontaneous antibody secretion, we analyzed the involvement of circulating ASC subsets against different types of vaccines by ELISPOT analysis. We found that CD27+ ASC were involved in primary responses and in recall responses against protein and polysaccharide antigens, whereas CD27- ASC were not involved in such responses or to a much lesser extent (and mainly restricted to polysaccharide antigens).

Plasmablasts are characterized by high surface expression levels of CD38. The newly identified CD27- ASC express CD38, however, the expression level is lower than the expression level on conventional plasmablasts. A small percentage of CD27-CD43+ B cells expressed low levels of the plasma cell marker CD138, albeit slightly lower than in CD27+CD43+ B cells. These observations further support the hypothesis that CD27- ASC are in an early state of differentiation towards plasmablasts/plasma cells. Surface expression of CXCR4 was significantly higher on IgG+CD27- ASCs than on IgG+CD27+ASC. CXCR4 is involved in B-cell recruitment into intestinal lymphoid tissue (Peyer’s patches) and for retention of developing B cells within the bone marrow microenvironment (2, 44, 45). Transcriptome analysis confirmed a higher expression of CXCR4 in IgG CD27- ASC than in CD27+ ASC. A fraction of IgA+ CD27- ASC expressed β7-Integrin, indicating that this sub-fraction of cells traffic to and are retained in the gut epithelial layer (46). Although more thorough analysis is needed (e.g. inclusion of more homing markers), our data clearly indicate differences in the homing potential for different ASC.

Hierarchical clustering of gene expression profiles revealed that the IgA CD27- ASC were most similar to the IgA CD27+ ASC, whereas the IgG CD27- ASC clustered with IgG Bmem. This might indicate that there are differences in differentiation pathways between IgA and IgG ASC. It should be mentioned that in our studies we did not directly compare expression profiles of IgG and IgA subpopulations as the two populations were obtained from different donors.

Gene expression studies revealed additional differences in expression between CD27+ and CD27- ASC. For example, the expression of (i) the chemokine receptors and chemokines CXCR2 and CCL5, (ii) the interleukins and interleukin receptors IL32, IL21R, IL1RN, IL18RAP and (iii) TLR2 was higher in IgG CD27- ASC than in IgG CD27+ ASC. No such differences were found in IgA ASC. This could indicate that the different ASC (CD27+ versus CD27- and IgG versus IgA) might be induced by different stimuli, fulfill a different physiological role and/or have a different origin or fate. B cell signaling through the IL-21 receptor has been reported to be required for establishing long-lived antibody responses in humans (47).

Gene expression profiling revealed that CD27-CD43+ ASC shows similarities to CD11c^hi^ cells and DN_2_ cells in SLE, with increased expression of ITGAX (CD11c) and TBX21 (T-bet) and decreased expression of CD24, CD27, and CXCR5. There are, however, also differences between CD27-CD43+ APC and CD11c^hi^ and DN_2_ cells, such as the higher expression of CD38 in CD27-CD43+ ASC. Further research is needed to better characterize the different cell populations and their functionality in health and disease. Wang et al. reported that CD11c^hi^ B cells from healthy donors do not share a similar transcriptome pattern with the transcriptome pattern from SLE cells for many genes (40).

We found fewer cell divisions and lower frequencies of SHM in CD27- ASC (both IgA and IgG) than in CD27+ ASC, which indicates differences in origin and function. The low frequency of SHM and number of cell divisions (approximately 5 cell cycles) could indicate that the CD27- ASC originate outside germinal centers in the absence of T-cell help. Other studies already suggested that CD27-IgA+ memory B cells emerged independently from T cell help or from CD40-CD40L interactions in humans (24). Alternatively, the low frequency of SHM could also indicate that CD27- ASC originated from B cells following T-cell help, which then underwent extrafollicular differentiation into CD27- ASC (42).

Studies on memory B cells demonstrated already differences in origin and function between CD27- and CD27+ Bmem (22, 24, 29). Berkowska et al. (24, 29) analyzed the replication history, SHM, and class-switch profiles of CD27-IgA+ and CD27-IgG+ Bmem cells. Similar to our results in CD27- ASCs, they observed only a few cell divisions (approximately 4), a low frequency of SHM, and a dominant subclass use of IgA1 (67%) in IgA+CD27- memory B cells. Moreover, in accordance with the low frequency of CD27- ASC in peripheral blood in our study, CD27- Bmem are present in low amounts as well (22). Based on these similarities, we propose that CD27-IgA+ memory cells and CD27-IgA+ ASC are derived from similar immune responses. In contrast, Berkowska et al. (24) observed a higher number of cell divisions (approximately 9) in CD27-IgG+ Bmem, albeit with a low frequency of SHM. In agreement with our findings in IgG+CD27- ASC, Berkowska et al. (24) reported that IgG+CD27- memory cells predominantly use the IgG1 subtype.

Similar to our results, Fecteau et al. (22) described CD27-IgG+ Bmem with a lower mutation frequency than CD27+IgG+ Bmem, while their phenotype and morphology were similar. Wu et al. (42) also showed a decreased level of SHM in CD27-IgD- compared to CD27+IgD- Bmem. Colonna-Romano et al. (48) suggested that CD27- cells are exhausted memory cells that have downregulated CD27. As replication history and SHM were lower in the CD27- ASC than in the CD27+ ASC in our study as well as in other studies (22, 24), we conclude that the CD27- ASC population cannot be defined as exhausted memory cells or exhausted plasmablasts.

We used a high-density protein microarray to study differences in antigen reactivity. Our results indicate that the antibody signature (repertoire) of CD43+CD27- cells are distinct from the antibody signature of CD43-CD27+ cells as we found that the antibodies induced (by CpG stimulation) by both cell populations differentially reacted to antigens present in the microarray. The frequency of conventional plasmablasts can alter in patients suffering from immune-mediated diseases. For example, immature plasma cells are increased in patients with UC, although not consistently found to be different in patients with CD (49, 50). In our study, we found the lowest levels of IgG CD27- and IgG CD27- ASC in CVID and the highest levels in patients with active UC. Our results thus confirm an increased frequency of conventional IgG CD27+CD43+ plasmablasts in UC. Such alterations were also observed for IgG CD27-CD43+ ASC. Moreover, we found that CD27+ and CD27- ASC cluster subgroups of UC, SLE, and RA away from CVID and HC. Our results suggest that ASC are altered in immune-mediated pathology but are exploratory and further research is needed to unravel the role of CD27- ASC in immune disorders.

In conclusion, we demonstrated that class-switched CD27-CD43+ B cells possess characteristics of conventional plasmablasts as they spontaneously secrete antibodies, are morphologically similar to ASC, show downregulation of B cell differentiation markers CD20 and CD24, express CD38, and have a gene expression profile related to plasmablasts. Despite these similarities, we observed (antibody-class-dependent) differences between CD27-CD43+ and CD27+CD43+ ASC in IgA/IgG subclass distribution, replication history, and frequency of SHM (lower in CD27-CD43+), vaccination responses, antibody repertoire, expression of homing markers, TLRs, cytokines and their receptors (higher in IgG+CD27-CD43+). These differences suggest that CD27-CD43+ cells respond to different stimuli, fulfill a different physiological role, and have a different maturation pathway or fate than CD27+ ASC. CD27-CD43+ B cells are likely at an early developmental stage and/or are generated from T-cell independent or extrafollicular responses with differences in function and homing potential. Additional studies are needed to unravel the role of CD27- ASC in health and immune disorders.

## Data availability statement

Human gene expression array data have been deposited in the ArrayExpress database at EMBL-EBI (www.ebi.ac.uk/arrayexpress) under accession number E-MTAB-12406.The protein microarray data discussed in this publication have been deposited in the GEO repository and are accessible through GEO Series accession number GS217855 (https://www.ncbi.nlm.nih.gov/geo/query/acc.cgi?acc=GSE217855).

## Ethics statement

The study was approved by the Ethics Committee of the University Hospitals of Leuven (S52146) (S53684). Informed consent was obtained from participants in the vaccination and immunophenotyping studies and from all patients included in the study.

## Author contributions

KC, BV, MvZ, JvD and XB designed the study. BV, KC and SS drafted the manuscript. KC and XB finalized the manuscript. KC, BV, BD, LM, GW, JC and KN conducted the experiments. KC, BV, GV, BD, MvZ, JC, AW and XB analyzed the results. SV characterized the IBD, RS characterized the CVID patients, PV characterized the RA patients and ED characterized the SLE patients. AW performed the protein microarray analyses. All authors contributed to the article and approved the submitted version.
