# Supplementary material for: Plasma cells are not restricted to the CD27+ phenotype: characterization of CD27-CD43+ antibody-secreting cells
Source: Front Immunol. 2023 Jul 10;14:1165936. doi: 10.3389/fimmu.2023.1165936 (PMC10364057; doi:10.3389/fimmu.2023.1165936)
Supplement: Supplementary file 1 [file Presentation_1.pptx]

## Slide 1
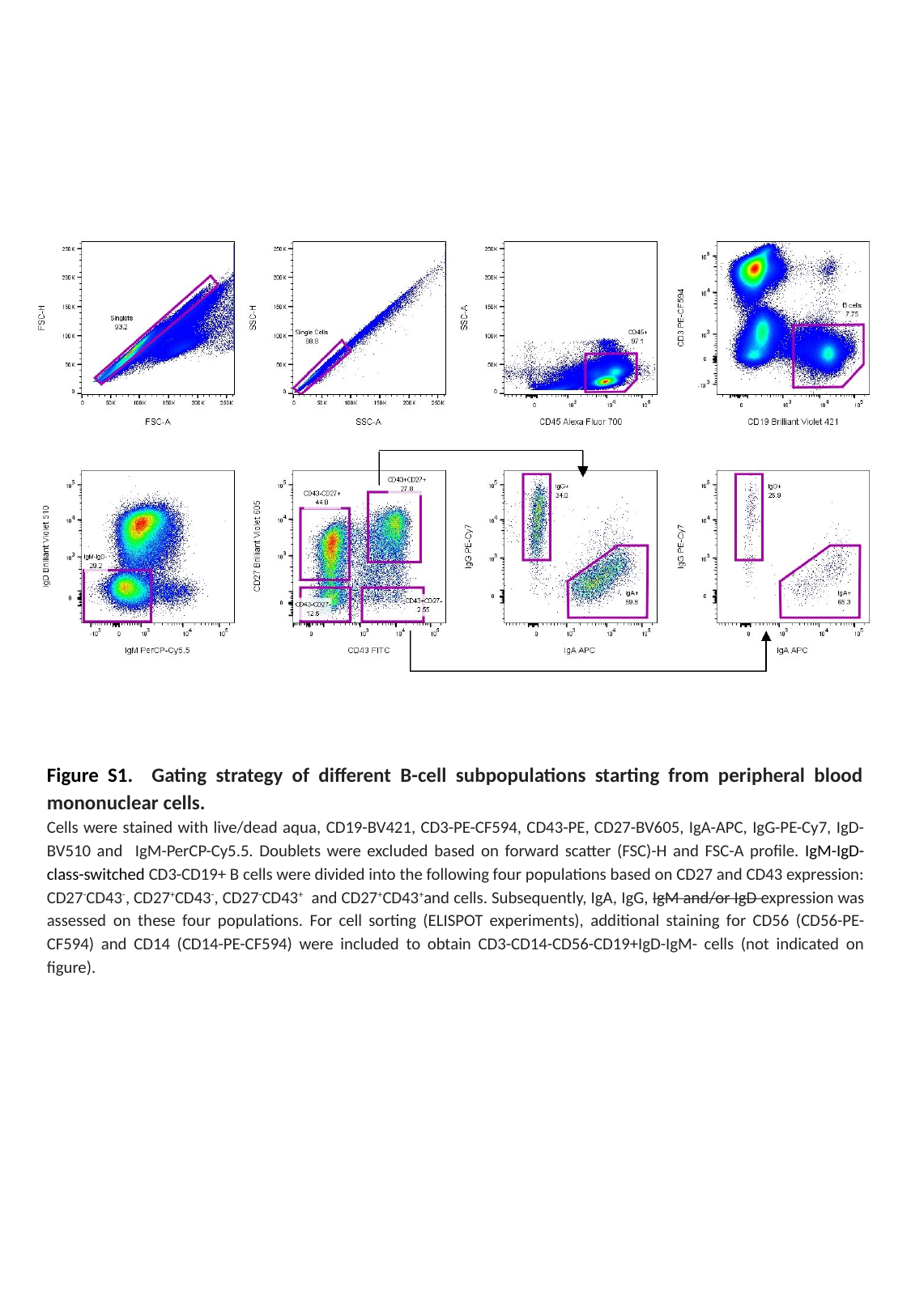

Figure S1. Gating strategy of different B-cell subpopulations starting from peripheral blood mononuclear cells.
Cells were stained with live/dead aqua, CD19-BV421, CD3-PE-CF594, CD43-PE, CD27-BV605, IgA-APC, IgG-PE-Cy7, IgD-BV510 and IgM-PerCP-Cy5.5. Doublets were excluded based on forward scatter (FSC)-H and FSC-A profile. IgM-IgD- class-switched CD3-CD19+ B cells were divided into the following four populations based on CD27 and CD43 expression: CD27-CD43-, CD27+CD43-, CD27-CD43+ and CD27+CD43+and cells. Subsequently, IgA, IgG, IgM and/or IgD expression was assessed on these four populations. For cell sorting (ELISPOT experiments), additional staining for CD56 (CD56-PE-CF594) and CD14 (CD14-PE-CF594) were included to obtain CD3-CD14-CD56-CD19+IgD-IgM- cells (not indicated on figure).

## Slide 2
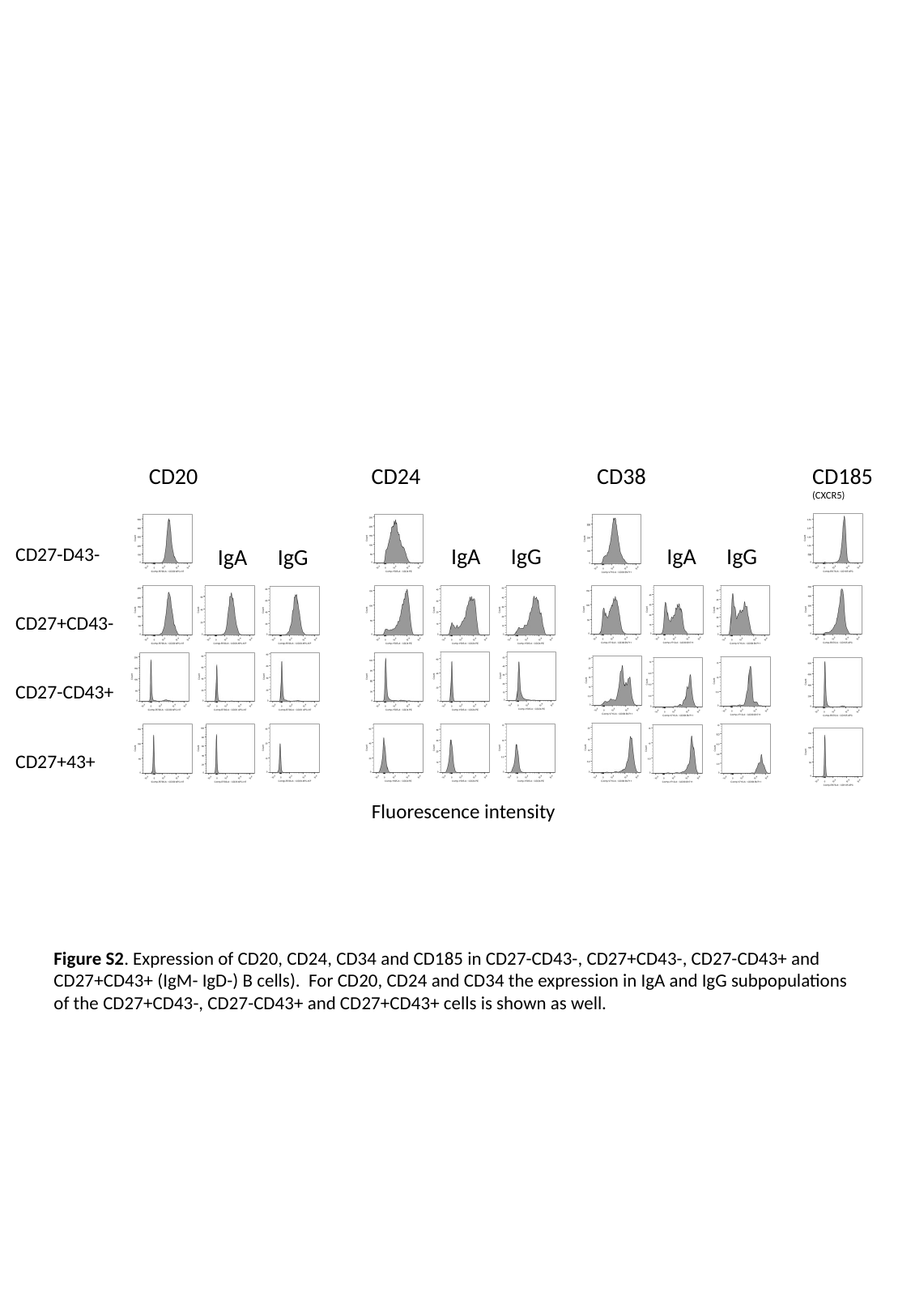

CD38
CD185
(CXCR5)
CD24
CD20
IgA IgG
IgA IgG
CD27-D43-
CD27+CD43-
CD27-CD43+
CD27+43+
IgA IgG
Fluorescence intensity
Figure S2. Expression of CD20, CD24, CD34 and CD185 in CD27-CD43-, CD27+CD43-, CD27-CD43+ and CD27+CD43+ (IgM- IgD-) B cells). For CD20, CD24 and CD34 the expression in IgA and IgG subpopulations of the CD27+CD43-, CD27-CD43+ and CD27+CD43+ cells is shown as well.

## Slide 3
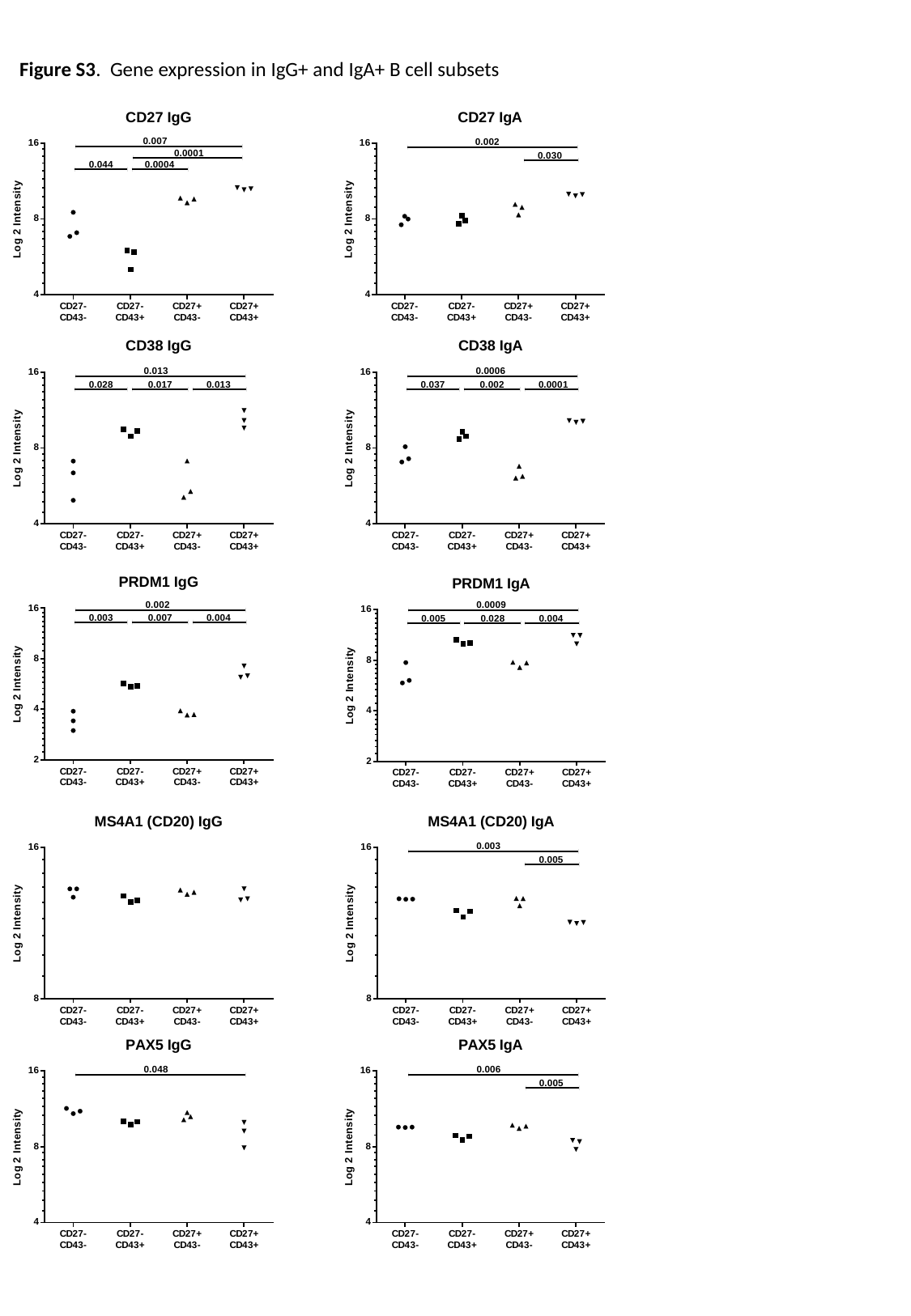

Figure S3. Gene expression in IgG+ and IgA+ B cell subsets

## Slide 4
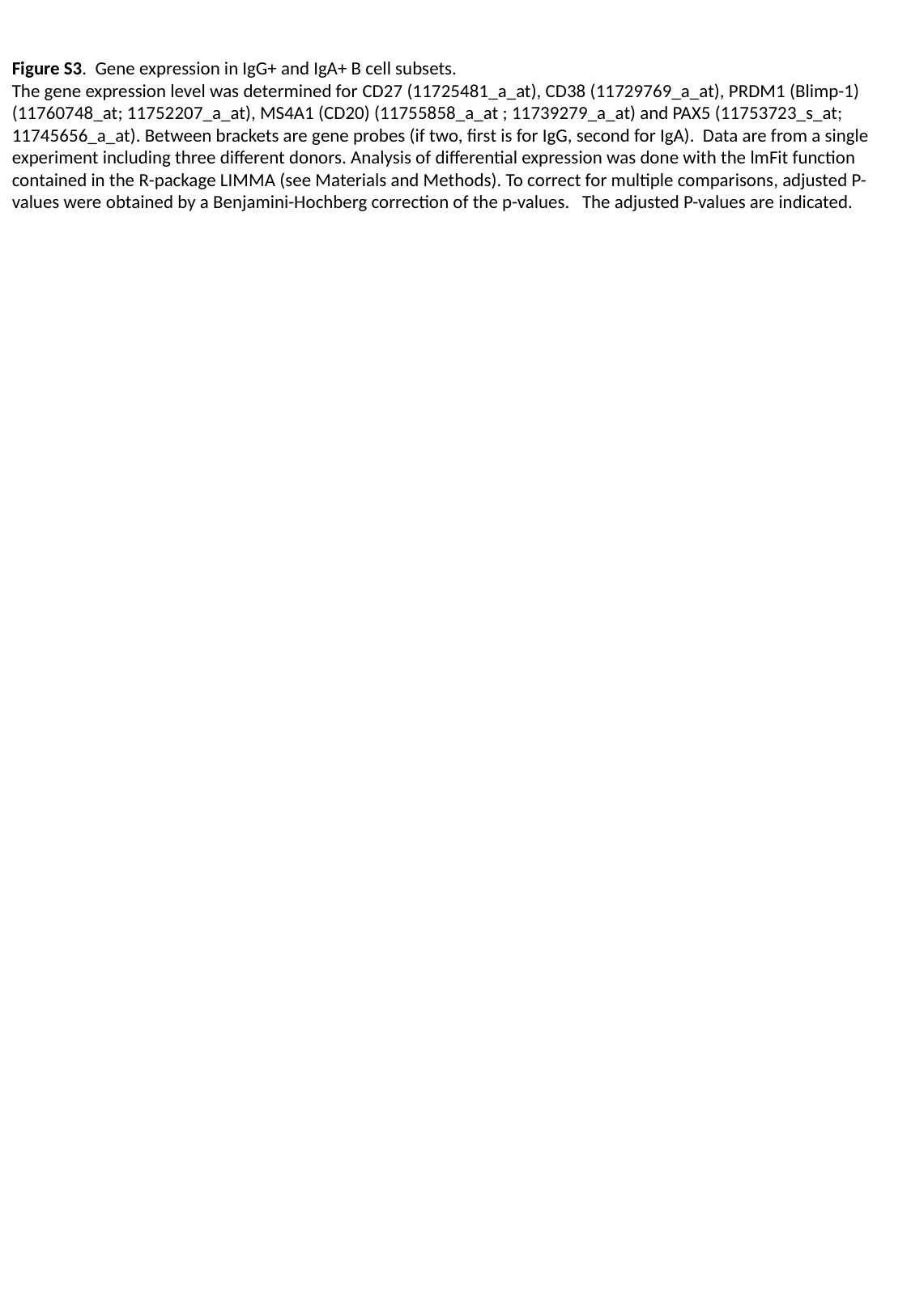

Figure S3. Gene expression in IgG+ and IgA+ B cell subsets.
The gene expression level was determined for CD27 (11725481_a_at), CD38 (11729769_a_at), PRDM1 (Blimp-1) (11760748_at; 11752207_a_at), MS4A1 (CD20) (11755858_a_at ; 11739279_a_at) and PAX5 (11753723_s_at; 11745656_a_at). Between brackets are gene probes (if two, first is for IgG, second for IgA). Data are from a single experiment including three different donors. Analysis of differential expression was done with the lmFit function contained in the R-package LIMMA (see Materials and Methods). To correct for multiple comparisons, adjusted P-values were obtained by a Benjamini-Hochberg correction of the p-values. The adjusted P-values are indicated.

## Slide 5
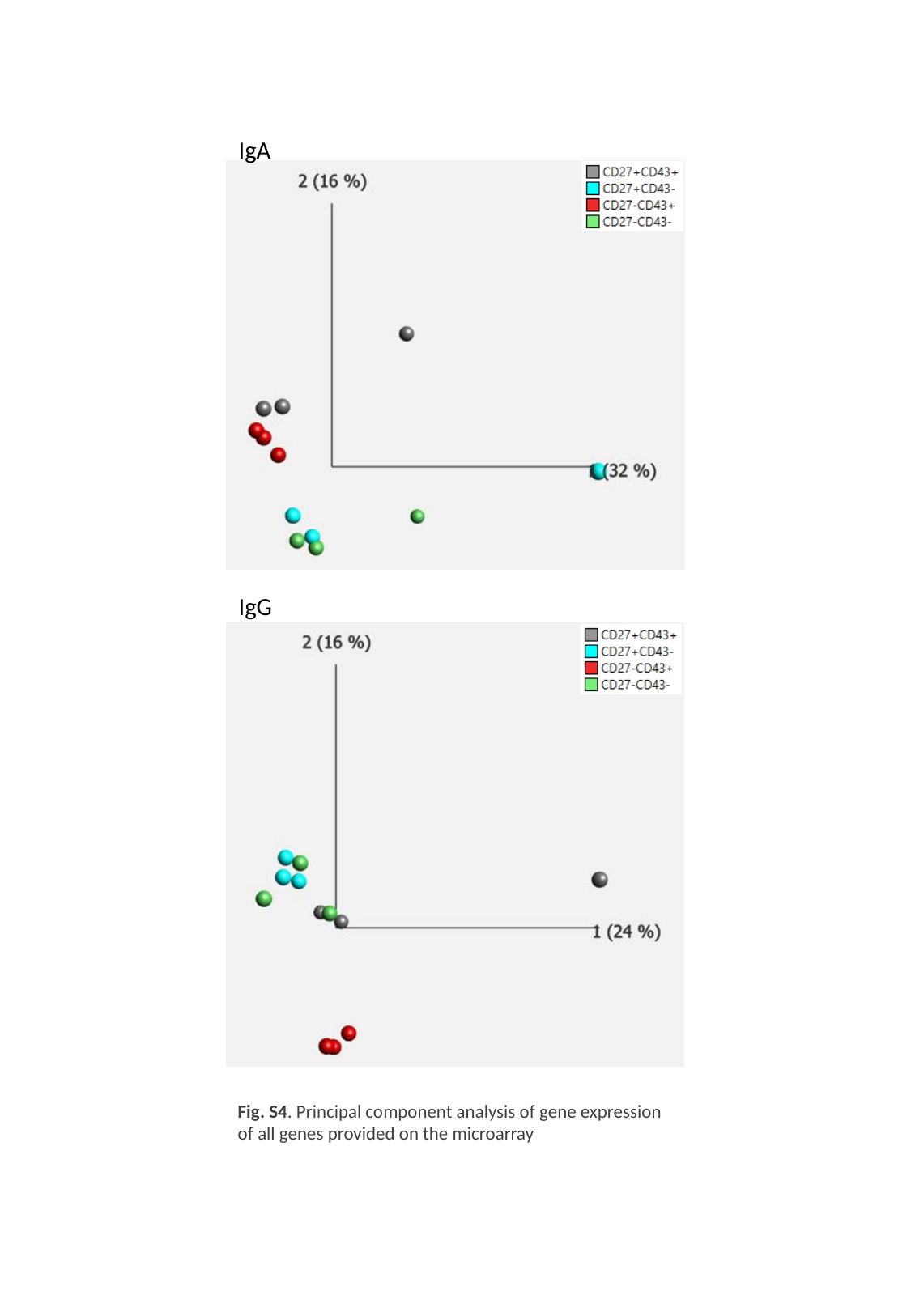

IgA
IgG
Fig. S4. Principal component analysis of gene expression of all genes provided on the microarray

## Slide 6
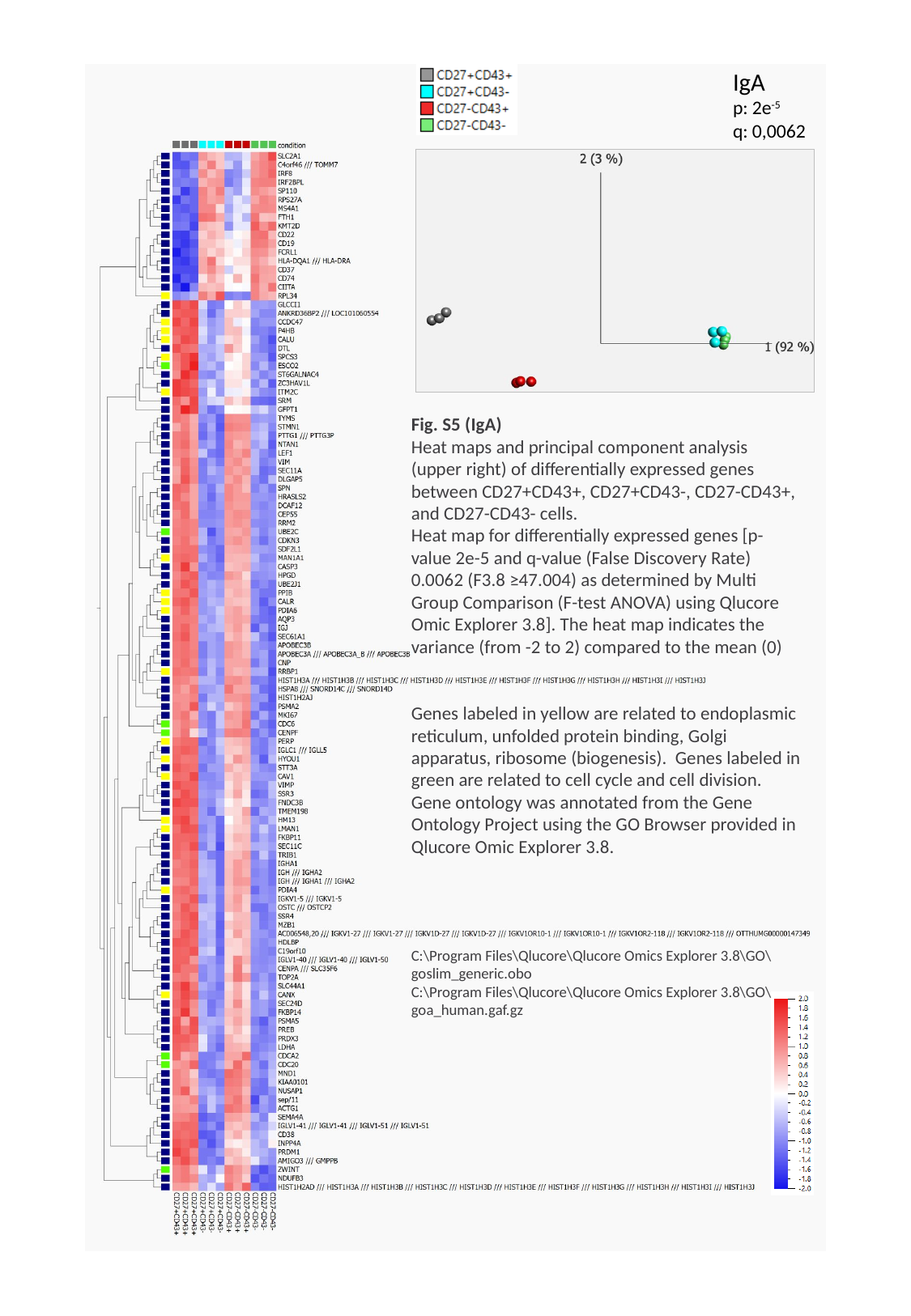

IgA
p: 2e-5
q: 0,0062
Fig. S5 (IgA)
Heat maps and principal component analysis (upper right) of differentially expressed genes between CD27+CD43+, CD27+CD43-, CD27-CD43+, and CD27-CD43- cells.
Heat map for differentially expressed genes [p-value 2e-5 and q-value (False Discovery Rate) 0.0062 (F3.8 ≥47.004) as determined by Multi Group Comparison (F-test ANOVA) using Qlucore Omic Explorer 3.8]. The heat map indicates the variance (from -2 to 2) compared to the mean (0)
Genes labeled in yellow are related to endoplasmic reticulum, unfolded protein binding, Golgi apparatus, ribosome (biogenesis). Genes labeled in green are related to cell cycle and cell division.
Gene ontology was annotated from the Gene Ontology Project using the GO Browser provided in Qlucore Omic Explorer 3.8.
C:\Program Files\Qlucore\Qlucore Omics Explorer 3.8\GO\goslim_generic.obo
C:\Program Files\Qlucore\Qlucore Omics Explorer 3.8\GO\goa_human.gaf.gz

## Slide 7
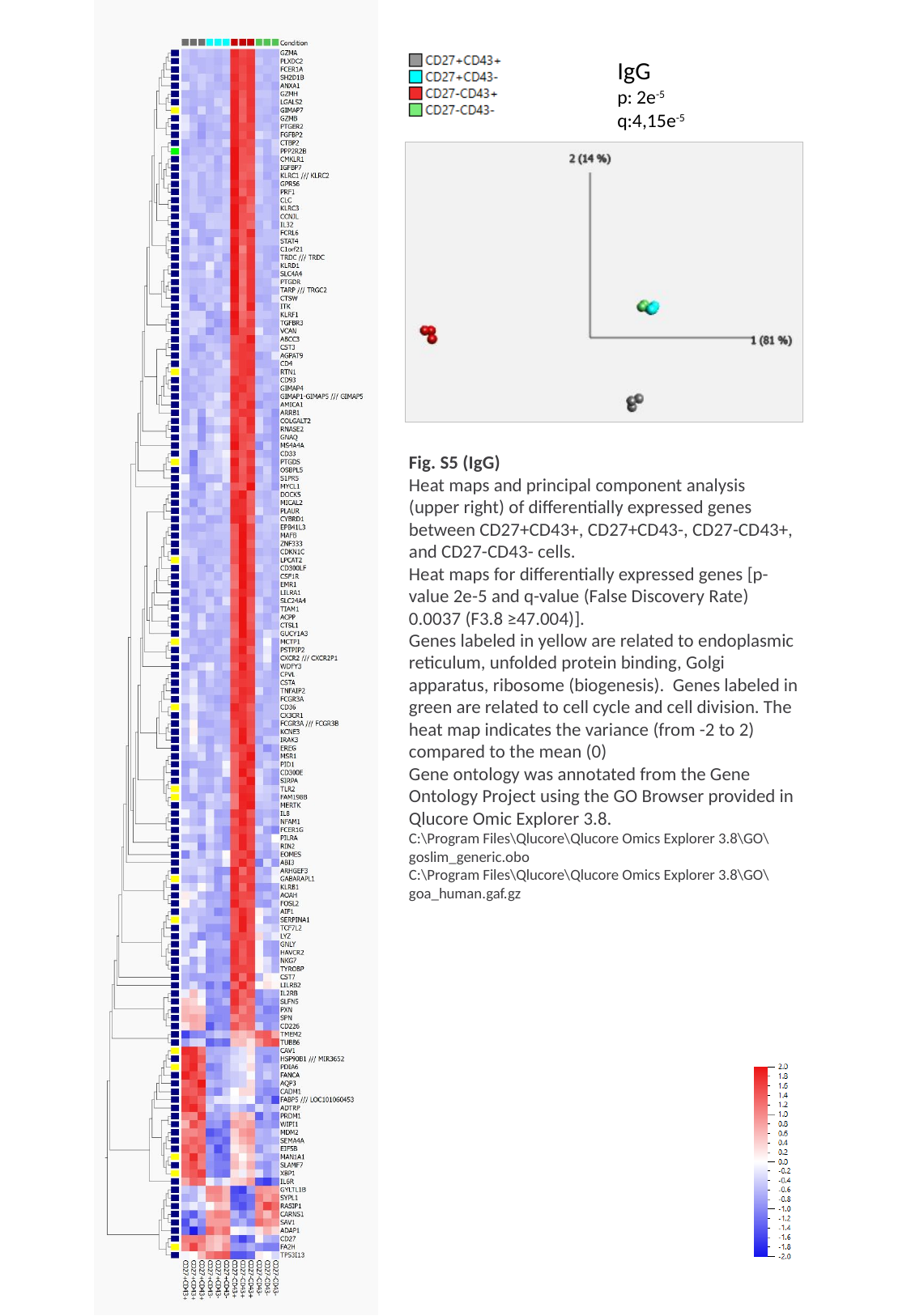

IgG
p: 2e-5
q:4,15e-5
Fig. S5 (IgG)
Heat maps and principal component analysis (upper right) of differentially expressed genes between CD27+CD43+, CD27+CD43-, CD27-CD43+, and CD27-CD43- cells.
Heat maps for differentially expressed genes [p-value 2e-5 and q-value (False Discovery Rate) 0.0037 (F3.8 ≥47.004)].
Genes labeled in yellow are related to endoplasmic reticulum, unfolded protein binding, Golgi apparatus, ribosome (biogenesis). Genes labeled in green are related to cell cycle and cell division. The heat map indicates the variance (from -2 to 2) compared to the mean (0)
Gene ontology was annotated from the Gene Ontology Project using the GO Browser provided in Qlucore Omic Explorer 3.8.
C:\Program Files\Qlucore\Qlucore Omics Explorer 3.8\GO\goslim_generic.obo
C:\Program Files\Qlucore\Qlucore Omics Explorer 3.8\GO\goa_human.gaf.gz

## Slide 8
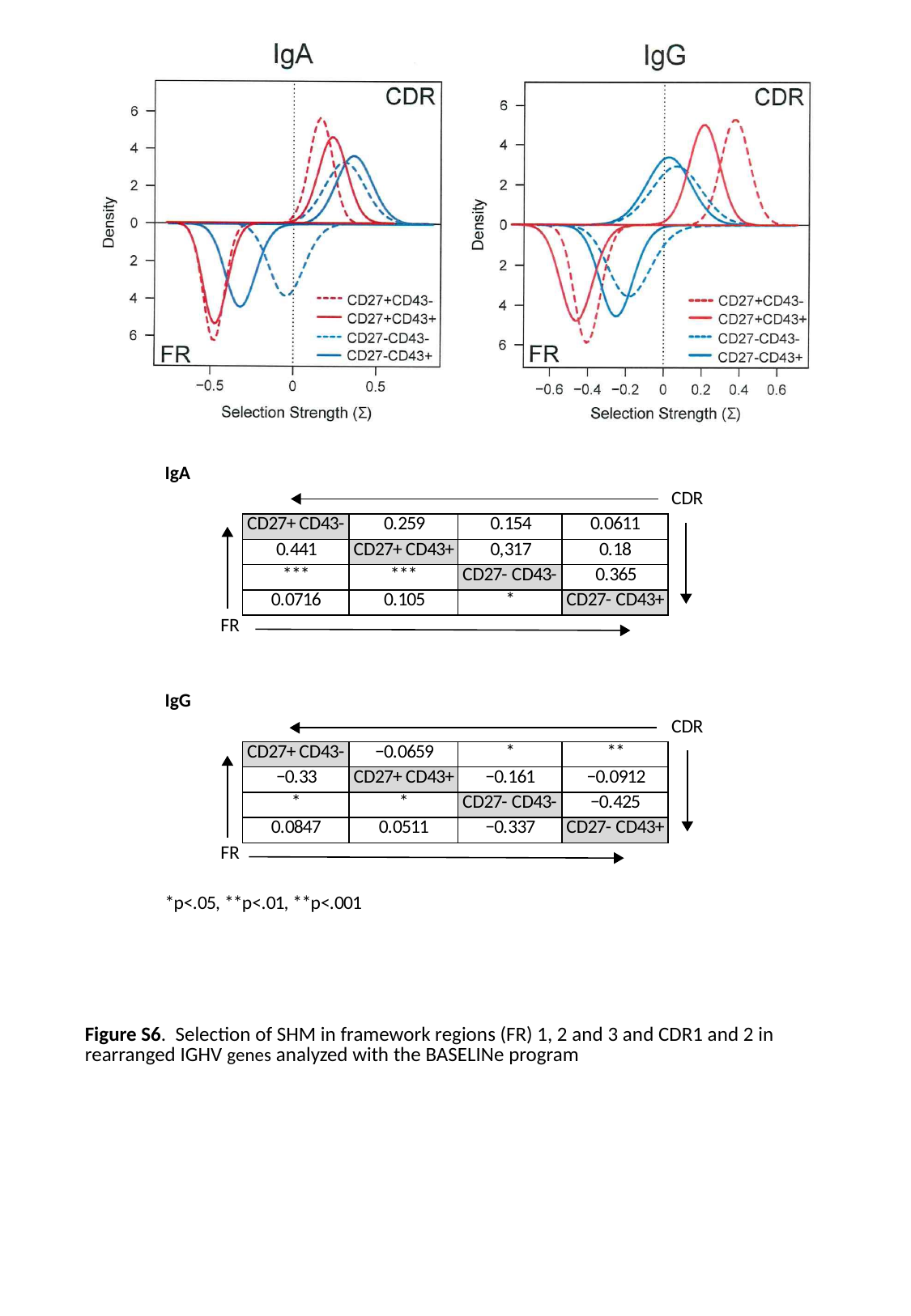

Figure S6. Selection of SHM in framework regions (FR) 1, 2 and 3 and CDR1 and 2 in rearranged IGHV genes analyzed with the BASELINe program

## Slide 9
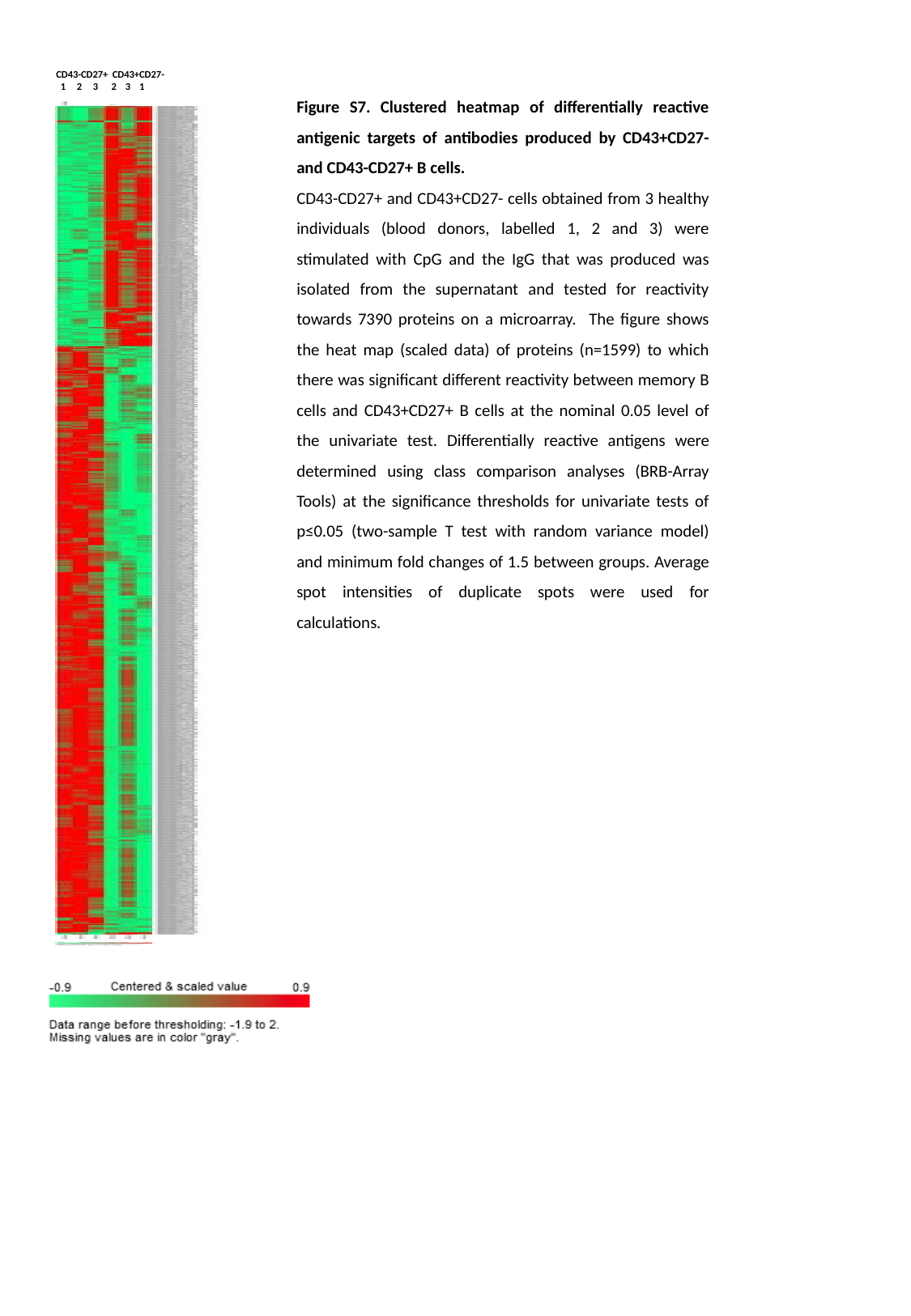

CD43-CD27+ CD43+CD27-
 1 2 3 2 3 1
Figure S7. Clustered heatmap of differentially reactive antigenic targets of antibodies produced by CD43+CD27- and CD43-CD27+ B cells.
CD43-CD27+ and CD43+CD27- cells obtained from 3 healthy individuals (blood donors, labelled 1, 2 and 3) were stimulated with CpG and the IgG that was produced was isolated from the supernatant and tested for reactivity towards 7390 proteins on a microarray. The figure shows the heat map (scaled data) of proteins (n=1599) to which there was significant different reactivity between memory B cells and CD43+CD27+ B cells at the nominal 0.05 level of the univariate test. Differentially reactive antigens were determined using class comparison analyses (BRB-Array Tools) at the significance thresholds for univariate tests of p≤0.05 (two-sample T test with random variance model) and minimum fold changes of 1.5 between groups. Average spot intensities of duplicate spots were used for calculations.

## Slide 10
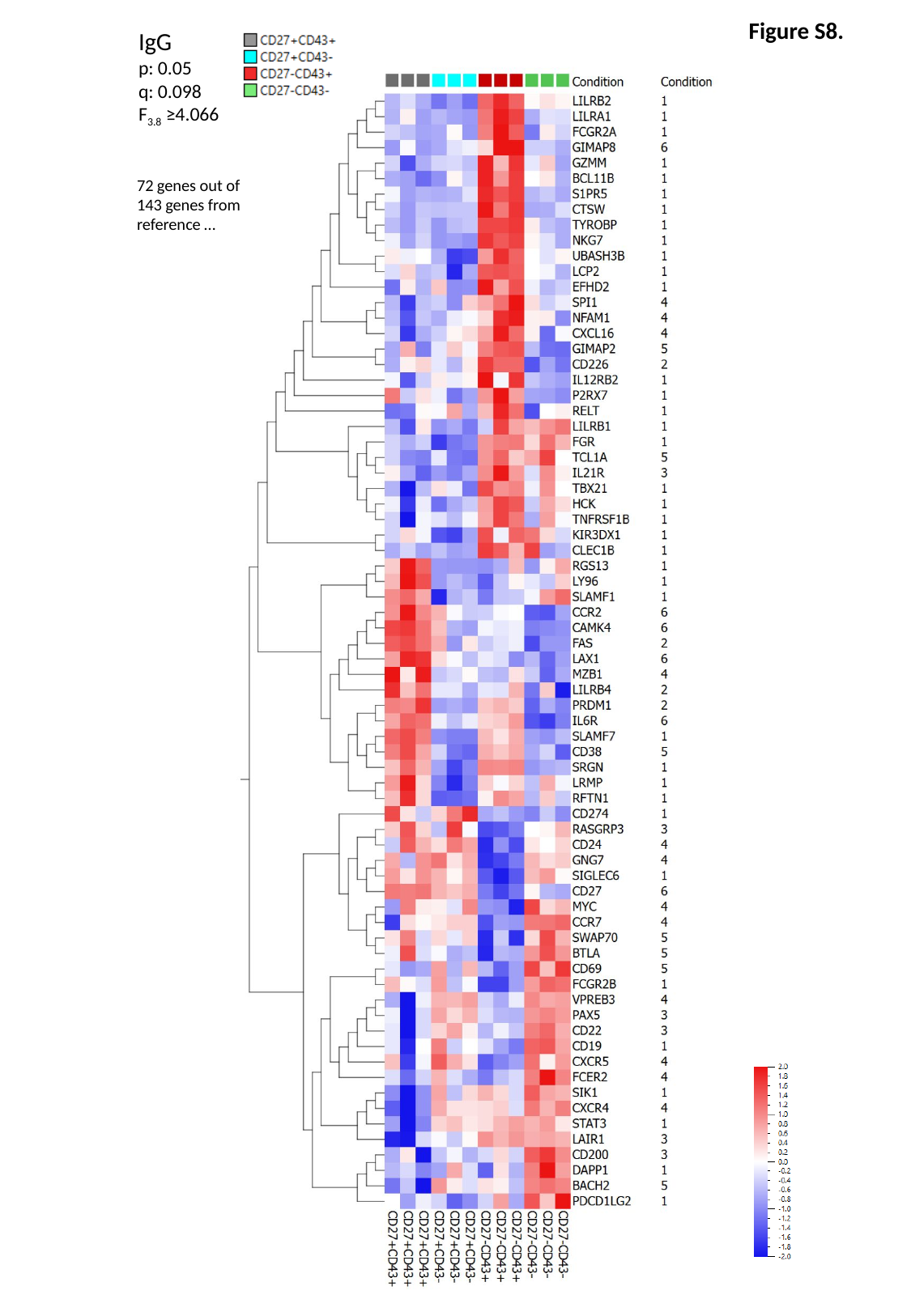

Figure S8.
IgG
p: 0.05
q: 0.098
F3.8 ≥4.066
72 genes out of 143 genes from reference …

## Slide 11
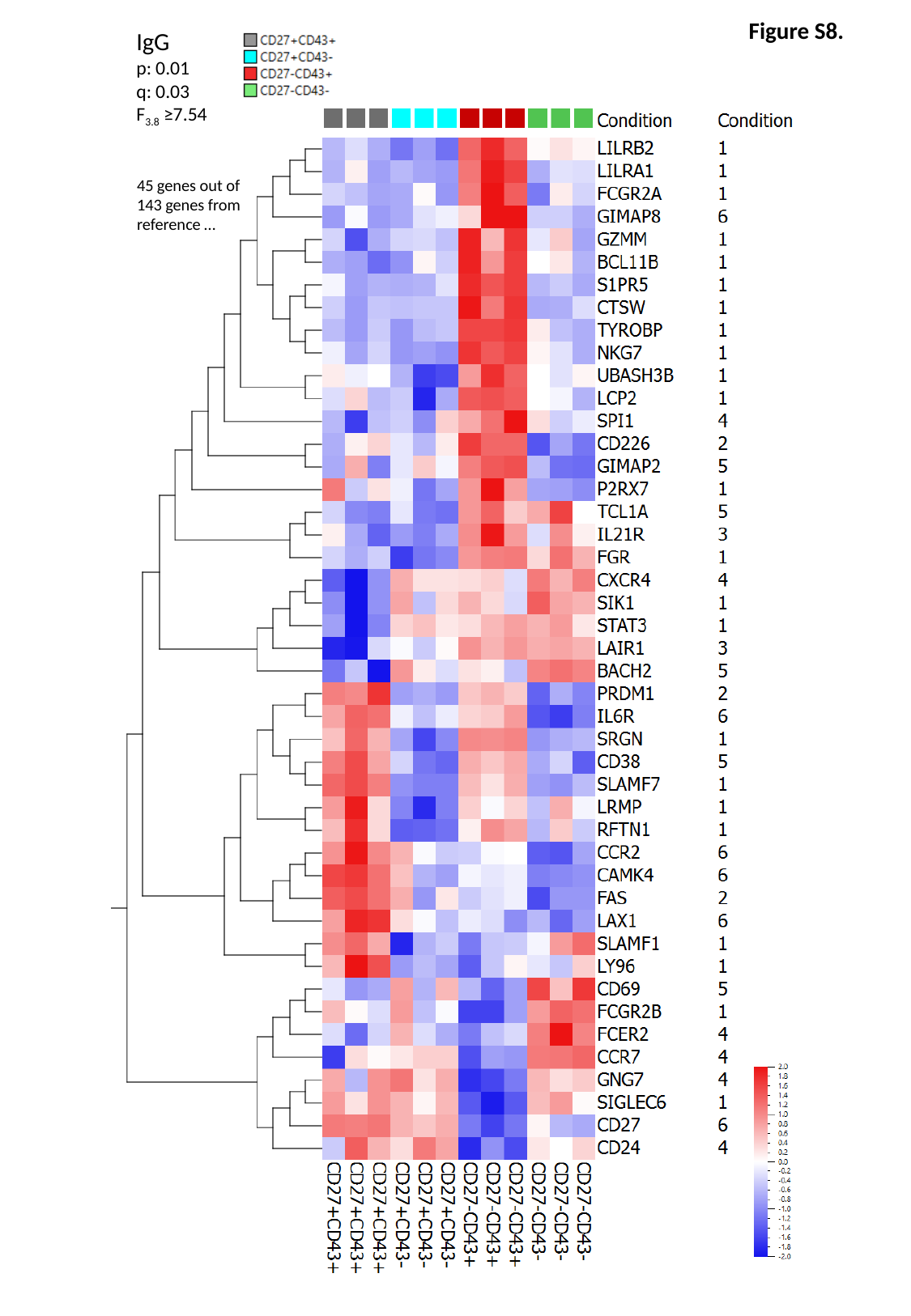

Figure S8.
IgG
p: 0.01
q: 0.03
F3.8 ≥7.54
45 genes out of 143 genes from reference …

## Slide 12
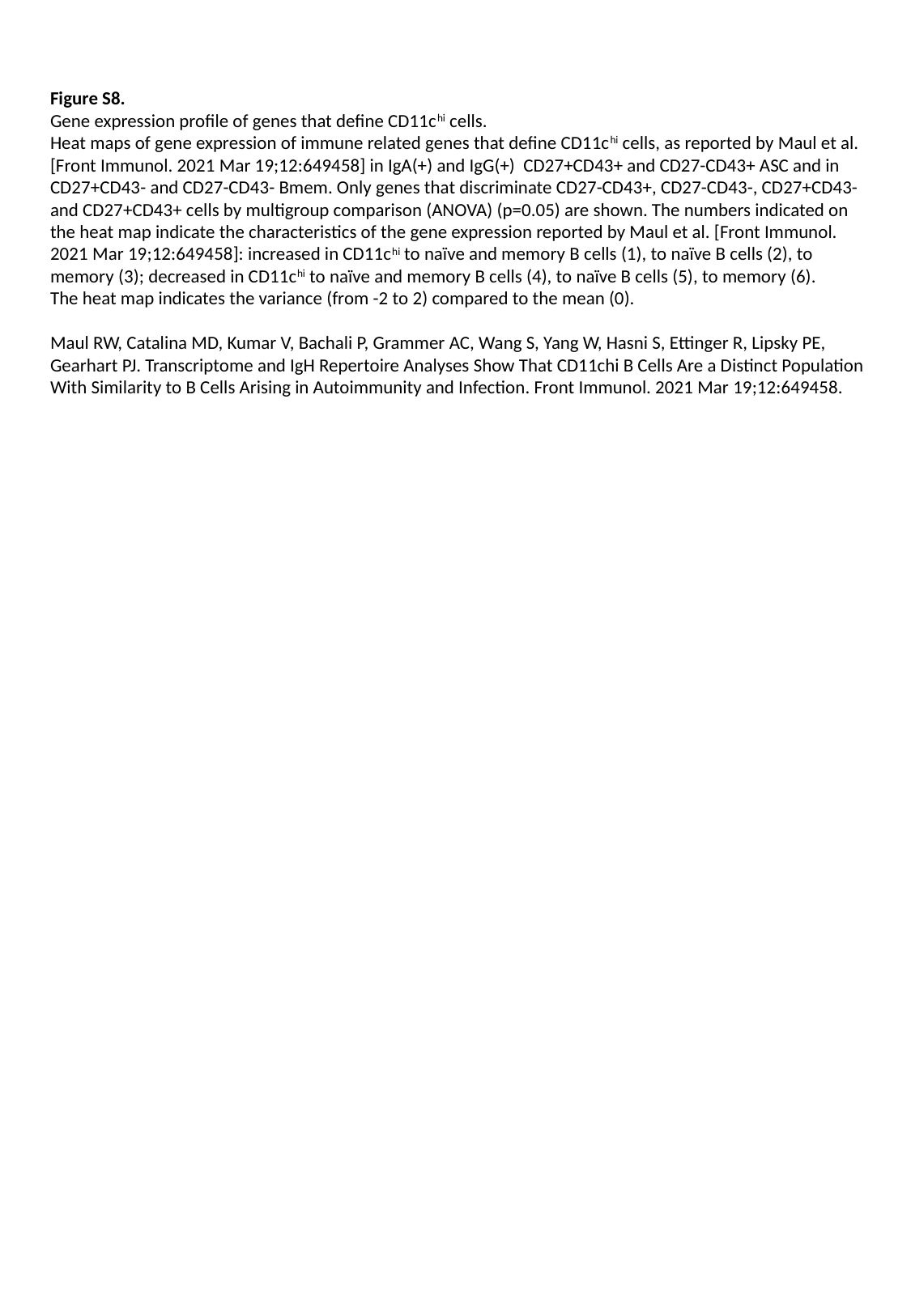

Figure S8.
Gene expression profile of genes that define CD11chi cells.
Heat maps of gene expression of immune related genes that define CD11chi cells, as reported by Maul et al. [Front Immunol. 2021 Mar 19;12:649458] in IgA(+) and IgG(+) CD27+CD43+ and CD27-CD43+ ASC and in CD27+CD43- and CD27-CD43- Bmem. Only genes that discriminate CD27-CD43+, CD27-CD43-, CD27+CD43- and CD27+CD43+ cells by multigroup comparison (ANOVA) (p=0.05) are shown. The numbers indicated on the heat map indicate the characteristics of the gene expression reported by Maul et al. [Front Immunol. 2021 Mar 19;12:649458]: increased in CD11chi to naïve and memory B cells (1), to naïve B cells (2), to memory (3); decreased in CD11chi to naïve and memory B cells (4), to naïve B cells (5), to memory (6).
The heat map indicates the variance (from -2 to 2) compared to the mean (0).
Maul RW, Catalina MD, Kumar V, Bachali P, Grammer AC, Wang S, Yang W, Hasni S, Ettinger R, Lipsky PE, Gearhart PJ. Transcriptome and IgH Repertoire Analyses Show That CD11chi B Cells Are a Distinct Population With Similarity to B Cells Arising in Autoimmunity and Infection. Front Immunol. 2021 Mar 19;12:649458.

## Slide 13
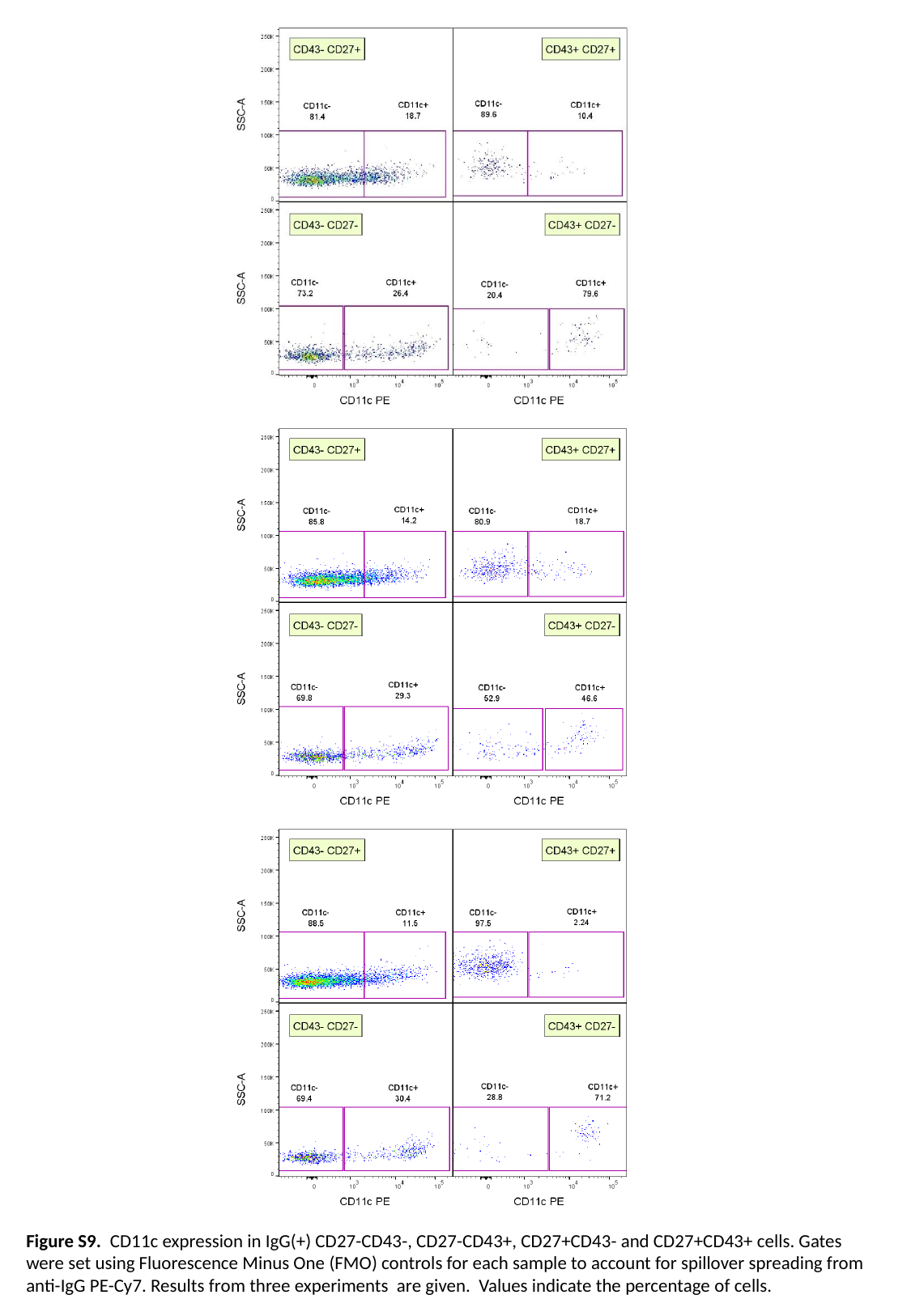

Figure S9. CD11c expression in IgG(+) CD27-CD43-, CD27-CD43+, CD27+CD43- and CD27+CD43+ cells. Gates were set using Fluorescence Minus One (FMO) controls for each sample to account for spillover spreading from anti-IgG PE-Cy7. Results from three experiments are given. Values indicate the percentage of cells.
