# Supplementary material for: Plasma cells are not restricted to the CD27+ phenotype: characterization of CD27-CD43+ antibody-secreting cells
Source: Front Immunol. 2023 Jul 10;14:1165936. doi: 10.3389/fimmu.2023.1165936 (PMC10364057; doi:10.3389/fimmu.2023.1165936)
Supplement: Supplementary file 2 [file Table_1.docx]

Supplemental data

**Table S1: List of fluorochrome-conjugated antibodies used in the study.**

| **Specificity** | **Clone** | **Fluorophore** | **Company** |
| --- | --- | --- | --- |
| CD3 | UCTH1 | PECF594 | BD Biosciences |
| CD19 | HIB19 | BV421 | Biolegend |
| CD19 | SJ25C1 | APC-H7 | BD Biosciences |
| CD27 | O323 | BV605 | Biolegend |
| CD43 | 1G10 | FITC | BD Biosciences |
| IgA | IS11-8E10 | APC | Miltenyi Biotech |
| IgG | G18-145 | PECy7 | BD Biosciences |
| IgM | MHM-88 | PERCP-Cy5,5 | Biolegend |
| IgD | IA6-2 | BV510 | Biolegend |
| CD38 | HIT2 | BV711 | Biolegend |
| CD184/CXCR4 | 15G5 | PE | Biolegend |
| CD20 | L27 | APC-H7 | BD Biosciences |
| CD24 | ML5 | PE | Beckman Coulter |
| β7INTEGRIN | FIB504 | PECy5 | BD Biosciences |
| CD138 | B-A38 | PE | IQ Products |
| CD14 | MϕP9 | PECF594 | BD Biosciences |
| CD56 | NCAM16.2 | PECF594 | BD Biosciences |
| CD45 | HI30 | Alexa70 | BD Biosciences |
| CD11c | Bu15 | PE | BioLegend |

**Table S2: plasma-cell related genes**

| BACH2 | BANK1 | CD19 | CD27 | CD38 | CD59 | CIITA | CXCR3 | FCER2 |
| --- | --- | --- | --- | --- | --- | --- | --- | --- |
| IL6R | IRF4 | IRF8 | LDLR | MS4A1 | PAX5 | PRDM1 | SDC1 | SERPIN89 |
| XBP1 |  |  |  |  |  |  |  |  |

Source: Henn AD, Wu S, Qiu X, Ruda M, Stover M, Yang H, Liu Z, Welle SL, Holden-Wiltse J, Wu H, Zand MS. High-resolution temporal response patterns to influenza vaccine reveal a distinct human plasma cell gene signature. Sci Rep. 2013;3:2327. (see also Fig. 3)

|  |  |  |  |  |  |  |  |  |
| --- | --- | --- | --- | --- | --- | --- | --- | --- |
| ADA | CCDC117 | DNAJB9 | GLIPR1 | LMAN1 | PPAPDC1B | SEC24D | SRPRB | TRABD |
| ADK | CCPG1 | DNAJC3 | GLT8D1 | LRRC59 | PPP1R15A | SEC61A1 | SSR1 | TRAM2 |
| ALAD | CD28 | DPAGT1 | GNE | MAGED1 | PQLC3 | SEC61B | SSR2 | TRIB1 |
| ALCAM | CD93 | EAF2 | GPR155 | MAGT1 | PRAF2 | SEC63 | SSR3 | TSSC4 |
| ALDH18A1 | CDV3 | EDEM1 | GPR55 | MAN1B1 | PRDM1 | SEL1L | SSR4 | TVP23B |
| ALDH9A1 | CHID1 | EDEM2 | H1F0 | MANEA | PRDX4 | SELK | ST6GAL1 | TXNDC11 |
| ALG2 | CHPF | EDEM3 | HDLBP | MANF | PRG2 | SELM | STT3A | UBA5 |
| AP2M1 | CHST1 | ELL2 | HERPUD1 | MARS | PRMT7 | SEPP1 | SUMO2 | UBC |
| ARF4 | CITED2 | ENDOU | HID1 | MCFD2 | PRRC1 | SERINC5 | SURF4 | UBE2J1 |
| ARFGAP3 | CKAP4 | ENPP1 | HIST1H1C | METTL9 | QPCTL | SIL1 | TAPBPL | UBXN4 |
| ASNS | CLPTM1L | ENTPD1 | HM13 | MGAT2 | RAB1A | SLAMF7 | TIGIT | UFC1 |
| ATAT1 | CLTB | EPCAM | HMGA1 | MIR631 | RAPGEF3 | SLC12A4 | TMED2 | USO1 |
| ATF5 | CNPY2 | ERGIC1 | HSD11B1 | MORF4L2 | RCBTB2 | SLC33A1 | TMED3 | VCP |
| ATL2 | CNST | ERN1 | HSPA13 | MRP63 | RELN | SLC39A11 | VIMP | WIPI1 |
| ATP6V0A1 | COPE | ERP44 | HSPA13 | MTDH | REXO2 | SLC39A4 | WBP5 | XBP1 |
| BCKDK | CORO2B | FAM214A | HSPA5 | MTHFD2 | RGCC | SLC39A7 | TMED9 | YARS |
| BET1 | CPEB2 | FAM46C | HTATIP2 | MZB1 | RHOB | SLC3A2 | TMEM176A | YIPF2 |
| BHLHA15 | CPOX | FCER1G | HYOU1 | NANS | RHOBTB1 | SLC44A1 | TMEM176B | YIPF5 |
| BLOC1S5-TXNDC5 | CREB3L2 | FKBP11 | IDH2 | NARS | RPL10 | SLC7A5 | TMEM184B | YIPF6 |
| BTD | CREG1 | FKBP2 | IFI16 | NDUFA1 | RPL15 | SLPI | TMEM214 | ZBP1 |
| C10orf54 | CRELD2 | FNDC3A | IFT20 | NUCB1 | RPN1 | SND1 | TMEM248 | ZFYVE21 |
| C15orf39 | CTSE | FNDC3B | IQCB1 | NUDT22 | RPN2 | SNX9 | TMEM258 | ZNF280B |
| C19orf10 | DAP | FOS | IRF4 | OS9 | RPS27L | SPCS1 | TMEM39A | |
| C1orf27 | DDOST | FUT1 | ISG20 | OXCT1 | RPS6 | SPCS2 | TMEM66 |  |
| CACNA1H | DENND5B | FUT8 | ITM2C | P4HB | SDC1 | SPCS3 | TNFRSF17 |  |
| CALR | DERL1 | GALNT2 | KCNN4 | PCK2 | SDF2L1 | SPINT2 | TNS3 |  |
| CALU | DERL3 | GFPT1 | KDELR1 | PDIA6 | SEC11C | SPON1 | TOP1 |  |
| CAPN5 | DESI1 | GLB1 | KRTCAP2 | PON2 | SEC22B | SRP54 | TP53INP1 |  |
| CARS | DHDDS |  | LEPROTL1 | PON3 | SEC24A | SRPR | TPST1 |  |
|  |  |  |  |  |  |  |  |  |

Source: Shi W, Liao Y, Willis SN, Taubenheim N, Inouye M, Tarlinton DM, Smyth GK, Hodgkin PD, Nutt SL, Corcoran LM.Transcriptional profiling of mouse B cell terminal differentiation defines a signature for antibody-secreting plasma cells. Nat Immunol. 2015;16:663-73. (see also Fig. 3)

**Table S3**. Gene expression in CD27- and CD27+ ASC of (i) Toll-like receptors, (ii) chemokines and their receptors, and (iii) interleukins and their receptors. Data are from a single experiment including three different donors. Analysis of differential expression was done with the lmFit function contained in the R-package LIMMA (see Materials and Methods). To correct for multiple comparisons, adjusted P-values were obtained by a Benjamini-Hochberg correction of the p-values.

|  |  | **IgG** |  |  |  |  | **IgA** |  |  |  |  |
| --- | --- | --- | --- | --- | --- | --- | --- | --- | --- | --- | --- |
| Probe Set | Gene symbol | Av. Expr. CD27+ | Av. Expr. CD27- | log Fold Change | P.Value | adj.P.Val | Av. Expr. CD27+ | Av. Expr. CD27- | log Fold Change | P.Value | adj.P.Val |
| **Toll Like Receptors** | |  |  |  |  |  |  |  |  |  |  |
| 11758377_s_at | TLR1 | 7,76 | 8,03 | 0,27 | 0.5212 | 0.8138 | 7,04 | 8,01 | 0,97 | 0.0732 | 0.9125 |
| 11746407_x_at | TLR10 | 8,12 | 6,73 | -1,38 | **0.0007** | **0.0266** | 7,49 | 8,46 | 0,96 | 0.0584 | 0.8887 |
| 11746534_a_at | TLR10 | 8,46 | 7,13 | -1,33 | **0.0026** | 0.0592 | 10,11 | 10,63 | 0,52 | 0.0659 | 0.9085 |
| 11746406_a_at | TLR10 | 10,80 | 9,97 | -0,83 | **0.0171** | 0.1738 | 7,17 | 8,26 | 1,10 | 0.0726 | 0.9125 |
| 11736273_a_at | TLR2 | 2,91 | 6,21 | 3,30 | **0.0000** | **0.0000** | 2,52 | 2,95 | 0,43 | 0.1034 | 0.9360 |
| 11736274_at | TLR2 | 3,33 | 5,21 | 1,88 | **0.0000** | **0.0022** | 4,25 | 3,83 | -0,42 | 0.2642 | 0.9676 |
| 11754833_a_at | TLR2 | 4,69 | 9,04 | 4,35 | **0.0002** | **0.0117** | 3,38 | 4,12 | 0,74 | 0.3693 | 0.9689 |
| 11730296_a_at | TLR3 | 3,57 | 2,84 | -0,73 | 0.0827 | 0.3846 | 2,69 | 2,76 | 0,07 | 0.7047 | 0.9868 |
| 11747518_a_at | TLR4 | 3,36 | 6,87 | 3,51 | **0.0001** | **0.0093** | 3,46 | 3,85 | 0,39 | 0.3374 | 0.9689 |
| 11743196_a_at | TLR4 | 5,22 | 7,60 | 2,38 | **0.0002** | **0.0118** | 6,46 | 6,16 | -0,29 | 0.5473 | 0.9715 |
| 11743197_at | TLR4 | 7,91 | 9,52 | 1,61 | 0.0551 | 0.3162 | 6,05 | 6,05 | 0,00 | 0.9965 | 0.9997 |
| 11731200_a_at | TLR5 | 4,19 | 5,89 | 1,70 | **0.0014** | **0.0397** | 4,47 | 4,34 | -0,13 | 0.7595 | 0.9902 |
| 11737628_a_at | TLR6 | 6,69 | 5,99 | -0,69 | 0.0850 | 0.3899 | 6,73 | 7,26 | 0,53 | 0.4474 | 0.9689 |
| 11737629_s_at | TLR6 | 8,66 | 8,61 | -0,05 | 0.8981 | 0.9709 | 9,15 | 9,32 | 0,16 | 0.6043 | 0.9767 |
| 11731147_at | TLR7 | 6,78 | 5,45 | -1,33 | **0.0153** | 0.1635 | 6,69 | 6,33 | -0,36 | 0.5705 | 0.9753 |
| 11731148_at | TLR7 | 7,42 | 6,35 | -1,08 | **0.0357** | 0.2545 | 7,11 | 7,25 | 0,14 | 0.7958 | 0.9918 |
| 11726947_a_at | TLR8 | 4,24 | 7,41 | 3,17 | **0.0000** | **0.0038** | 3,52 | 3,28 | -0,24 | 0.3361 | 0.9689 |
| 11726948_at | TLR8 | 3,30 | 5,56 | 2,26 | **0.0002** | **0.0119** | 3,89 | 3,77 | -0,12 | 0.5445 | 0.9715 |
| 11716547_s_at | TLR9 | 6,66 | 6,36 | -0,30 | 0.3345 | 0.6909 | 6,90 | 7,07 | 0,18 | 0.5210 | 0.9698 |
|  |  |  |  |  |  |  |  |  |  |  |  |
| **Chemokine receptors and their ligands** | |  |  |  |  |  |  |  |  |  |  |
| 11720994_x_at | CCL3 /// LOC101060267 | 7,55 | 9,26 | 1,71 | 0.2154 | 0.5788 | 8,90 | 7,11 | -1,78 | **0.0014** | 0.4030 |
| 11755564_x_at | CCL3L1 /// CCL3L3 | 4,57 | 6,41 | 1,84 | **0.0085** | 0.1194 | 5,31 | 4,52 | -0,79 | **0.0297** | 0.7958 |
| 11746954_s_at | CCL4 /// CCL4L1 /// CCL4L2 /// LOC101060278 | 5,54 | 10,65 | 5,11 | **0.0000** | **0.0005** | 6,01 | 5,20 | -0,81 | 0.1354 | 0.9484 |
| 11718983_x_at | CCL4 /// CCL4L1 /// CCL4L2 /// LOC101060278 | 5,17 | 9,66 | 4,49 | **0.0000** | **0.0015** | 6,96 | 6,15 | -0,80 | 0.1736 | 0.9672 |
| 11718982_s_at | CCL4 /// CCL4L1 /// CCL4L2 /// LOC101060278 | 6,33 | 11,10 | 4,78 | **0.0002** | **0.0108** | 5,69 | 5,16 | -0,54 | 0.2597 | 0.9676 |
| 11744660_s_at | CCL4L1 /// CCL4L2 | 3,07 | 4,86 | 1,79 | **0.0007** | **0.0249** | 5,39 | 5,08 | -0,31 | 0.3220 | 0.9689 |
| 11759870_x_at | CCL4L1 /// CCL4L2 | 4,57 | 5,94 | 1,37 | **0.0036** | 0.0720 | 2,71 | 2,59 | -0,12 | 0.5105 | 0.9698 |
| 11763012_x_at | CCL4L1 /// CCL4L2 | 4,60 | 6,06 | 1,46 | **0.0041** | 0.0782 | 5,00 | 4,94 | -0,06 | 0.8310 | 0.9925 |
| 11760063_x_at | CCL4L1 /// CCL4L2 | 5,09 | 6,22 | 1,13 | **0.0219** | 0.1983 | 5,21 | 5,16 | -0,05 | 0.8750 | 0.9944 |
| 11732276_x_at | CCL5 | 8,83 | 12,93 | 4,10 | **0.0002** | **0.0129** | 4,60 | 5,62 | 1,02 | **0.0072** | 0.6095 |
| 11732275_at | CCL5 | 6,42 | 10,50 | 4,09 | **0.0005** | **0.0214** | 6,91 | 8,39 | 1,48 | **0.0033** | 0.4892 |
| 11753810_a_at | CCL5 | 9,04 | 13,26 | 4,21 | **0.0006** | **0.0221** | 7,25 | 8,96 | 1,71 | **0.0006** | 0.3621 |
| 11729847_a_at | CCL7 | 2,90 | 2,83 | -0,07 | 0.7494 | 0.9211 | 3,17 | 3,19 | 0,01 | 0.9456 | 0.9979 |
| 11728038_at | CCL8 | 3,28 | 3,39 | 0,11 | 0.6273 | 0.8685 | 3,23 | 3,09 | -0,14 | 0.5090 | 0.9698 |
| 11728039_s_at | CCL8 | 2,95 | 2,90 | -0,05 | 0.8093 | 0.9417 | 2,82 | 2,82 | 0,00 | 0.9951 | 0.9997 |
| 11725444_at | CCR1 | 6,06 | 6,17 | 0,12 | 0.8007 | 0.9386 | 5,67 | 6,31 | 0,65 | 0.2449 | 0.9676 |
| 11729900_at | CCR10 | 5,06 | 4,78 | -0,28 | 0.2801 | 0.6457 | 6,99 | 6,19 | -0,80 | **0.0384** | 0.8397 |
| 11731676_s_at | CCR2 | 7,09 | 5,24 | -1,85 | **0.0016** | **0.0447** | 6,42 | 4,55 | -1,87 | **0.0007** | 0.3667 |
| 11750856_s_at | CCR2 | 5,47 | 3,62 | -1,85 | **0.0368** | 0.2583 | 5,25 | 4,23 | -1,02 | 0.0746 | 0.9132 |
| 11752590_s_at | CCR2 | 4,03 | 3,32 | -0,71 | 0.1031 | 0.4246 | 4,41 | 3,43 | -0,98 | **0.0057** | 0.5785 |
| 11750769_s_at | CCR2 | 6,60 | 5,43 | -1,17 | 0.2367 | 0.6016 | 4,67 | 3,72 | -0,95 | **0.0292** | 0.7945 |
| 11740646_a_at | CCR3 | 3,38 | 3,99 | 0,61 | **0.0397** | 0.2693 | 5,17 | 4,10 | -1,08 | 0.0875 | 0.9247 |
| 11741322_a_at | CCR3 | 5,38 | 6,00 | 0,61 | **0.0445** | 0.2838 | 5,36 | 5,38 | 0,02 | 0.9431 | 0.9979 |
| 11736720_at | CCR4 | 3,32 | 3,99 | 0,68 | **0.0070** | 0.1063 | 3,84 | 3,94 | 0,10 | 0.5877 | 0.9767 |
| 11759215_at | CCR4 | 3,22 | 3,32 | 0,11 | 0.6146 | 0.8623 | 2,48 | 2,65 | 0,17 | 0.3705 | 0.9689 |
| 11730910_s_at | CCR5 | 4,06 | 3,83 | -0,23 | 0.3931 | 0.7348 | 3,53 | 3,37 | -0,17 | 0.8068 | 0.9925 |
| 11748062_s_at | CCR5 | 3,72 | 3,45 | -0,27 | 0.4695 | 0.7844 | 3,88 | 3,87 | -0,01 | 0.9714 | 0.9987 |
| 11730909_s_at | CCR5 | 4,74 | 4,97 | 0,22 | 0.7180 | 0.9077 | 4,88 | 4,95 | 0,07 | 0.8190 | 0.9925 |
| 11736620_x_at | CCR6 | 6,08 | 4,80 | -1,28 | 0.0887 | 0.3968 | 4,58 | 4,85 | 0,27 | 0.6157 | 0.9786 |
| 11752105_x_at | CCR6 | 7,70 | 7,07 | -0,63 | 0.2627 | 0.6293 | 6,40 | 7,76 | 1,35 | **0.0132** | 0.6665 |
| 11736621_a_at | CCR6 | 6,61 | 6,52 | -0,09 | 0.8160 | 0.9439 | 5,46 | 6,91 | 1,45 | **0.0038** | 0.5155 |
| 11725981_at | CCR7 | 8,73 | 7,98 | -0,74 | 0.1702 | 0.5264 | 7,43 | 8,65 | 1,22 | **0.0066** | 0.5941 |
| 11735871_s_at | CCR8 | 3,74 | 3,59 | -0,15 | 0.5243 | 0.8157 | 3,80 | 3,86 | 0,06 | 0.7613 | 0.9904 |
| 11732390_a_at | CCR9 | 6,40 | 6,36 | -0,04 | 0.9491 | 0.9855 | 8,25 | 8,82 | 0,57 | 0.1855 | 0.9674 |
| 11737108_a_at | CCRL1 | 3,94 | 4,39 | 0,44 | 0.2316 | 0.5963 | 7,28 | 6,32 | -0,95 | 0.1320 | 0.9459 |
| 11741285_a_at | CCRL1 | 4,53 | 5,68 | 1,15 | 0.2342 | 0.5986 | 6,14 | 5,60 | -0,54 | 0.5722 | 0.9754 |
| 11741286_a_at | CCRL1 | 4,49 | 4,15 | -0,34 | 0.5290 | 0.8184 | 6,01 | 5,72 | -0,29 | 0.5260 | 0.9698 |
| 11748896_s_at | CCRL1 | 5,12 | 5,03 | -0,09 | 0.8649 | 0.9604 | 5,43 | 5,76 | 0,34 | 0.3063 | 0.9689 |
| 11741990_s_at | CCRL2 | 3,87 | 4,12 | 0,25 | 0.4747 | 0.7869 | 4,05 | 4,41 | 0,36 | 0.1536 | 0.9573 |
| 11729424_s_at | CCRL2 | 5,22 | 5,27 | 0,05 | 0.9188 | 0.9770 | 3,29 | 3,88 | 0,59 | **0.0317** | 0.8090 |
| 11734552_a_at | CX3CL1 | 5,08 | 5,35 | 0,27 | 0.2597 | 0.6266 | 6,19 | 5,94 | -0,25 | 0.3544 | 0.9689 |
| 11734551_a_at | CX3CL1 | 5,53 | 5,56 | 0,03 | 0.8561 | 0.9576 | 5,81 | 5,64 | -0,17 | 0.4433 | 0.9689 |
| 11723048_at | CX3CR1 | 3,52 | 11,25 | 7,73 | **0.0000** | **0.0000** | 3,08 | 3,08 | 0,00 | 0.9987 | 0.9997 |
| 11756847_a_at | CX3CR1 | 3,44 | 10,25 | 6,81 | **0.0000** | **0.0000** | 2,84 | 3,66 | 0,82 | 0.1663 | 0.9651 |
| 11754114_a_at | CXCL1 | 4,30 | 4,11 | -0,18 | 0.4008 | 0.7400 | 3,27 | 3,10 | -0,17 | 0.4085 | 0.9689 |
| 11719366_s_at | CXCL1 | 3,16 | 3,13 | -0,03 | 0.8927 | 0.9692 | 4,91 | 4,77 | -0,14 | 0.6656 | 0.9819 |
| 11763250_x_at | CXCL1 /// CXCL2 | 5,48 | 5,22 | -0,26 | 0.2957 | 0.6597 | 4,57 | 4,34 | -0,24 | 0.4151 | 0.9689 |
| 11720298_at | CXCL10 | 3,20 | 3,23 | 0,03 | 0.9148 | 0.9762 | 3,04 | 3,11 | 0,07 | 0.7477 | 0.9889 |
| 11732466_a_at | CXCL11 | 2,77 | 2,35 | -0,42 | 0.0562 | 0.3192 | 2,68 | 2,62 | -0,06 | 0.7577 | 0.9896 |
| 11749245_a_at | CXCL11 | 3,12 | 2,76 | -0,36 | 0.1688 | 0.5247 | 2,44 | 2,46 | 0,02 | 0.9122 | 0.9973 |
| 11732467_x_at | CXCL11 | 2,89 | 2,57 | -0,32 | 0.2274 | 0.5919 | 2,51 | 2,64 | 0,13 | 0.5017 | 0.9698 |
| 11720818_a_at | CXCL12 | 4,48 | 4,13 | -0,35 | 0.0687 | 0.3527 | 4,16 | 3,80 | -0,36 | 0.1950 | 0.9676 |
| 11720819_s_at | CXCL12 | 3,14 | 2,73 | -0,41 | 0.0856 | 0.3908 | 3,62 | 3,40 | -0,22 | 0.4753 | 0.9689 |
| 11718744_a_at | CXCL12 | 3,51 | 3,25 | -0,26 | 0.3438 | 0.6988 | 3,03 | 2,95 | -0,08 | 0.6889 | 0.9844 |
| 11753257_a_at | CXCL12 | 3,09 | 2,93 | -0,15 | 0.4597 | 0.7783 | 4,55 | 4,52 | -0,02 | 0.8999 | 0.9972 |
| 11718743_a_at | CXCL12 | 3,49 | 3,41 | -0,08 | 0.6608 | 0.8839 | 3,31 | 3,35 | 0,03 | 0.8574 | 0.9933 |
| 11720161_at | CXCL13 | 3,89 | 3,20 | -0,69 | 0.1910 | 0.5517 | 3,77 | 3,81 | 0,03 | 0.9075 | 0.9972 |
| 11720162_at | CXCL13 | 3,33 | 3,13 | -0,20 | 0.3290 | 0.6871 | 3,46 | 3,58 | 0,12 | 0.8155 | 0.9925 |
| 11717911_x_at | CXCL14 | 4,72 | 4,32 | -0,40 | 0.0874 | 0.3941 | 3,33 | 3,24 | -0,09 | 0.8942 | 0.9970 |
| 11717910_at | CXCL14 | 4,98 | 4,75 | -0,23 | 0.2812 | 0.6466 | 4,24 | 4,28 | 0,03 | 0.8768 | 0.9947 |
| 11756059_a_at | CXCL14 | 3,99 | 4,16 | 0,17 | 0.3803 | 0.7252 | 4,88 | 5,04 | 0,16 | 0.4741 | 0.9689 |
| 11717912_s_at | CXCL14 | 2,95 | 2,84 | -0,11 | 0.6184 | 0.8645 | 5,05 | 5,25 | 0,19 | 0.3517 | 0.9689 |
| 11756645_x_at | CXCL16 | 6,87 | 10,11 | 3,24 | **0.0005** | **0.0204** | 6,75 | 6,93 | 0,18 | 0.7082 | 0.9868 |
| 11739280_a_at | CXCL16 | 6,66 | 9,12 | 2,45 | **0.0058** | 0.0951 | 5,18 | 5,89 | 0,72 | 0.1029 | 0.9357 |
| 11722658_at | CXCL17 | 5,34 | 5,33 | -0,01 | 0.9793 | 0.9945 | 5,94 | 4,78 | -1,16 | 0.0818 | 0.9238 |
| 11744127_at | CXCL2 | 3,29 | 6,27 | 2,98 | **0.0009** | **0.0299** | 3,52 | 3,26 | -0,27 | 0.3403 | 0.9689 |
| 11744128_x_at | CXCL2 | 3,38 | 6,14 | 2,76 | **0.0010** | **0.0330** | 3,01 | 3,08 | 0,07 | 0.8315 | 0.9925 |
| 11728477_at | CXCL3 | 2,61 | 3,07 | 0,46 | **0.0153** | 0.1636 | 2,67 | 2,54 | -0,13 | 0.5361 | 0.9701 |
| 11728476_a_at | CXCL3 | 4,13 | 3,47 | -0,66 | **0.0298** | 0.2334 | 3,82 | 3,70 | -0,11 | 0.6216 | 0.9792 |
| 11728716_x_at | CXCL5 | 2,60 | 3,64 | 1,04 | 0.0589 | 0.3267 | 2,68 | 2,50 | -0,18 | 0.4038 | 0.9689 |
| 11728715_at | CXCL5 | 2,64 | 3,57 | 0,94 | 0.0806 | 0.3797 | 2,54 | 2,43 | -0,11 | 0.5689 | 0.9747 |
| 11728717_at | CXCL5 | 2,68 | 3,08 | 0,40 | 0.0834 | 0.3864 | 2,47 | 2,42 | -0,05 | 0.8221 | 0.9925 |
| 11717025_s_at | CXCL5 /// GLYR1 | 9,21 | 9,65 | 0,44 | 0.1543 | 0.5062 | 9,37 | 9,54 | 0,17 | 0.6039 | 0.9767 |
| 11742670_at | CXCL6 | 3,63 | 3,47 | -0,16 | 0.4590 | 0.7777 | 2,82 | 2,33 | -0,49 | 0.1753 | 0.9672 |
| 11730801_at | CXCL6 | 2,27 | 2,20 | -0,07 | 0.6512 | 0.8795 | 3,43 | 3,42 | -0,01 | 0.9829 | 0.9990 |
| 11719943_at | CXCL9 | 2,95 | 2,97 | 0,03 | 0.9137 | 0.9758 | 3,19 | 3,24 | 0,06 | 0.7801 | 0.9907 |
| 11733003_a_at | CXCR1 | 5,49 | 6,50 | 1,01 | **0.0136** | 0.1536 | 5,84 | 5,68 | -0,16 | 0.5103 | 0.9698 |
| 11751323_a_at | CXCR1 | 4,59 | 4,95 | 0,36 | 0.1094 | 0.4360 | 4,44 | 4,47 | 0,03 | 0.9092 | 0.9972 |
| 11731424_x_at | CXCR2 | 3,10 | 7,81 | 4,71 | **0.0000** | **0.0002** | 4,75 | 4,22 | -0,52 | 0.1950 | 0.9676 |
| 11731423_at | CXCR2 | 3,09 | 7,61 | 4,53 | **0.0000** | **0.0007** | 3,36 | 2,97 | -0,39 | 0.5894 | 0.9767 |
| 11731425_at | CXCR2 | 4,10 | 6,98 | 2,88 | **0.0000** | **0.0036** | 3,83 | 3,53 | -0,30 | 0.6454 | 0.9807 |
| 11731426_s_at | CXCR2 /// CXCR2P1 | 5,33 | 9,67 | 4,34 | **0.0000** | **0.0000** | 5,90 | 5,27 | -0,63 | 0.2522 | 0.9676 |
| 11734606_a_at | CXCR3 | 6,99 | 6,15 | -0,84 | 0.2007 | 0.5628 | 7,74 | 6,65 | -1,09 | **0.0132** | 0.6665 |
| 11728191_x_at | CXCR4 | 11,83 | 12,75 | 0,92 | **0.0015** | **0.0410** | 12,62 | 12,70 | 0,08 | 0.6481 | 0.9808 |
| 11728189_a_at | CXCR4 | 11,41 | 12,33 | 0,92 | **0.0026** | 0.0588 | 11,57 | 12,14 | 0,57 | **0.0349** | 0.8250 |
| 11739094_a_at | CXCR4 | 11,34 | 12,41 | 1,07 | **0.0059** | 0.0959 | 11,31 | 11,91 | 0,61 | **0.0305** | 0.8007 |
| 11728190_s_at | CXCR4 | 12,58 | 13,02 | 0,44 | **0.0158** | 0.1666 | 11,31 | 11,96 | 0,65 | **0.0131** | 0.6665 |
| 11726966_a_at | CXCR5 | 10,25 | 9,45 | -0,80 | 0.0660 | 0.3455 | 9,73 | 10,32 | 0,58 | **0.0151** | 0.6825 |
| 11733116_a_at | CXCR5 | 7,28 | 6,57 | -0,71 | 0.2292 | 0.5938 | 7,68 | 8,27 | 0,58 | 0.0645 | 0.9081 |
| 11729977_a_at | CXCR6 | 4,28 | 5,14 | 0,86 | 0.1499 | 0.5004 | 6,29 | 5,57 | -0,73 | 0.2777 | 0.9676 |
| 11722598_s_at | CXCR7 | 4,80 | 6,49 | 1,68 | **0.0171** | 0.1739 | 5,91 | 5,49 | -0,42 | 0.0954 | 0.9299 |
| 11722597_a_at | CXCR7 | 5,79 | 6,03 | 0,23 | 0.2696 | 0.6361 | 6,49 | 6,23 | -0,26 | 0.2482 | 0.9676 |
| 11752501_a_at | CXCR7 | 5,59 | 5,79 | 0,21 | 0.3621 | 0.7114 | 6,64 | 6,40 | -0,24 | 0.2504 | 0.9676 |
| 11744541_a_at | CXCR7 | 6,38 | 6,42 | 0,04 | 0.8917 | 0.9690 | 5,28 | 5,26 | -0,01 | 0.9624 | 0.9983 |
| 11736722_at | XCL1 | 3,08 | 2,90 | -0,18 | 0.4720 | 0.7857 | 2,91 | 2,85 | -0,06 | 0.7645 | 0.9904 |
| 11735394_s_at | XCL1 /// XCL2 | 4,99 | 8,39 | 3,40 | **0.0001** | **0.0082** | 5,14 | 5,01 | -0,13 | 0.8363 | 0.9925 |
| 11738162_a_at | XCR1 | 4,24 | 3,85 | -0,39 | **0.0344** | 0.2493 | 4,15 | 4,26 | 0,12 | 0.6947 | 0.9854 |
|  |  |  |  |  |  |  |  |  |  |  |  |
| **Interleukines and their receptors** | |  |  |  |  |  |  |  |  |  |  |
| 11734307_at | IL10 | 3,55 | 3,08 | -0,48 | 0.0596 | 0.3287 | 3,31 | 3,34 | 0,03 | 0.8691 | 0.9934 |
| 11734308_at | IL10 | 4,35 | 4,19 | -0,16 | 0.4415 | 0.7671 | 4,40 | 4,41 | 0,01 | 0.9798 | 0.9988 |
| 11729747_a_at | IL10RA | 8,35 | 9,91 | 1,56 | **0.0083** | 0.1175 | 9,05 | 9,27 | 0,21 | 0.5158 | 0.9698 |
| 11729748_a_at | IL10RA | 6,56 | 6,76 | 0,21 | 0.6077 | 0.8585 | 6,92 | 6,79 | -0,13 | 0.7202 | 0.9873 |
| 11727467_x_at | IL10RB | 7,78 | 8,15 | 0,37 | 0.1123 | 0.4409 | 7,49 | 7,94 | 0,45 | 0.5648 | 0.9742 |
| 11753111_x_at | IL10RB | 7,56 | 8,27 | 0,71 | **0.0441** | 0.2826 | 7,77 | 8,06 | 0,30 | 0.3814 | 0.9689 |
| 11727690_at | IL11 | 3,35 | 3,49 | 0,14 | 0.4725 | 0.7860 | 3,64 | 3,54 | -0,09 | 0.5967 | 0.9767 |
| 11727691_a_at | IL11 | 6,30 | 6,53 | 0,23 | 0.4267 | 0.7572 | 7,11 | 6,73 | -0,38 | 0.2194 | 0.9676 |
| 11751797_a_at | IL11 | 3,27 | 3,28 | 0,01 | 0.9730 | 0.9926 | 3,58 | 3,34 | -0,24 | 0.2290 | 0.9676 |
| 11721024_a_at | IL11RA | 6,42 | 7,43 | 1,01 | **0.0323** | 0.2427 | 7,10 | 7,26 | 0,16 | 0.7047 | 0.9868 |
| 11737464_a_at | IL11RA | 6,29 | 6,80 | 0,51 | 0.3046 | 0.6679 | 6,65 | 6,98 | 0,33 | 0.2348 | 0.9676 |
| 11737465_at | IL11RA | 6,24 | 7,18 | 0,94 | 0.0664 | 0.3467 | 6,38 | 7,12 | 0,74 | 0.1393 | 0.9534 |
| 11756769_a_at | IL12A | 7,69 | 5,92 | -1,77 | **0.0103** | 0.1326 | 7,67 | 7,74 | 0,07 | 0.8494 | 0.9930 |
| 11737833_at | IL12B | 4,99 | 3,68 | -1,31 | 0.1535 | 0.5055 | 3,47 | 3,59 | 0,12 | 0.8352 | 0.9925 |
| 11737834_at | IL12B | 5,94 | 5,83 | -0,12 | 0.5494 | 0.8299 | 6,56 | 6,55 | -0,01 | 0.9646 | 0.9987 |
| 11741270_x_at | IL12RB1 | 7,23 | 7,45 | 0,22 | 0.5009 | 0.8021 | 7,22 | 7,70 | 0,48 | 0.1179 | 0.9432 |
| 11752440_a_at | IL12RB1 | 5,45 | 5,90 | 0,45 | 0.3334 | 0.6896 | 5,70 | 5,82 | 0,12 | 0.7074 | 0.9868 |
| 11759434_at | IL12RB1 | 5,87 | 6,88 | 1,01 | **0.0079** | 0.1139 | 6,65 | 7,01 | 0,35 | 0.3655 | 0.9689 |
| 11737427_at | IL12RB2 | 3,62 | 5,86 | 2,24 | **0.0023** | 0.0549 | 4,37 | 4,41 | 0,04 | 0.8802 | 0.9955 |
| 11748377_a_at | IL12RB2 | 5,13 | 6,46 | 1,33 | **0.0052** | 0.0881 | 5,07 | 5,00 | -0,08 | 0.8155 | 0.9925 |
| 11749285_a_at | IL12RB2 | 3,00 | 4,84 | 1,84 | **0.0065** | 0.1021 | 3,46 | 3,43 | -0,04 | 0.8489 | 0.9930 |
| 11755863_a_at | IL12RB2 | 3,72 | 5,58 | 1,86 | **0.0047** | 0.0846 | 4,21 | 4,38 | 0,18 | 0.6510 | 0.9810 |
| 11735860_at | IL13 | 4,52 | 4,62 | 0,10 | 0.5766 | 0.8433 | 5,07 | 4,94 | -0,14 | 0.6770 | 0.9836 |
| 11745622_a_at | IL13 | 5,44 | 5,30 | -0,14 | 0.5839 | 0.8473 | 5,60 | 5,19 | -0,41 | 0.2098 | 0.9676 |
| 11727903_a_at | IL13RA1 | 6,05 | 6,41 | 0,36 | 0.4788 | 0.7894 | 4,45 | 5,26 | 0,81 | 0.1065 | 0.9405 |
| 11727904_s_at | IL13RA1 | 7,34 | 7,64 | 0,30 | 0.4119 | 0.7464 | 6,02 | 6,85 | 0,83 | 0.2516 | 0.9676 |
| 11727905_a_at | IL13RA1 | 8,79 | 8,46 | -0,33 | 0.4503 | 0.7727 | 6,20 | 7,40 | 1,20 | 0.0617 | 0.8989 |
| 11750299_a_at | IL13RA1 | 7,90 | 7,94 | 0,04 | 0.9213 | 0.9777 | 4,52 | 6,73 | 2,21 | 0.0509 | 0.8765 |
| 11757033_a_at | IL13RA2 | 2,49 | 2,64 | 0,15 | 0.4173 | 0.7502 | 3,01 | 3,00 | -0,01 | 0.9784 | 0.9987 |
| 11725255_a_at | IL15 | 6,26 | 7,11 | 0,85 | 0.3229 | 0.6819 | 6,52 | 6,59 | 0,06 | 0.9214 | 0.9977 |
| 11740881_x_at | IL15 | 5,60 | 6,34 | 0,74 | 0.4133 | 0.7479 | 5,67 | 5,66 | 0,00 | 0.9978 | 0.9997 |
| 11759832_a_at | IL15 | 4,23 | 4,45 | 0,22 | 0.6834 | 0.8933 | 4,35 | 4,37 | 0,03 | 0.9671 | 0.9987 |
| 11731039_s_at | IL15RA | 7,39 | 6,77 | -0,62 | 0.0618 | 0.3341 | 7,73 | 7,72 | -0,02 | 0.9685 | 0.9987 |
| 11732019_a_at | IL15RA | 8,34 | 7,79 | -0,55 | 0.1049 | 0.4279 | 9,26 | 8,76 | -0,50 | 0.2449 | 0.9676 |
| 11748229_x_at | IL15RA | 6,70 | 6,72 | 0,02 | 0.9300 | 0.9802 | 7,75 | 7,34 | -0,41 | 0.2623 | 0.9676 |
| 11734727_a_at | IL16 | 10,62 | 10,50 | -0,12 | 0.6948 | 0.8978 | 10,00 | 10,27 | 0,28 | 0.4344 | 0.9689 |
| 11741610_a_at | IL16 | 9,52 | 8,98 | -0,54 | 0.1961 | 0.5568 | 7,81 | 8,41 | 0,59 | 0.1247 | 0.9432 |
| 11746376_a_at | IL16 | 10,11 | 10,00 | -0,11 | 0.7219 | 0.9095 | 9,66 | 10,07 | 0,41 | 0.3551 | 0.9689 |
| 11748089_a_at | IL16 | 5,49 | 5,31 | -0,19 | 0.4932 | 0.7977 | 5,64 | 5,81 | 0,17 | 0.4014 | 0.9689 |
| 11755752_a_at | IL16 | 8,63 | 8,67 | 0,04 | 0.9300 | 0.9802 | 7,90 | 8,43 | 0,53 | 0.0976 | 0.9321 |
| 11758090_s_at | IL16 | 7,87 | 7,45 | -0,42 | 0.3618 | 0.7111 | 7,34 | 7,77 | 0,43 | 0.4521 | 0.9689 |
| 11760386_at | IL16 | 7,70 | 7,84 | 0,14 | 0.8880 | 0.9678 | 7,44 | 7,91 | 0,47 | 0.3639 | 0.9689 |
| 11760387_at | IL16 | 8,99 | 9,40 | 0,41 | 0.1415 | 0.4872 | 8,23 | 8,74 | 0,52 | 0.1438 | 0.9536 |
| 11732112_at | IL17A | 3,18 | 3,23 | 0,05 | 0.8650 | 0.9604 | 3,22 | 2,94 | -0,28 | 0.4709 | 0.9689 |
| 11732113_at | IL17A | 3,19 | 3,46 | 0,27 | 0.2231 | 0.5869 | 3,61 | 3,43 | -0,18 | 0.6003 | 0.9767 |
| 11725582_at | IL17B | 4,93 | 4,98 | 0,06 | 0.8016 | 0.9388 | 5,22 | 4,86 | -0,36 | 0.2001 | 0.9676 |
| 11741836_at | IL17C | 5,81 | 5,99 | 0,18 | 0.5076 | 0.8061 | 6,10 | 5,87 | -0,23 | 0.4708 | 0.9689 |
| 11720693_a_at | IL17D | 4,70 | 5,35 | 0,65 | 0.1594 | 0.5124 | 5,64 | 5,55 | -0,09 | 0.7622 | 0.9904 |
| 11738223_a_at | IL17F | 3,77 | 3,50 | -0,26 | 0.1776 | 0.5354 | 4,19 | 3,91 | -0,28 | 0.4368 | 0.9689 |
| 11740065_at | IL17RA | 6,54 | 7,85 | 1,30 | 0.0888 | 0.3968 | 6,09 | 6,90 | 0,81 | 0.2558 | 0.9676 |
| 11723085_s_at | IL17RB | 5,18 | 5,49 | 0,31 | 0.5893 | 0.8498 | 5,24 | 5,73 | 0,50 | 0.5036 | 0.9698 |
| 11726404_x_at | IL17RC | 7,78 | 7,92 | 0,14 | 0.5491 | 0.8296 | 7,64 | 7,60 | -0,03 | 0.8616 | 0.9934 |
| 11736502_a_at | IL17RC | 4,51 | 4,49 | -0,02 | 0.9047 | 0.9726 | 4,38 | 4,06 | -0,31 | 0.1253 | 0.9432 |
| 11736503_x_at | IL17RC | 3,88 | 3,71 | -0,17 | 0.3915 | 0.7339 | 4,13 | 3,96 | -0,17 | 0.5519 | 0.9724 |
| 11746244_a_at | IL17RC | 2,85 | 3,28 | 0,43 | 0.1134 | 0.4429 | 3,24 | 2,88 | -0,36 | 0.3155 | 0.9689 |
| 11746245_x_at | IL17RC | 4,42 | 4,55 | 0,12 | 0.5996 | 0.8548 | 5,31 | 5,15 | -0,16 | 0.6704 | 0.9826 |
| 11748446_a_at | IL17RC | 4,80 | 5,02 | 0,22 | 0.4012 | 0.7402 | 5,67 | 5,17 | -0,49 | 0.1162 | 0.9432 |
| 11748447_x_at | IL17RC | 4,71 | 4,75 | 0,04 | 0.8534 | 0.9564 | 5,25 | 5,03 | -0,22 | 0.4821 | 0.9689 |
| 11723171_a_at | IL17RD | 2,71 | 2,51 | -0,20 | 0.2613 | 0.6280 | 2,82 | 2,84 | 0,01 | 0.9702 | 0.9987 |
| 11723172_a_at | IL17RD | 2,72 | 2,60 | -0,12 | 0.5984 | 0.8542 | 2,77 | 2,48 | -0,29 | 0.4900 | 0.9689 |
| 11723173_at | IL17RD | 3,00 | 2,83 | -0,18 | 0.2717 | 0.6375 | 4,42 | 3,31 | -1,12 | 0.1182 | 0.9432 |
| 11750390_a_at | IL17RD | 5,25 | 5,10 | -0,15 | 0.4497 | 0.7723 | 5,47 | 5,09 | -0,38 | 0.1579 | 0.9573 |
| 11733493_a_at | IL17RE | 7,01 | 6,81 | -0,20 | 0.2676 | 0.6338 | 7,05 | 7,08 | 0,03 | 0.8711 | 0.9939 |
| 11750391_a_at | IL17RE | 4,37 | 4,46 | 0,09 | 0.6577 | 0.8823 | 5,29 | 5,17 | -0,12 | 0.5586 | 0.9736 |
| 11750392_a_at | IL17RE | 7,01 | 7,08 | 0,07 | 0.7320 | 0.9136 | 7,18 | 6,96 | -0,22 | 0.3744 | 0.9689 |
| 11753377_a_at | IL17RE | 4,45 | 4,60 | 0,16 | 0.4499 | 0.7724 | 4,82 | 4,62 | -0,20 | 0.6415 | 0.9806 |
| 11762585_at | IL17RE | 3,72 | 3,59 | -0,12 | 0.5393 | 0.8244 | 3,72 | 3,78 | 0,06 | 0.7972 | 0.9920 |
| 11762586_x_at | IL17RE | 4,05 | 3,94 | -0,11 | 0.6058 | 0.8574 | 4,74 | 4,76 | 0,02 | 0.9345 | 0.9978 |
| 11738330_at | IL17REL | 5,14 | 4,89 | -0,26 | 0.1874 | 0.5474 | 5,34 | 5,18 | -0,16 | 0.5580 | 0.9734 |
| 11727092_x_at | IL18 | 3,72 | 5,46 | 1,74 | **0.0021** | 0.0520 | 3,88 | 4,13 | 0,25 | 0.5267 | 0.9698 |
| 11726928_s_at | IL18BP | 6,57 | 6,71 | 0,14 | 0.6656 | 0.8864 | 6,75 | 7,18 | 0,43 | 0.3103 | 0.9689 |
| 11762407_at | IL18BP | 7,64 | 8,29 | 0,65 | 0.1588 | 0.5115 | 7,89 | 8,31 | 0,42 | 0.1346 | 0.9479 |
| 11735275_at | IL18R1 | 5,34 | 6,73 | 1,39 | 0.0974 | 0.4136 | 4,08 | 4,02 | -0,06 | 0.8682 | 0.9934 |
| 11735276_a_at | IL18R1 | 2,91 | 5,17 | 2,26 | **0.0064** | 0.1013 | 3,16 | 3,03 | -0,13 | 0.7442 | 0.9886 |
| 11732017_a_at | IL18RAP | 4,35 | 8,11 | 3,76 | **0.0000** | **0.0037** | 4,18 | 4,08 | -0,10 | 0.5306 | 0.9700 |
| 11749598_a_at | IL18RAP | 3,26 | 7,68 | 4,42 | **0.0000** | **0.0003** | 2,91 | 2,79 | -0,12 | 0.5915 | 0.9767 |
| 11731352_a_at | IL19 | 5,29 | 5,17 | -0,12 | 0.4954 | 0.7989 | 5,56 | 5,30 | -0,26 | 0.3742 | 0.9689 |
| 11762944_at | IL19 | 3,28 | 3,38 | 0,10 | 0.5727 | 0.8416 | 3,49 | 3,09 | -0,40 | 0.4044 | 0.9689 |
| 11725198_at | IL1A | 3,89 | 3,19 | -0,70 | 0.1477 | 0.4976 | 3,42 | 3,99 | 0,56 | 0.2608 | 0.9676 |
| 11719916_at | IL1B | 4,33 | 8,06 | 3,73 | **0.0001** | **0.0057** | 4,70 | 4,82 | 0,13 | 0.8402 | 0.9925 |
| 11735897_a_at | IL1F10 | 3,53 | 3,25 | -0,28 | 0.1433 | 0.4904 | 3,54 | 3,14 | -0,41 | 0.1075 | 0.9414 |
| 11718025_at | IL1R1 | 2,69 | 3,26 | 0,58 | 0.1237 | 0.4603 | 2,60 | 2,76 | 0,16 | 0.5949 | 0.9767 |
| 11756953_a_at | IL1R1 | 5,45 | 5,57 | 0,12 | 0.5752 | 0.8424 | 4,15 | 4,29 | 0,14 | 0.6398 | 0.9804 |
| 11728500_a_at | IL1R2 | 3,53 | 5,56 | 2,04 | **0.0008** | **0.0289** | 3,72 | 3,62 | -0,10 | 0.7254 | 0.9874 |
| 11729098_a_at | IL1RAP | 5,65 | 6,29 | 0,65 | 0.1689 | 0.5247 | 5,81 | 5,86 | 0,06 | 0.7745 | 0.9904 |
| 11729099_a_at | IL1RAP | 5,06 | 6,91 | 1,85 | **0.0124** | 0.1462 | 5,76 | 5,81 | 0,05 | 0.8961 | 0.9970 |
| 11732585_a_at | IL1RAP | 4,42 | 4,26 | -0,16 | 0.7642 | 0.9261 | 4,96 | 5,00 | 0,05 | 0.9238 | 0.9978 |
| 11761995_a_at | IL1RAP | 3,51 | 3,35 | -0,16 | 0.4584 | 0.7772 | 3,07 | 2,99 | -0,08 | 0.7793 | 0.9907 |
| 11737250_at | IL1RAPL1 | 5,95 | 5,87 | -0,08 | 0.7156 | 0.9069 | 6,00 | 6,05 | 0,04 | 0.8757 | 0.9946 |
| 11751140_a_at | IL1RAPL2 | 2,80 | 2,85 | 0,06 | 0.7669 | 0.9271 | 2,73 | 2,76 | 0,03 | 0.8721 | 0.9943 |
| 11734884_a_at | IL1RL1 | 4,65 | 5,13 | 0,49 | 0.0903 | 0.4000 | 5,30 | 4,90 | -0,41 | 0.2354 | 0.9676 |
| 11734885_at | IL1RL1 | 3,68 | 4,00 | 0,32 | 0.1720 | 0.5286 | 4,50 | 3,90 | -0,60 | 0.0672 | 0.9103 |
| 11738178_at | IL1RL1 | 4,51 | 5,03 | 0,52 | 0.0964 | 0.4115 | 4,53 | 4,55 | 0,02 | 0.9449 | 0.9979 |
| 11745596_a_at | IL1RL1 | 4,65 | 5,13 | 0,49 | 0.0903 | 0.4000 | 5,30 | 4,90 | -0,41 | 0.2354 | 0.9676 |
| 11759145_s_at | IL1RL1 | 4,46 | 4,90 | 0,44 | 0.0985 | 0.4159 | 4,44 | 4,44 | 0,00 | 0.9975 | 0.9997 |
| 11735830_a_at | IL1RL2 | 4,93 | 4,78 | -0,15 | 0.3319 | 0.6885 | 5,09 | 4,79 | -0,29 | 0.3149 | 0.9689 |
| 11719754_s_at | IL1RN | 4,06 | 6,91 | 2,85 | **0.0000** | **0.0004** | 4,52 | 4,48 | -0,04 | 0.8430 | 0.9930 |
| 11740722_a_at | IL1RN | 4,75 | 5,53 | 0,78 | **0.0005** | **0.0195** | 4,89 | 4,88 | -0,01 | 0.9600 | 0.9982 |
| 11733943_at | IL2 | 2,79 | 2,85 | 0,07 | 0.6938 | 0.8975 | 2,87 | 2,71 | -0,16 | 0.4782 | 0.9689 |
| 11731392_at | IL20 | 3,09 | 3,03 | -0,07 | 0.7600 | 0.9245 | 3,09 | 3,21 | 0,12 | 0.5174 | 0.9698 |
| 11753698_a_at | IL20 | 3,00 | 3,04 | 0,03 | 0.8802 | 0.9652 | 3,36 | 3,10 | -0,26 | 0.3842 | 0.9689 |
| 11728363_s_at | IL20RA | 2,59 | 2,56 | -0,03 | 0.8765 | 0.9638 | 2,67 | 2,64 | -0,03 | 0.8829 | 0.9955 |
| 11728364_a_at | IL20RA | 3,90 | 3,79 | -0,11 | 0.5320 | 0.8201 | 4,06 | 3,83 | -0,22 | 0.4915 | 0.9689 |
| 11727589_a_at | IL20RB | 4,19 | 4,27 | 0,08 | 0.7686 | 0.9277 | 4,38 | 4,08 | -0,30 | 0.2375 | 0.9676 |
| 11727590_a_at | IL20RB | 4,88 | 5,30 | 0,42 | 0.2561 | 0.6226 | 6,18 | 5,97 | -0,21 | 0.4608 | 0.9689 |
| 11753604_a_at | IL21 | 4,18 | 4,07 | -0,11 | 0.5267 | 0.8168 | 4,08 | 3,95 | -0,12 | 0.5184 | 0.9698 |
| 11739739_a_at | IL21R | 6,20 | 9,17 | 2,97 | **0.0009** | **0.0313** | 6,10 | 6,71 | 0,61 | 0.0512 | 0.8765 |
| 11739740_a_at | IL21R | 5,01 | 6,52 | 1,51 | **0.0017** | **0.0460** | 4,28 | 4,33 | 0,05 | 0.8405 | 0.9925 |
| 11733737_at | IL22 | 4,30 | 4,39 | 0,09 | 0.7958 | 0.9372 | 5,11 | 4,39 | -0,72 | 0.0676 | 0.9119 |
| 11752600_a_at | IL22RA1 | 5,94 | 6,04 | 0,10 | 0.6426 | 0.8758 | 6,38 | 6,06 | -0,32 | 0.3154 | 0.9689 |
| 11755853_a_at | IL22RA1 | 4,24 | 4,16 | -0,08 | 0.7288 | 0.9125 | 4,62 | 4,40 | -0,22 | 0.5494 | 0.9718 |
| 11763791_a_at | IL22RA1 | 4,38 | 4,29 | -0,09 | 0.7968 | 0.9375 | 4,71 | 4,39 | -0,32 | 0.1743 | 0.9672 |
| 11731374_a_at | IL22RA2 | 3,60 | 3,23 | -0,37 | 0.2156 | 0.5790 | 4,00 | 3,22 | -0,78 | 0.3886 | 0.9689 |
| 11738383_a_at | IL22RA2 | 2,65 | 2,66 | 0,01 | 0.9559 | 0.9875 | 2,96 | 3,10 | 0,14 | 0.6059 | 0.9767 |
| 11732301_at | IL23A | 5,99 | 4,96 | -1,03 | **0.0452** | 0.2863 | 5,82 | 6,57 | 0,75 | 0.0623 | 0.9009 |
| 11761418_x_at | IL23A | 11,88 | 12,18 | 0,30 | 0.5368 | 0.8232 | 11,92 | 12,39 | 0,46 | 0.0518 | 0.8789 |
| 11761525_a_at | IL23A | 4,44 | 5,47 | 1,03 | **0.0047** | 0.0842 | 3,87 | 3,50 | -0,37 | 0.3643 | 0.9689 |
| 11762318_x_at | IL23A | 10,44 | 11,83 | 1,39 | 0.1627 | 0.5162 | 11,10 | 11,52 | 0,42 | **0.0487** | 0.8697 |
| 11737362_x_at | IL23R | 2,68 | 2,61 | -0,07 | 0.6935 | 0.8974 | 2,54 | 2,83 | 0,29 | 0.2755 | 0.9676 |
| 11748794_a_at | IL23R | 2,70 | 2,73 | 0,02 | 0.8770 | 0.9640 | 2,48 | 2,65 | 0,17 | 0.3557 | 0.9689 |
| 11731834_a_at | IL24 | 10,59 | 10,27 | -0,32 | 0.1932 | 0.5543 | 9,37 | 10,46 | 1,09 | **0.0126** | 0.6665 |
| 11741775_a_at | IL24 | 10,11 | 9,54 | -0,57 | **0.0306** | 0.2363 | 9,04 | 10,05 | 1,01 | **0.0144** | 0.6749 |
| 11754176_a_at | IL24 | 5,99 | 6,07 | 0,08 | 0.8617 | 0.9595 | 6,70 | 6,53 | -0,17 | 0.5468 | 0.9715 |
| 11738490_a_at | IL25 | 5,41 | 5,09 | -0,32 | 0.1965 | 0.5575 | 5,93 | 5,79 | -0,14 | 0.6143 | 0.9783 |
| 11741934_a_at | IL25 | 5,67 | 5,82 | 0,14 | 0.4439 | 0.7687 | 5,85 | 5,52 | -0,33 | 0.2070 | 0.9676 |
| 11737150_at | IL26 | 5,24 | 4,11 | -1,13 | **0.0300** | 0.2344 | 6,48 | 5,63 | -0,85 | 0.1453 | 0.9538 |
| 11735577_at | IL27 | 4,50 | 4,62 | 0,12 | 0.5767 | 0.8434 | 5,47 | 5,33 | -0,13 | 0.4964 | 0.9694 |
| 11729821_at | IL27RA | 8,11 | 7,57 | -0,54 | 0.1517 | 0.5026 | 7,14 | 7,59 | 0,45 | 0.0653 | 0.9085 |
| 11739954_at | IL2RA | 6,89 | 5,20 | -1,70 | **0.0413** | 0.2743 | 4,89 | 5,07 | 0,19 | 0.8531 | 0.9931 |
| 11739955_a_at | IL2RA | 8,32 | 6,97 | -1,35 | **0.0210** | 0.1940 | 6,44 | 7,35 | 0,91 | 0.0563 | 0.8829 |
| 11752775_a_at | IL2RA | 6,76 | 5,80 | -0,95 | **0.0142** | 0.1572 | 5,90 | 6,18 | 0,28 | 0.3982 | 0.9689 |
| 11722635_at | IL2RB | 6,70 | 10,94 | 4,24 | **0.0000** | **0.0020** | 6,31 | 7,21 | 0,89 | 0.1397 | 0.9535 |
| 11720207_a_at | IL2RG | 11,07 | 11,12 | 0,05 | 0.8132 | 0.9432 | 10,93 | 11,15 | 0,22 | 0.2710 | 0.9676 |
| 11753814_a_at | IL2RG | 9,96 | 11,01 | 1,05 | 0.1733 | 0.5297 | 10,29 | 10,63 | 0,35 | 0.0904 | 0.9260 |
| 11753882_a_at | IL2RG | 10,92 | 11,53 | 0,61 | 0.2604 | 0.6272 | 10,96 | 11,40 | 0,44 | 0.0569 | 0.8839 |
| 11753883_x_at | IL2RG | 10,99 | 11,59 | 0,59 | 0.2312 | 0.5957 | 11,18 | 11,55 | 0,37 | 0.0797 | 0.9225 |
| 11754181_a_at | IL2RG | 11,25 | 11,67 | 0,42 | 0.1912 | 0.5519 | 11,19 | 11,38 | 0,19 | 0.3592 | 0.9689 |
| 11754182_x_at | IL2RG | 11,72 | 12,13 | 0,41 | 0.1851 | 0.5446 | 11,87 | 12,09 | 0,22 | 0.3159 | 0.9689 |
| 11732963_at | IL3 | 3,79 | 3,60 | -0,19 | 0.5072 | 0.8058 | 4,38 | 4,26 | -0,12 | 0.5647 | 0.9742 |
| 11738590_at | IL31 | 3,51 | 3,57 | 0,05 | 0.8336 | 0.9496 | 4,16 | 3,62 | -0,54 | 0.2335 | 0.9676 |
| 11738528_a_at | IL31RA | 4,33 | 4,15 | -0,18 | 0.3260 | 0.6848 | 4,52 | 4,32 | -0,20 | 0.6661 | 0.9819 |
| 11750799_x_at | IL31RA | 3,48 | 3,16 | -0,32 | 0.1020 | 0.4224 | 3,49 | 3,29 | -0,19 | 0.2857 | 0.9681 |
| 11753023_x_at | IL31RA | 3,85 | 3,72 | -0,12 | 0.4669 | 0.7830 | 4,42 | 4,28 | -0,15 | 0.6444 | 0.9807 |
| 11762511_a_at | IL31RA | 4,38 | 4,25 | -0,13 | 0.5384 | 0.8238 | 4,06 | 3,93 | -0,13 | 0.7978 | 0.9922 |
| 11762812_at | IL31RA | 3,07 | 2,88 | -0,19 | 0.5312 | 0.8198 | 2,71 | 2,71 | 0,01 | 0.9804 | 0.9988 |
| 11763997_a_at | IL31RA | 3,86 | 3,80 | -0,06 | 0.7486 | 0.9207 | 4,44 | 4,02 | -0,42 | 0.2389 | 0.9676 |
| 11734890_a_at | IL32 | 4,15 | 8,99 | 4,85 | **0.0000** | **0.0002** | 4,58 | 4,75 | 0,17 | 0.5756 | 0.9758 |
| 11735174_a_at | IL32 | 4,08 | 8,14 | 4,06 | **0.0000** | **0.0001** | 4,00 | 4,17 | 0,17 | 0.6444 | 0.9807 |
| 11736394_x_at | IL32 | 4,18 | 7,57 | 3,39 | **0.0000** | **0.0004** | 4,19 | 4,15 | -0,04 | 0.9154 | 0.9977 |
| 11753515_a_at | IL32 | 4,24 | 8,99 | 4,75 | **0.0000** | **0.0002** | 4,59 | 4,80 | 0,21 | 0.4986 | 0.9695 |
| 11743858_at | IL33 | 2,53 | 2,51 | -0,02 | 0.9162 | 0.9764 | 2,64 | 2,69 | 0,05 | 0.7987 | 0.9925 |
| 11743859_x_at | IL33 | 2,57 | 2,77 | 0,20 | 0.2235 | 0.5873 | 3,04 | 2,77 | -0,28 | 0.2721 | 0.9676 |
| 11751426_a_at | IL33 | 3,04 | 2,81 | -0,24 | 0.1940 | 0.5549 | 2,77 | 2,68 | -0,10 | 0.6069 | 0.9770 |
| 11726706_at | IL34 | 4,81 | 5,01 | 0,20 | 0.4582 | 0.7771 | 5,17 | 4,89 | -0,28 | 0.4501 | 0.9689 |
| 11726707_x_at | IL34 | 5,12 | 5,09 | -0,03 | 0.9232 | 0.9782 | 5,59 | 5,29 | -0,30 | 0.3961 | 0.9689 |
| 11734628_at | IL36A | 3,95 | 3,68 | -0,27 | 0.1164 | 0.4482 | 4,38 | 4,38 | 0,00 | 0.9870 | 0.9997 |
| 11737490_a_at | IL36B | 4,60 | 4,57 | -0,03 | 0.8545 | 0.9569 | 4,83 | 4,69 | -0,14 | 0.6747 | 0.9834 |
| 11738901_at | IL36B | 4,36 | 4,03 | -0,33 | 0.1812 | 0.5397 | 4,17 | 4,08 | -0,09 | 0.6141 | 0.9783 |
| 11753008_a_at | IL36G | 2,72 | 2,60 | -0,12 | 0.5193 | 0.8129 | 3,04 | 2,93 | -0,10 | 0.5707 | 0.9753 |
| 11732733_a_at | IL36RN | 3,09 | 3,22 | 0,13 | 0.6139 | 0.8619 | 3,81 | 3,71 | -0,10 | 0.7479 | 0.9889 |
| 11732734_a_at | IL36RN | 2,86 | 2,69 | -0,16 | 0.3688 | 0.7170 | 2,87 | 2,72 | -0,15 | 0.4189 | 0.9689 |
| 11735417_a_at | IL36RN | 4,84 | 4,92 | 0,09 | 0.6980 | 0.8994 | 4,73 | 4,76 | 0,03 | 0.8939 | 0.9970 |
| 11732837_a_at | IL37 | 3,48 | 3,64 | 0,16 | 0.5461 | 0.8282 | 3,89 | 4,03 | 0,14 | 0.5907 | 0.9767 |
| 11732469_at | IL3RA | 7,03 | 7,95 | 0,93 | 0.0549 | 0.3158 | 7,31 | 7,49 | 0,18 | 0.5713 | 0.9753 |
| 11735020_a_at | IL4 | 5,55 | 5,77 | 0,22 | 0.4049 | 0.7424 | 5,87 | 5,59 | -0,29 | 0.2859 | 0.9681 |
| 11732110_a_at | IL4I1 | 5,74 | 5,36 | -0,38 | 0.0564 | 0.3196 | 5,54 | 5,42 | -0,13 | 0.5784 | 0.9764 |
| 11718073_at | IL4R | 9,76 | 11,05 | 1,29 | **0.0451** | 0.2859 | 7,98 | 9,74 | 1,76 | **0.0002** | 0.2604 |
| 11718074_a_at | IL4R | 5,87 | 5,68 | -0,19 | 0.8137 | 0.9433 | 3,95 | 4,20 | 0,25 | 0.4275 | 0.9689 |
| 11718075_at | IL4R | 7,49 | 8,28 | 0,79 | 0.1777 | 0.5355 | 6,38 | 7,55 | 1,17 | **0.0117** | 0.6665 |
| 11742511_a_at | IL4R | 8,90 | 10,57 | 1,67 | **0.0129** | 0.1492 | 7,71 | 9,41 | 1,70 | **0.0013** | 0.4030 |
| 11748724_a_at | IL4R | 5,22 | 5,95 | 0,73 | 0.1191 | 0.4526 | 4,20 | 4,34 | 0,14 | 0.6116 | 0.9780 |
| 11738281_a_at | IL5 | 4,23 | 5,33 | 1,10 | **0.0108** | 0.1363 | 5,04 | 5,38 | 0,34 | 0.5983 | 0.9767 |
| 11734541_a_at | IL5RA | 4,94 | 4,03 | -0,91 | 0.1010 | 0.4208 | 4,98 | 5,32 | 0,35 | 0.5836 | 0.9767 |
| 11738838_a_at | IL5RA | 2,98 | 2,83 | -0,15 | 0.8308 | 0.9487 | 3,66 | 3,46 | -0,20 | 0.6947 | 0.9854 |
| 11740816_a_at | IL5RA | 3,33 | 3,14 | -0,19 | 0.6575 | 0.8823 | 3,86 | 3,96 | 0,10 | 0.8221 | 0.9925 |
| 11753566_a_at | IL5RA | 5,79 | 4,62 | -1,17 | **0.0262** | 0.2182 | 6,09 | 7,13 | 1,04 | 0.1236 | 0.9432 |
| 11746463_a_at | IL6 | 5,34 | 5,03 | -0,31 | 0.7151 | 0.9067 | 4,48 | 5,86 | 1,38 | 0.0512 | 0.8765 |
| 11760425_a_at | IL6 | 4,06 | 4,15 | 0,09 | 0.6844 | 0.8938 | 4,41 | 3,88 | -0,53 | 0.4003 | 0.9689 |
| 11736509_x_at | IL6R | 8,53 | 7,13 | -1,41 | **0.0436** | 0.2810 | 9,93 | 8,90 | -1,03 | **0.0151** | 0.6825 |
| 11736510_a_at | IL6R | 8,29 | 6,09 | -2,20 | **0.0044** | 0.0812 | 7,39 | 6,91 | -0,48 | 0.1929 | 0.9676 |
| 11741958_a_at | IL6R | 7,98 | 6,35 | -1,63 | **0.0399** | 0.2696 | 8,05 | 7,59 | -0,47 | 0.1700 | 0.9660 |
| 11741959_x_at | IL6R | 9,40 | 8,36 | -1,04 | **0.0254** | 0.2146 | 9,04 | 8,11 | -0,93 | 0.0931 | 0.9298 |
| 11730757_a_at | IL6ST | 7,54 | 6,75 | -0,79 | **0.2992** | 0.6630 | 7,25 | 6,64 | -0,61 | 0.1749 | 0.9672 |
| 11730758_a_at | IL6ST | 6,37 | 6,70 | 0,33 | 0.5174 | 0.8117 | 7,69 | 7,00 | -0,69 | **0.0333** | 0.8135 |
| 11753579_a_at | IL6ST | 7,93 | 8,61 | 0,67 | 0.2884 | 0.6534 | 8,71 | 8,08 | -0,63 | 0.3214 | 0.9689 |
| 11753878_s_at | IL6ST | 7,51 | 7,34 | -0,17 | 0.8401 | 0.9515 | 8,35 | 7,44 | -0,91 | 0.1688 | 0.9660 |
| 11753879_x_at | IL6ST | 7,95 | 7,43 | -0,51 | 0.2273 | 0.5919 | 8,57 | 7,50 | -1,07 | 0.0899 | 0.9260 |
| 11753886_a_at | IL6ST | 7,36 | 8,28 | 0,92 | 0.2281 | 0.5926 | 9,08 | 8,23 | -0,85 | 0.2312 | 0.9676 |
| 11759037_s_at | IL6ST | 7,29 | 6,61 | -0,68 | 0.3172 | 0.6770 | 8,10 | 6,48 | -1,62 | **0.0011** | 0.3932 |
| 11759038_at | IL6ST | 7,82 | 7,64 | -0,17 | 0.8324 | 0.9493 | 8,74 | 7,83 | -0,91 | 0.0675 | 0.9118 |
| 11735257_a_at | IL7 | 7,34 | 4,55 | -2,80 | **0.0002** | **0.0097** | 6,89 | 6,34 | -0,55 | 0.4017 | 0.9689 |
| 11735258_x_at | IL7 | 7,00 | 4,97 | -2,02 | **0.0039** | 0.0760 | 7,26 | 6,84 | -0,42 | 0.5541 | 0.9729 |
| 11747034_a_at | IL7 | 8,38 | 6,11 | -2,28 | **0.0010** | **0.0325** | 7,28 | 7,20 | -0,08 | 0.8704 | 0.9937 |
| 11747035_a_at | IL7 | 6,69 | 6,49 | -0,21 | 0.7043 | 0.9023 | 7,06 | 8,10 | 1,05 | **0.0407** | 0.8449 |
| 11754162_a_at | IL7 | 6,53 | 3,97 | -2,56 | **0.0012** | **0.0365** | 6,58 | 6,05 | -0,53 | 0.4944 | 0.9691 |
| 11754163_x_at | IL7 | 6,61 | 4,80 | -1,81 | **0.0209** | 0.1936 | 6,82 | 7,07 | 0,25 | 0.4531 | 0.9689 |
| 11733187_a_at | IL7R | 4,30 | 3,69 | -0,61 | **0.0108** | 0.1363 | 3,96 | 3,85 | -0,11 | 0.6370 | 0.9801 |
| 11751647_a_at | IL7R | 3,10 | 3,26 | 0,16 | 0.5770 | 0.8435 | 3,29 | 3,60 | 0,31 | 0.2334 | 0.9676 |
| 11762266_x_at | IL7R | 3,38 | 3,14 | -0,25 | 0.2601 | 0.6268 | 3,35 | 3,38 | 0,03 | 0.8947 | 0.9970 |
| 11718841_s_at | IL8 | 4,51 | 9,52 | 5,01 | **0.0000** | **0.0001** | 4,49 | 5,36 | 0,87 | 0.2835 | 0.9678 |
| 11754026_a_at | IL8 | 4,18 | 8,80 | 4,62 | **0.0000** | **0.0000** | 4,70 | 5,09 | 0,39 | 0.3378 | 0.9689 |
| 11763226_x_at | IL8 | 4,69 | 8,36 | 3,68 | **0.0000** | **0.0003** | 5,24 | 5,09 | -0,16 | 0.7192 | 0.9872 |
| 11731785_at | IL9 | 3,10 | 3,12 | 0,02 | 0.9396 | 0.9829 | 3,28 | 3,09 | -0,19 | 0.6529 | 0.9810 |
| 11741876_a_at | IL9R | 6,21 | 5,96 | -0,24 | 0.4270 | 0.7575 | 6,44 | 6,31 | -0,13 | 0.6829 | 0.9840 |
| 11741877_s_at | IL9R | 5,10 | 5,21 | 0,11 | 0.5936 | 0.8521 | 5,52 | 5,33 | -0,18 | 0.6322 | 0.9801 |
| 11748692_a_at | IL9R | 5,53 | 5,76 | 0,24 | 0.3236 | 0.6826 | 6,34 | 5,80 | -0,54 | 0.2452 | 0.9676 |
|  |  |  |  |  |  |  |  |  |  |  |  |
|  |  |  |  |  |  |  |  |  |  |  |  |
| 11761777_x_at | ITGAX | 5,88 | 7,70 | 1,8206 | **0,0100** | 0,1303 | 7,24 | 8,34 | 1,1002 | **0,0085** | 0,6244 |
| 11763787_a_at | ITGAX | 5,01 | 7,80 | 2,7881 | **0,0013** | **0,0387** | 5,89 | 7,34 | 1,4540 | **0,0152** | 0,6855 |
| 11743475_a_at | ITGAX | 8,00 | 10,94 | 2,9326 | **0,0037** | 0,0736 | 9,16 | 10,92 | 1,7549 | **0,0299** | 0,7987 |
| 11750033_a_at | ITGAX | 6,31 | 8,76 | 2,4500 | **0,0044** | 0,0816 | 7,33 | 8,82 | 1,4845 | **0,0154** | 0,6870 |
|  |  |  |  |  |  |  |  |  |  |  |  |
| 11732538_at | TBX21 | 6,17 | 10,07 | 3,9029 | **0,0006** | **0,0227** | 6,60 | 6,14 | -0,4664 | 0,4048 | 0,9689 |
|  |  |  |  |  |  |  |  |  |  |  |  |
| 11749141_a_at | TRAF5 | 11,01 | 10,66 | -0,3562 | 0,3959 | 0,7369 | 10,50 | 11,21 | 0,7085 | **0,0100** | 0,6471 |
| 11728512_a_at | TRAF5 | 9,72 | 9,15 | -0,5737 | 0,0310 | 0,2374 | 9,64 | 10,42 | 0,7832 | **0,0154** | 0,6870 |
|  |  |  |  |  |  |  |  |  |  |  |  |
| 11762375_a_at | FCRL5 | 7,88 | 7,83 | -0,0582 | 0,9452 | 0,9845 | 7,98 | 8,70 | 0,7201 | 0,4846 | 0,9689 |
| 11755780_a_at | FCRL5 | 8,44 | 7,86 | -0,5794 | 0,3858 | 0,7295 | 7,96 | 8,45 | 0,4960 | 0,4784 | 0,9689 |
| 11727230_a_at | FCRL5 | 7,45 | 7,45 | -0,0016 | 0,9978 | 0,9995 | 8,00 | 8,52 | 0,5182 | 0,5599 | 0,9739 |
| 11747768_a_at | FCRL5 | 8,25 | 8,03 | -0,0016 | 0,9978 | 0,9995 | 8,21 | 8,42 | 0,2167 | 0,7904 | 0,9917 |

**Table S4**

Proteins to which there was a significantly different reactivity between antibodies produced by CD43-CD27+ B cells and by CD43+CD27+ B cells (at the nominal 0.05 level of the univariate test). Differentially reactive antigens were determined using class comparison analyses (BRB-Array Tools) at the significance thresholds for univariate tests of p≤0.05 (two-sample T-test with random variance model) and minimum fold changes of 1.5 between groups. Average spot intensities of duplicate spots were used for calculations. For each B cell population, the 15 most significant targets are represented. The table shows the UnigeneID, p-values, False Discovery Rates (FDR), geometric mean intensities, fold changes, Clone IDs, and the gene symbol.

| **Unigene ID** | **Parametric p-value** | **FDR** | **Geom mean of intensities in CD43-CD27+ cells** |  | **Geom mean of intensities in CD43+CD27- cells** | **Fold-change** | **CLONE ID** | **Gene Symbol** |
| --- | --- | --- | --- | --- | --- | --- | --- | --- |
| Hs.517815 | 5,32E-05 | 0,275 | 1575,99 |  | 17397,25 | 0,091 | RZPDp9027N055Q | CHCHD4 |
| Hs.79387 | 8,88E-05 | 0,278 | 1210,15 |  | 17835,27 | 0,068 | RZPDp9027I172Q | PSMC5 |
| Hs.467084 | 0,0001305 | 0,278 | 1670,78 |  | 17577,54 | 0,095 | RZPDp9027O1716Q | EIF4G3 |
| Hs.490789 | 0,0001358 | 0,278 | 1822,05 |  | 14582,53 | 0,12 | RZPDp9027B042Q | PTPRN2 |
| Hs.528581 | 0,0001828 | 0,278 | 1127,19 |  | 6474,74 | 0,17 | RZPDp9028I0813Q | DNLZ |
| Hs.631550 | 0,0001976 | 0,278 | 601,6 |  | 3890,63 | 0,15 | RZPDp9028C2116Q | ARHGEF1 |
| Hs.524210 | 0,0002597 | 0,309 | 1063,58 |  | 17446,2 | 0,061 | RZPDp9027J214Q | ING4 |
| Hs.123198 | 0,0003623 | 0,331 | 817,71 |  | 3678,28 | 0,22 | RZPDp9027K243Q | MYO9B |
| Hs.529449 | 0,0003871 | 0,331 | 2041,26 |  | 18182,04 | 0,11 | RZPDp9027M042Q | APBB3 |
| Hs.497399 | 0,0004061 | 0,331 | 1825,31 |  | 14983,34 | 0,12 | RZPDp9027G101Q | ARL8A |
| Hs.1420 | 0,0004336 | 0,336 | 857,29 |  | 4557,9 | 0,19 | RZPDp9027L0717Q | FGFR3 |
| Hs.524210 | 0,0007127 | 0,371 | 1123,56 |  | 22627,12 | 0,05 | RZPDp9027K061Q | ING4 |
| Hs.655012 | 0,0007172 | 0,371 | 880,22 |  | 14319,77 | 0,061 | RZPDp9028L105Q | GIPC1 |
| Hs.20157 | 0,0007738 | 0,371 | 1626,71 |  | 9609,32 | 0,17 | RZPDp9028I0516Q | CDK5RAP3 |
| Hs.635 | 0,0008113 | 0,371 | 1293,81 |  | 4272,5 | 0,3 | RZPDp9027L223Q | CACNB1 |
|  |  |  |  |  |  |  |  |  |
| Hs.137516 | 0,00104 | 0,371 | 2061,98 |  | 502,27 | 4,11 | RZPDp9028E067Q | FIGNL1 |
| Hs.23650 | 0,0010238 | 0,371 | 2086,54 |  | 490,27 | 4,26 | RZPDp9027K071Q | MAZ |
| Hs.108371 | 0,0008272 | 0,371 | 1799,1 |  | 439,84 | 4,09 | RZPDp9028B017Q | E2F4 |
| Hs.505802 | 0,0007865 | 0,371 | 1603,19 |  | 399,5 | 4,01 | RZPDp9027N1319Q | POLDIP3 |
| Hs.443976 | 0,0007032 | 0,371 | 3452,41 |  | 670,94 | 5,15 | RZPDp9028P168Q | CEP250 |
| Hs.459095 | 0,0005862 | 0,371 | 2196,85 |  | 401,79 | 5,47 | RZPDp9028A068Q | IL16 |
| Hs.150837 | 0,0005569 | 0,371 | 1273,63 |  | 288,73 | 4,41 | RZPDp9028C078Q | TXNDC5 |
| Hs.515662 | 0,0005563 | 0,371 | 1412,41 |  | 276,46 | 5,11 | RZPDp9027A1413Q | ZBTB45 |
| Hs.150549 | 0,0005126 | 0,371 | 1377,04 |  | 375,94 | 3,66 | RZPDp9027A1515Q | GPRIN1 |
| Hs.524271 | 0,0003853 | 0,331 | 1854,91 |  | 402,92 | 4,6 | RZPDp9028B047Q | PHC2 |
| Hs.632403 | 0,0002993 | 0,309 | 1490,23 |  | 284,25 | 5,24 | RZPDp9028G222Q | B4GALT2 |
| Hs.696194 | 0,0002715 | 0,309 | 4614,26 |  | 720,92 | 6,4 | RZPDp9028B1210Q | PARP14 |
| Hs.443625 | 0,0001322 | 0,278 | 3889,08 |  | 505,3 | 7,7 | RZPDp9028K215Q | COL3A1 |
| Hs.694847 | 5,11E-05 | 0,275 | 5626,65 |  | 571,91 | 9,84 | RZPDp9028D217Q | BAZ1B |
| Hs.590999 | 4,50E-06 | 0,0698 | 8875,97 |  | 408,5 | 21,73 | RZPDp9028O197Q |  |

**Table S5: IgA and IgG secretion for serotypes PS-1 and PS-4 by different B cell populations after vaccination with Pneumo23 (indicated with *) or Pneumovax.**

|  | IgA ASC (spots/100000 cells) | | | | IgG ASC (spots/100000 cells) | | | |
| --- | --- | --- | --- | --- | --- | --- | --- | --- |
|  | **CD27+**  **CD43-** | **CD27+**  **CD43+** | **CD27-**  **CD43-** | **CD27-**  **CD43+** | **CD27+**  **CD43-** | **CD27+**  **CD43+** | **CD27-**  **CD43-** | **CD27-**  **CD43+** |
| **PPV - PS-1** | | | | | | | | |
| HV7 7d* | 5 | 126 | 5 | 155 | 0 | 111 | 5 | 21 |
| HV8 7d* | 0 | 344 | 0 | 35 | 0 | 667 | 0 | 15 |
| HV9 7d | 0 | 1067 | 0 | 0 | 0 | 178 | 0 | 0 |
| HV10 7d | 0 | 302 | 0 | 1 | 5 | 453 | 0 | 0 |
| HV11 10d | 0 | 258 | 0 | 2 | 0 | 320 | 0 | 1 |
| HV12 10d | 0 | 507 | 0 | 0 | 0 | 747 | 0 | 2 |
| **PPV - PS-4** | | | | | | | | |
| HV7 7d* | 0 | 366 | 5 | 150 | 0 | 116 | 0 | 21 |
| HV8 7d* | 3 | 895 | 2 | 0 | 5 | 500 | 0 | 5 |
| HV9 7d | 0 | 667 | 0 | 0 | 0 | 89 | 0 | 0 |
| HV10 7d | 0 | 880 | 0 | 2 | 0 | 533 | 0 | 1 |
| HV11 10d | 0 | 267 | 0 | 0 | 0 | 151 | 0 | 0 |
| HV12 10d | 0 | 1600 | 0 | 4 | 0 | 276 | 0 | 2 |

Healthy volunteers (HV) (students) (n=6) were vaccinated with Pneumo23 (indicated with *) or Pneumovax and the presence of vaccine-specific ASC was analyzed by ELISPOT 7-10 days post-vaccination (as indicated).
